# Upregulation of mitochondrial ATPase inhibitory factor 1 (ATPIF1) mediates increased glycolysis in mouse hearts

**DOI:** 10.1172/JCI155333

**Published:** 2022-05-16

**Authors:** Bo Zhou, Arianne Caudal, Xiaoting Tang, Juan D. Chavez, Timothy S. McMillen, Andrew Keller, Outi Villet, Mingyue Zhao, Yaxin Liu, Julia Ritterhoff, Pei Wang, Stephen C. Kolwicz, Wang Wang, James E. Bruce, Rong Tian

**Affiliations:** 1 Mitochondria and Metabolism Center, Department of Anesthesiology & Pain Medicine, and; 2 Department of Genome Sciences, University of Washington, Seattle, Washington, USA.

**Keywords:** Cardiology, Metabolism, Heart failure, Mitochondria, Structural biology

## Abstract

In hypertrophied and failing hearts, fuel metabolism is reprogrammed to increase glucose metabolism, especially glycolysis. This metabolic shift favors biosynthetic function at the expense of ATP production. Mechanisms responsible for the switch are poorly understood. We found that inhibitory factor 1 of the mitochondrial F_o_F_1_-ATP synthase (ATPIF1), a protein known to inhibit ATP hydrolysis by the reverse function of ATP synthase during ischemia, was significantly upregulated in pathological cardiac hypertrophy induced by pressure overload, myocardial infarction, or **α**-adrenergic stimulation. Chemical cross-linking mass spectrometry analysis of hearts hypertrophied by pressure overload suggested that increased expression of ATPIF1 promoted the formation of F_o_F_1_-ATP synthase nonproductive tetramer. Using ATPIF1 gain- and loss-of-function cell models, we demonstrated that stalled electron flow due to impaired ATP synthase activity triggered mitochondrial ROS generation, which stabilized HIF1**α**, leading to transcriptional activation of glycolysis. Cardiac-specific deletion of ATPIF1 in mice prevented the metabolic switch and protected against the pathological remodeling during chronic stress. These results uncover a function of ATPIF1 in nonischemic hearts, which gives F_o_F_1_-ATP synthase a critical role in metabolic rewiring during the pathological remodeling of the heart.

## Introduction

Mitochondrial F_o_F_1_-ATP synthase, or complex V, is composed of a soluble catalytic F_1_ region (main subunit composition α_3_β_3_γ) in the mitochondrial matrix and a membrane-embedded F_o_ region (1). The enzyme complex catalyzes ATP synthesis using the mitochondrial proton gradient generated by the electron transport chain (1). When the proton gradient is compromised, F_o_F_1_-ATP synthase reverses its function from ATP synthesis to ATP hydrolysis so that protons can be pumped into the intermembrane space. Such a response would sustain mitochondrial membrane potential at the expense of cellular ATP level. On the other hand, the reduced proton gradient results in matrix pH acidification, which promotes binding of ATPase inhibitory factor 1 (ATPIF1), a 12 kDa protein molecule, to the F_1_ region of F_o_F_1_-ATP synthase to inhibit its activity, thus slowing ATP hydrolysis (2). Under normal conditions, ATPIF1 was thought not to interact with complex V, thus leaving its ATP synthesis function unaffected. Consistent with this notion, a previous study showed that whole-body deletion of ATPIF1 caused no obvious phenotype in unstressed mice (3).

Our recent studies using cross-linking mass spectrometry have revealed that ATPIF1 exists proximal to the α and β catalytic subunits of F_1_-ATPase in respiring mitochondria, suggesting that ATPIF1 could interact with complex V in ATP synthesis mode (4, 5). In several cancer cell lines, increased expression of ATPIF1 is linked to reduced oxidative phosphorylation (OXPHOS) and increased glycolysis (6). Furthermore, overexpression of a mutant ATPIF1 that could mimic the binding of ATPIF1 with F_o_F_1_-ATP synthase under stress promoted glycolysis in hepatic and neurological tissues in mice even under conditions of normal mitochondrial membrane potential (6–8). These observations collectively raised the possibility that ATPIF1 could interact with complex V in ATP synthesis mode to induce a metabolic switch. How ATPIF1 modulates F_o_F_1_-ATP synthase function under normal pH in respiring mitochondria, and the physiological significance of such an interaction in vivo, remain to be determined.

The heart is a high-energy-consuming organ. Over 95% of the ATP in the heart is generated from mitochondrial OXPHOS. In response to chronic stress, cardiomyocytes develop pathological hypertrophy, which is accompanied by a reprogramming of cardiac metabolism to increased reliance on glycolysis and decreased fatty acid oxidation resulting in reduced ATP production (9, 10). The mechanisms responsible for the switch from oxidative metabolism to glycolysis are poorly understood but appear to be coupled with impaired mitochondrial function (11–13). Interestingly, a recent study showed that whole-body deletion of ATPIF1 preserved cardiac function in a mouse model of pressure overload–induced heart failure (14). While the study did not determine myocardial energy metabolism, it raises the question of whether ATPIF1 plays a role in the metabolic reprogramming of pathological hypertrophy. Here we report that ATPIF1 expression is upregulated in cardiomyocytes and mouse hearts undergoing pathological hypertrophy via an activator protein 1–mediated (AP-1–mediated) transcriptional mechanism. Using genetic models of ATPIF1 gain and loss of function in cardiomyocytes and in mouse hearts, we find that upregulation of ATPIF1 in cardiac hypertrophy inhibits ATP synthesis by promoting tetramer formation of F_o_F_1_-ATP synthase. Impairment of complex V function in respiring mitochondria increased reactive oxygen species (ROS) generation, resulting in transcriptional activation of glycolysis. Cardiac-specific deletion of ATPIF1 in mice prevented the switch to glycolysis in cardiac hypertrophy and protected against cardiac dysfunction during pathological remodeling after myocardial infarction.

## Results

### ATPIF1 was upregulated in pathological cardiac hypertrophy via AP-1–mediated transcriptional activation.

**Figure 1:**
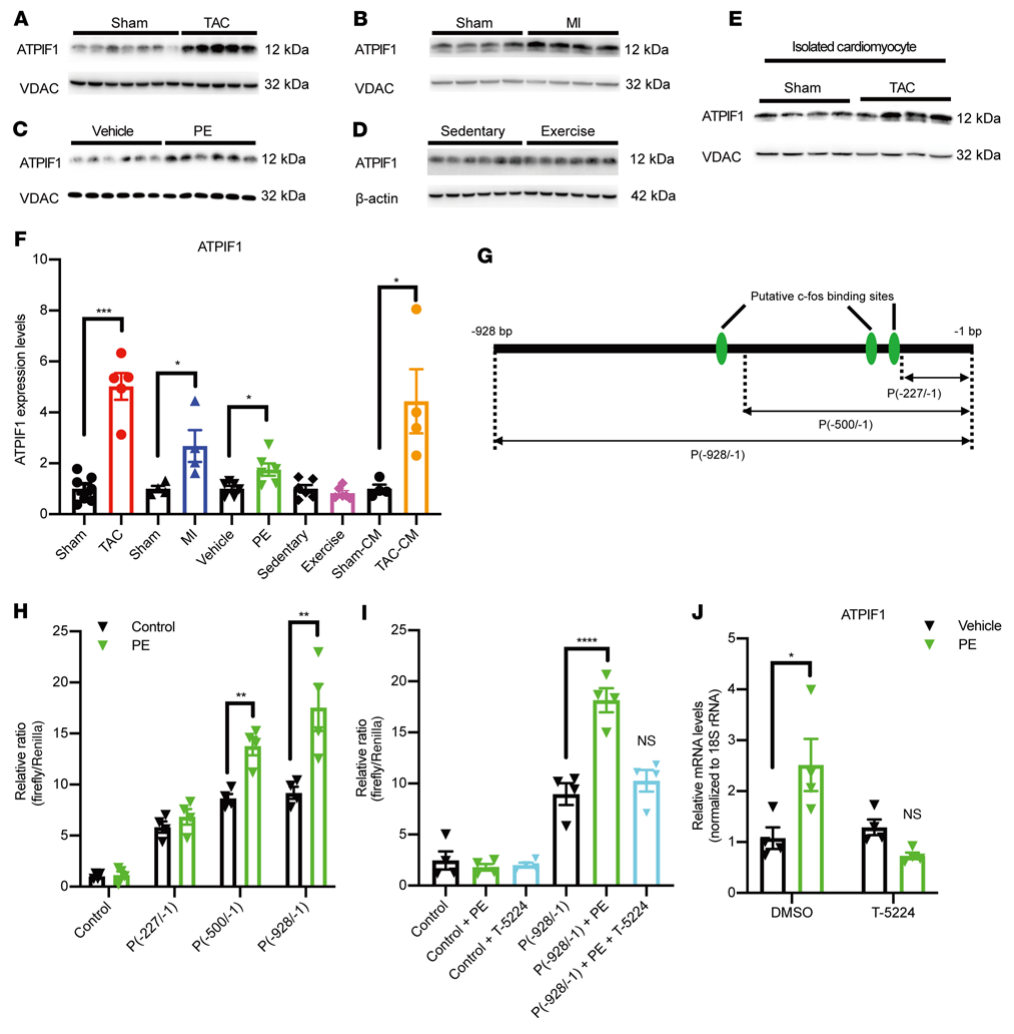
ATPIF1 was upregulated in pathological cardiac hypertrophy. (**A**–**E**) ATPIF1 protein levels were assayed in cardiac mitochondria isolated from mice subjected to TAC (**A**, *n =* 5), left anterior descending artery ligation (MI; **B**, *n =* 4), or sham operations (*n =* 4–7) for 28 days; in primary adult rat cardiomyocytes treated with 10 μM PE for 48 hours (**C**, *n =* 6); in isolated cardiomyocytes from mice subjected to TAC or sham surgery for 28 days (**E**, *n =* 4); and in cardiac tissue lysates prepared from mice subjected to a motorized treadmill 5 times per week for 10 weeks to induce physiological hypertrophy (**D**, *n =* 6). (**F**) Densitometric analysis of ATPIF1 protein levels from each group by ImageJ. (**G**) Schematic of putative c-fos/c-Jun binding sites in mouse ATPIF1 promoter. The predicted binding sites are in the regions of –586/–579 bp, –256/–250 bp, and –235/–228 bp, respectively. Truncated promoter fragments containing decreasing numbers of the binding sites are indicated. (**H** and **I**) Promoter activity in primary adult rat cardiomyocytes carrying dual-luciferase reporters fused with mouse ATPIF1 promoter sequence. Treatment with or without 20 μM T-5224 (AP-1 activity inhibitor) or 10 μM PE is indicated [NS vs. P(–928/–1 bp), *n =* 4]. (**J**) ATPIF1 mRNA expression in adult rat cardiomyocytes receiving indicated treatment (NS vs. vehicle treated with DMSO, *n =* 4). Data are means ± SEM of the values. *P* values were determined using unpaired 2-tailed Student’s *t* test comparing the treatment group with its respective controls (**F** and **H**), 1-way ANOVA followed by Dunnett’s multiple-comparison test (**I**), or 2-way ANOVA followed by Tukey’s multiple-comparison test (**J**); *P < 0.05, **P < 0.01, ***P < 0.001, ****P < 0.0001.

We examined ATPIF1 levels in 3 commonly used models of pathological cardiac hypertrophy: (a) pressure overload via transverse aortic constriction (TAC) in mice; (b) myocardial infarction (MI) by left anterior descending artery ligation in mice; and (c) adult rat ventricular cardiomyocytes (ARVCMs) treated with phenylephrine (PE; 10 μM) for 48 hours (Supplemental Figure 1, A–C; supplemental material available online with this article; https://doi.org/10.1172/JCI155333DS1). In all 3 models, ATPIF1 was markedly upregulated at the mRNA and protein levels in cardiac tissue or cardiomyocytes (Figure 1, A–C, E, and F, and Supplemental Figure 1D). In contrast, the expression of ATPIF1 did not change in the mouse hearts with physiological hypertrophy induced by exercise (Figure 1, D and F, and Supplemental Figure 1, A, B, and D). Thus, upregulation of ATPIF1 appears to be associated with pathological cardiac hypertrophy. To search for the transcriptional mechanisms responsible for ATPIF1 upregulation, the mouse ATPIF1 promoter region (–928/–1 bp) was analyzed by PROMO algorithm (http://alggen.lsi.upc.es/cgi-bin/promo_v3/promo/promoinit.cgi?dirDB=TF_8.3). We found 3 putative binding sites for c-fos and c-Jun, AP-1 transcription factor proteins, in the region of –586/–579 bp, –256/–250 bp, and –235/–228 bp, respectively (Figure 1G and Supplemental Figure 1F). Also, c-fos expression was upregulated in ARVCMs after PE treatment (Supplemental Figure 1E). We thus hypothesized that binding of AP-1 transcription factor regulated ATPIF1 expression during pathological hypertrophy. To test this, we fused the full-length promoter region (–928/–1 bp) or truncated promoter fragments (–500/–1 bp and –227/–1 bp, respectively) to a dual-luciferase reporter gene construct and expressed them in ARVCMs using adenovirus vectors. Our results showed that luciferase activity in PE-treated ARVCMs was dose-dependently correlated with the presence of AP-1 binding sites in the promoter fragment (Figure 1, G and H). Furthermore, the AP-1 inhibitor T-5224 could abolish the increases in luciferase activity and ATPIF1 expression induced by PE (Figure 1, I and J). Taken together, these data suggested that ATPIF1 upregulation was mediated by the pro-growth transcription factor AP-1 in pathological cardiac hypertrophy.

### ATPIF1 promoted glycolysis in cardiomyocytes through HIF1α signaling.

**Figure 2:**
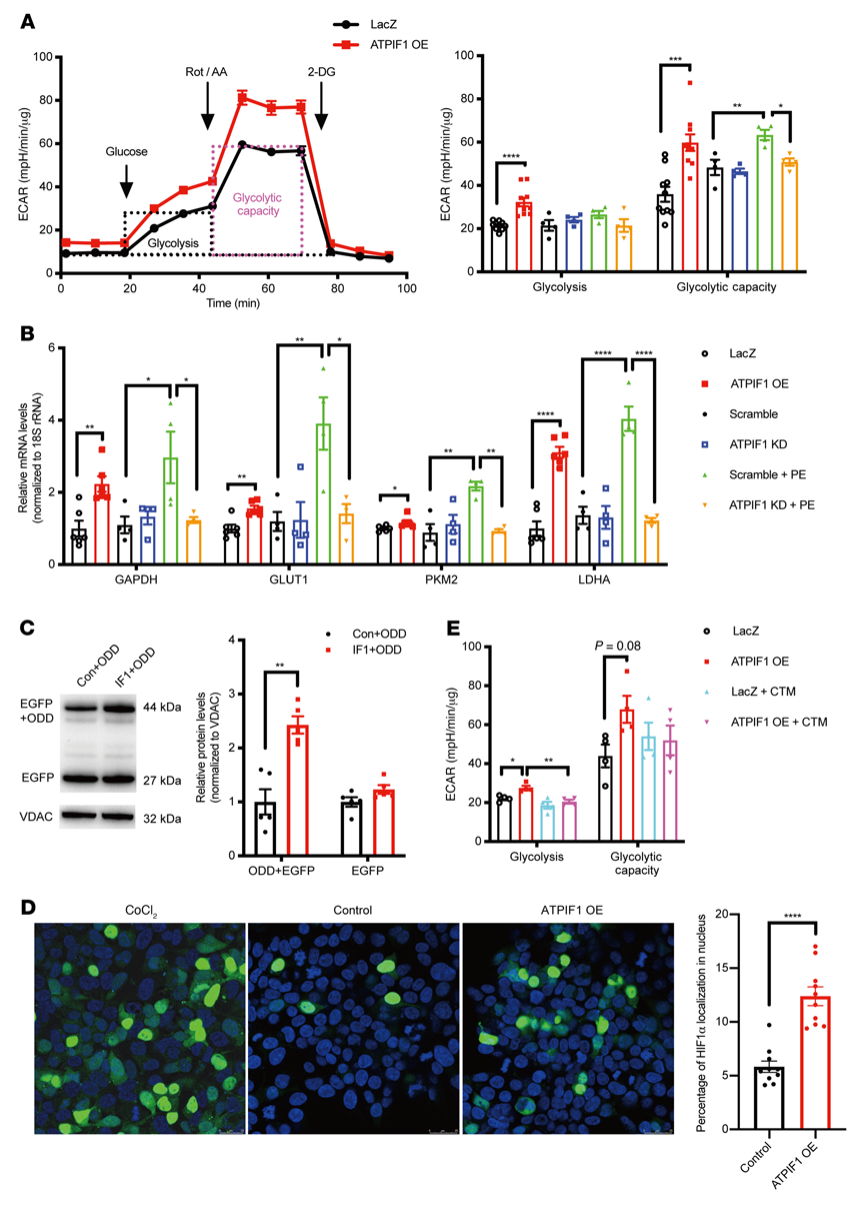
ATPIF1 promoted glycolysis in cardiomyocytes through HIF1α signaling. (**A** and **B**) Extracellular acidification rate (ECAR; **A**) and glycolytic gene expression (**B**) analysis of cardiomyocytes with overexpression (*n =* 6–10; LacZ as control) or knockdown of ATPIF1 treated with or without PE (*n =* 4; Scramble as control). Rot, rotenone; AA, antimycin A; 2-DG, 2-deoxyglucose; mpH, milli-pH. (**C**) Representative immunoblot analysis of EGFP level in HEK293 cells cotransfected with ATPIF1 and ODD-EGFP for 24 hours (*n =* 5). (**D**) Percentage and representative immunostaining image of HIF1α localization to the nucleus in HEK293 cells cotransfected with ATPIF1 and HIF1α-EGFP for 24 hours (*n =* 10); scale bars: 25 μm. (**E**) ECAR analysis of cardiomyocytes with or without the addition of HIF1α inhibitor (chetomin, CTM) in the presence of ATPIF1 (*n =* 4). All values were normalized to 18S rRNA expression and to control sets (**B**). Data are means ± SEM of the values. Comparison of LacZ vs. ATPIF1 OE was made using unpaired 2-tailed Student’s *t* test. Comparisons for Scramble vs. ATPIF1 KD vs. Scramble+PE vs. ATPIF1 KD+PE were made using 2-way ANOVA followed by Tukey’s multiple-comparison test; *P < 0.05, **P < 0.01, ***P < 0.001, ****P < 0.0001.

To understand the functional role of ATPIF1 upregulation in hypertrophied cardiomyocytes, we sought to overexpress (OE; 10 MOI) or knock down (KD; 100 MOI) ATPIF1 in ARVCMs by adenoviral vectors and subjected them to PE treatment (Supplemental Figure 2A). We found that ATPIF1 OE increased glycolysis and the maximal capacity of glycolysis in ARVCMs (Figure 2A). Moreover, PE-induced upregulation of the glycolytic capacity was abrogated by ATPIF1 KD (Figure 2A). Similarly, the extracellular lactate levels were significantly increased by ATPIF1 OE or PE treatment, and reduced by ATPIF1 KD in PE-treated ARVCMs (Supplemental Figure 2B). These results suggested that ATPIF1 upregulation was sufficient and necessary to trigger increased glycolysis in hypertrophied cardiomyocytes. ATPIF1 OE or pathological hypertrophy induced by PE increased the expression of glycolytic enzymes, e.g., GAPDH, GLUT1, LDHA, and PKM2, and the PE-induced changes were abolished by ATPIF1 KD (Figure 2B). Consistently, the activity of GAPDH and LDH was higher in the ATPIF1-OE group (Supplemental Figure 2, C and D). The increased LDH activity induced by PE was inhibited by ATPIF1 KD (Supplemental Figure 2D). Combined, these findings suggest that upregulation of ATPIF1 promotes glycolysis via transcriptional mechanisms.

It has been shown that HIF1α is a potent transcriptional regulator of glycolytic genes, and HIF1α is upregulated in hypertrophied cardiomyocytes (15). We found that ATPIF1 OE increased HIF1α levels, and, importantly, ATPIF1 KD abrogated PE-induced HIF1α accumulation (Supplemental Figure 2E). To determine whether ATPIF1 OE increased HIF1α stability, we coexpressed the oxygen-dependent degradation domain (ODD) of HIF1α and ATPIF1 in HEK293 cells. The ODD was fused with EGFP gene (ODD-EGFP) to monitor the level of expression. Western blots showed high-level enrichment of ODD in the ATPIF1-OE group, while EGFP levels were comparable to those of the control group (Figure 2C), which suggested that ATPIF1 OE could prevent ODD degradation. In addition, confocal imaging and immunoblot analysis showed that HIF1α-EGFP, when cotransfected with ATPIF1 in HEK293 cells, displayed increased nuclear localization (Figure 2D and Supplemental Figure 2F). These findings suggested that upregulation of ATPIF1 led to stabilization and subsequent nuclear translocation of HIF1α, which was essential for the activation of target genes. To further substantiate the link between HIF1α and increased glycolysis induced by ATPIF1, we used chetomin (CTM) to inhibit HIF1α transcriptional activity and examined glycolysis rate and glycolytic gene expression levels in ATPIF1-OE cardiomyocytes. CTM treatment prevented the increase in glycolysis and normalized the expression of glycolytic genes (Figure 2E and Supplemental Figure 2, G and H). These observations collectively suggested that HIF1α signaling mediated the increased glycolysis caused by ATPIF1 upregulation.

### Increased mitochondrial ROS level was necessary for ATPIF1-triggered HIF1α activation.

**Figure 3:**
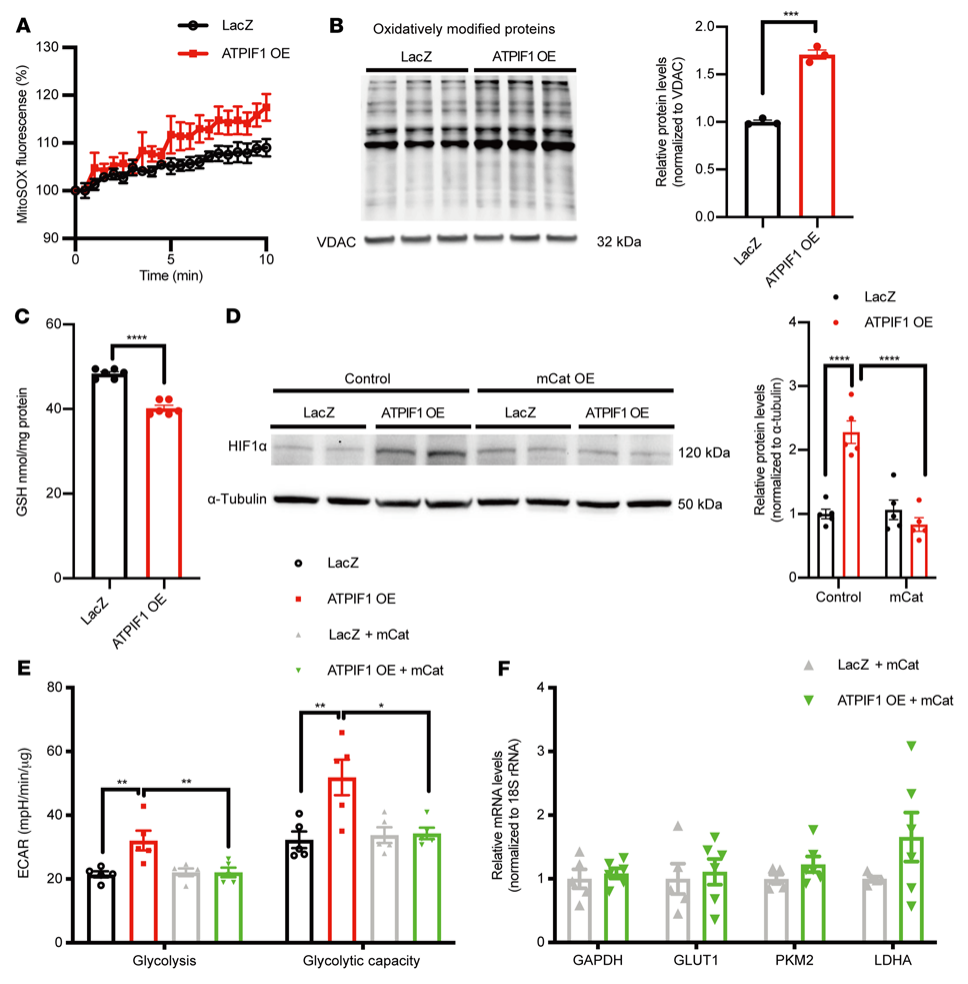
Increased mtROS level was necessary for ATPIF1-triggered HIF1α activation. (**A**) Real-time measurement of mitochondrial superoxide production in cardiomyocytes by MitoSOX (*n =* 4). (**B**) Immunoblot analysis of protein carbonyls in isolated mitochondria from cardiomyocytes (*n =* 3). (**C**) Quantification of glutathione (GSH) levels in the cardiomyocyte lysates (*n =* 6). (**D**–**F**) Representative immunoblot and group average of HIF1α protein level (**D**, *n =* 5), ECAR measurement (**E**, *n =* 5), and real-time PCR analysis of glycolytic gene expression (**F**, *n =* 6) of cardiomyocytes with coexpression of ATPIF1 and mitochondrial catalase (mCat). Gene expression values were normalized to 18S rRNA expression and are shown as relative change over the average of control sets (**F**). Data are means ± SEM of the values. *P* values were determined using unpaired 2-tailed Student’s *t* test (**C** and **F**) or 2-way ANOVA followed by Tukey’s multiple-comparison test (**D** and **E**); *P < 0.05, **P < 0.01, ***P < 0.001, ****P < 0.0001.

ATPIF1 upregulation in cardiomyocytes and H9C2 cells induced a significant increase in mitochondrial superoxide measured by MitoSOX fluorescence (Figure 3A and Supplemental Figure 3A). We also found that ATPIF1 OE increased hydrogen peroxide levels in H9C2 cells (Supplemental Figure 3B). Protein carbonylation, a readout of oxidative modification by ROS, was higher in isolated mitochondria from ATPIF1-OE cardiomyocytes (Figure 3B). Increased cellular oxidative stress was also evidenced by a lower level of glutathione in ATPIF1-OE (Figure 3C). As mitochondrial ROS (mtROS) have been proposed as a signaling mechanism for HIF1α stabilization (16), we next tested whether scavenging of mtROS could prevent the increases in HIF1α levels caused by ATPIF1 OE. Expressing mitochondria-localized catalase (mCat) normalized HIF1α level in ATPIF1-OE cardiomyocytes (Figure 3D). Scavenging of mtROS restored the glycolysis rate and prevented the upregulation of glycolytic genes induced by ATPIF1 (Figure 3, E and F, and Supplemental Figure 3C). Similar effects could also be achieved by a ROS scavenger, NAC (*N*-acetyl-l-cysteine), in ATPIF1-OE cardiomyocytes (Supplemental Figure 3, D–G). Collectively, these results suggested that upregulation of ATPIF1 induced HIF1α accumulation via elevation of mtROS.

### Overexpression of ATPIF1 inhibited complex V activity and slowed electron flow through the electron transport chain.

**Figure 4:**
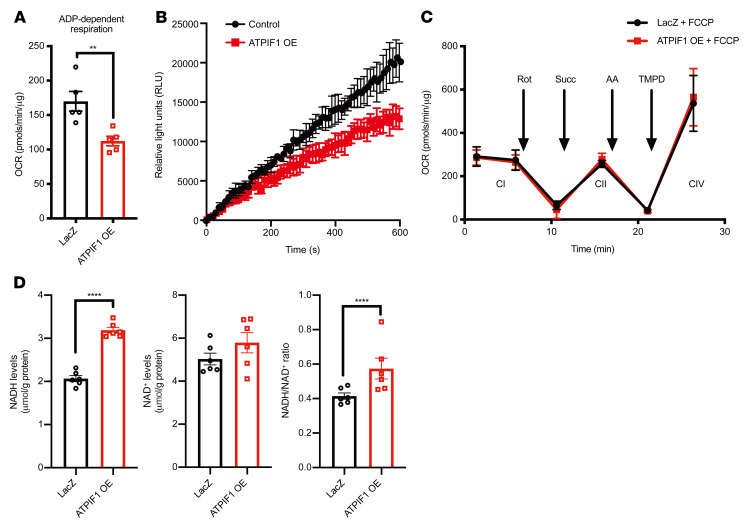
ATPIF1 overexpression inhibited complex V activity and slowed electron flow through the ETC. (**A**) Measurement of ADP-dependent OCR in isolated mitochondria from cardiomyocytes by Seahorse analyzer using pyruvate and malate as substrates (*n =* 5). (**B**) Rate of ATP production in cardiomyocytes (*n =* 4). (**C**) Measurements of OCR driven by FCCP to assess complex I–IV activities in isolated mitochondria from cardiomyocytes using Seahorse analyzer (*n =* 4). Rotenone was added to inhibit complex I activity, and succinate was used to activate complex II–mediated respiration. Antimycin A was added to inhibit complex III activity, and complex IV was stimulated by the addition of TMPD and ascorbate. (**D**) Quantification of NAD^+^ and NADH levels in the cardiomyocyte lysates (*n =* 6). Data are means ± SEM of the values. Comparisons between ATPIF1 OE and its respective controls were made using unpaired 2-tailed Student’s *t* test; **P < 0.01, ****P < 0.0001.

When mitochondrial membrane potential is lost during stress, F_o_F_1_-ATP synthase functions in the direction of ATP hydrolysis pumping protons from the matrix to the intermembrane space to aid restoration of membrane potential (1). The binding of ATPIF1 and complex V inhibits F_o_F_1_-ATPase activity under these conditions to preserve ATP (2). Consistent with this notion, ATPIF1 OE reduced the rate of ATP hydrolysis when mitochondrial membrane potential was compromised, as evidenced by decreased release of Mg^2+^ from hydrolyzed ATP (Supplemental Figure 4A). Interestingly, ATPIF1 OE in cardiomyocytes also triggered a substantial decrease of mitochondrial respiratory rates driven by ADP (state 3 respiration), suggesting that a high level of ATPIF1 inhibited F_o_F_1_-ATP synthase when it functioned in the direction of ATP synthesis (Figure 4A and Supplemental Figure 4B). Indeed, the ATP production rate was significantly inhibited in cardiomyocytes overexpressing ATPIF1 (Figure 4B). The inhibition appeared to be specific to complex V, because mitochondrial respiration driven by FCCP, which bypasses complex V, was unaltered by ATPIF1 OE (Figure 4C). No changes in the function of other electron transport chain (ETC) complexes were found during FCCP-stimulated respiration (Figure 4C). Moreover, we also ruled out changes in mitochondrial ETC protein expression and supercomplex levels as potential causes of reduced OCR in cardiomyocytes with ATPIF1 OE (Supplemental Figure 4, C and D). Inhibition of OXPHOS would stall electron flow on the ETC, as the classic study by Chance and Williams demonstrated (17), hence accumulation of reducing equivalents, such as NADH. We found that NADH level and NADH/NAD^+^ ratio increased in cardiomyocytes with ATPIF1 OE (Figure 4D). Taken together, these results showed that upregulation of ATPIF1 slowed electron flow on the ETC, rendering the microenvironment more reduced and conducive to ROS production.

### Upregulation of ATPIF1 in hypertrophied hearts impaired complex V function by promoting the formation of F_o_F_1_-ATP synthase tetramers.

**Figure 5:**
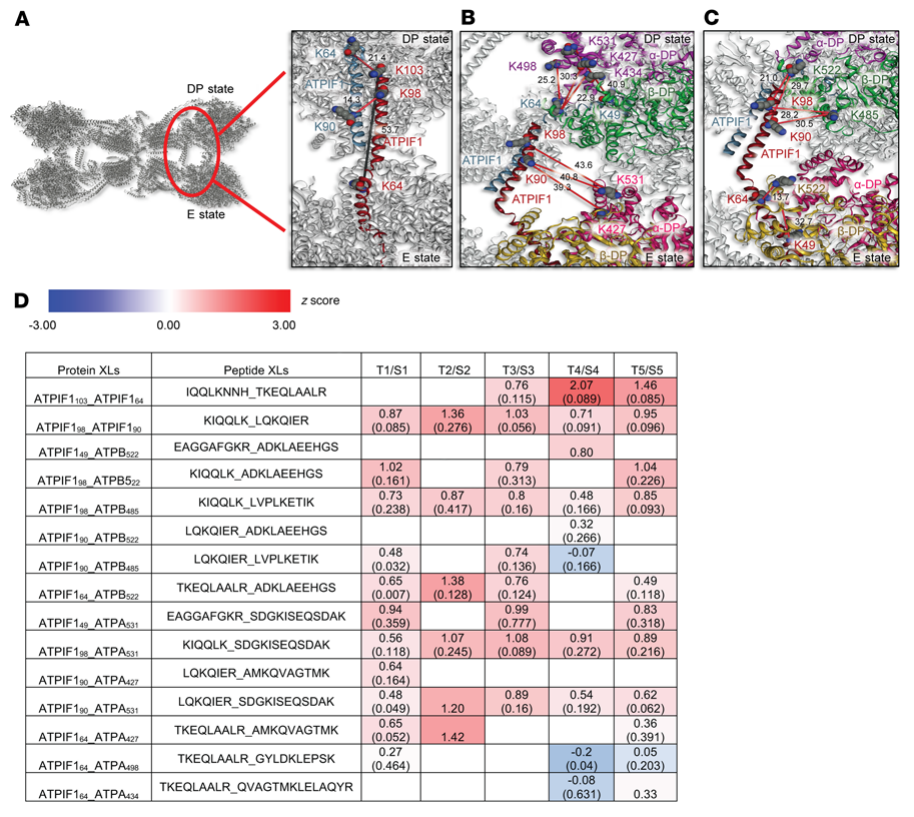
Upregulation of ATPIF1 in hypertrophied hearts promoted the F_o_F_1_-ATP synthase tetramer. (**A**–**C**) ATPIF1-inhibited ATP synthase tetramer structure (PDB: 6j5k) with inset showing cross-linked regions. Cross-linked residues are marked with red-gray-blue spheres and labeled with the same color as the chain name label. Cross-links are indicated in red lines with Cα–Cα Euclidean distance (Å) written in black. (**A**) ATPIF1-ATPIF1 dimers: Cross-link K64-K103 as interlink (red line) between ATPIF1 dimers has smaller Cα–Cα Euclidean distance (21.4 Å) than intralink (gray line) within ATPIF1 monomer (53.7 Å), supporting antiparallel ATPIF1 dimer structure. (**B**) ATPIF1-ATPA (α subunit): N-terminal region of ATPIF1 was linked to α_DP_ in DP-state F_1_-ATPase, while C-terminal region from the other ATPIF1 was linked to α_DP_ in E-state F_1_-ATPase. (**C**) ATPIF1-ATPB (β subunit): N-terminal region of ATPIF1 was linked to β_DP_ in E-state of F_1_-ATPase, while C-terminal region of the same ATPIF1 was linked to β_DP_ in DP-state F_1_-ATPase. (**D**) Log_2_ value of TAC/sham (T/S) ratios from 5 biological replicates for cross-links (XLs) identified between ATPIF1 dimer, ATPIF1_ATPA, and ATPIF1_ATPB as shown in **A**–**C**. The 95% confidence interval for each cross-linked peptide pair is shown in parentheses.

A recent cryogenic electron microscopy (cryo-EM) study demonstrated a nonproductive tetrameric state of F_o_F_1_-ATP synthase when bound with natural inhibitor ATPIF1 (18). To determine whether upregulation of ATPIF1 increased F_o_F_1_-ATP synthase tetramer formation in hypertrophied hearts, we applied isobaric quantitative Protein Interaction Reporter (iqPIR) cross-linking technologies to TAC and sham-operated hearts and then isolated their mitochondria for mass spectrometry analysis (19). A total of 15 nonredundant cross-links were identified involving ATPIF1, including 2 pairs for ATPIF1, 7 pairs for ATPIF1-ATPA, and 6 pairs for ATPIF1-ATPB, from 5 biological replicates of TAC/sham sample pairs. Our cross-link data show a high degree of agreement with the F_o_F_1_-ATP synthase tetramer structure (6j5k, Protein Data Bank [PDB]; https://www.rcsb.org/) with all 15 links mapped within the spannable distance of PIR cross-linkers (Figure 5). The link between ATPIF1 K64 and K103 is consistent with an interlink between antiparallel ATPIF1-ATPIF1 dimers (Cα–Cα Euclidean distance 21.4 Å) and beyond the linkable distance for an intralink across an elongated ATPIF1 monomer) (distance 53.7 Å, gray line) as shown in Figure 5A. Consistent with previous findings that the N-terminus of ATPIF1 is inserted into the F_1_ head interacting with the ATPA-ATPB α_DP_-β_DP_ interface (2, 20), we identified cross-links both between the N-terminal region of ATPIF1 and ATPA α_DP_ chain (ATPIF1_49__ATPA_531_, ATPIF1_64__ATPA_427_, ATPIF1_64__ATPA_434_, and ATPIF1_64__ATPA_498_ in Figure 5B) and between N-terminal ATPIF1 and ATPB β_DP_ chain (ATPIF1_49__ATPB_522_ and ATPIF1_64__ATP_522_ in Figure 5C). Interestingly, we also detected the C-terminal region of ATPIF1 (K90 and K98) cross-linked to the same lysine sites K427 and K531 in ATPA. On the basis of all available structures containing complex V and ATPIF1, these links are consistent only with the C-terminal domain of ATPIF1 linked with an F_1_ protomer other than that which interacts with the ATPIF1 N-terminal region. For instance, these links, when mapped to the PDB 6j5k structure of ATPIF1-inhibited ATP synthase tetrameric species, appear consistent with C-terminal K90 and K98 from one ATPIF1 monomer linked to K427 and K531 in α_DP_ chain in the E-state protomer, while the N-terminal K49 and K64 from the other ATPIF1 monomer linked to K427, K434, K498, and K531 in the α_DP_ chain in the DP-state protomer (Figure 5B). Similarly, while the N-terminal ATPIF1 K49 and K64 were linked to K522 in the β_DP_ chain in the E-state protomer, the C-terminal K90 and K98 from the other elongated end of the same ATPIF1 chain were linked to β_DP_ chain (K498 and K522) in the DP-state F_1_ (Figure 5C). The simultaneous detection of these ATPIF1 cross-links with α_DP_-β_DP_ in both DP state and E state of F_o_F_1_-ATP synthase protomers indicated the presence of tetrameric F_o_F_1_-ATP synthase structure brought together by 2 antiparallel ATPIF1 dimers.

We performed quantitative analysis of the cross-links by pairwise comparison of TAC and sham-operated hearts (Figure 5D). The ATPIF1-containing cross-links were markedly increased in TAC as compared with sham controls, indicating that ATPIF1 binding with F_o_F_1_-ATP synthase was increased and ATPIF1-inhibited F_o_F_1_-ATP synthase tetrameric nonproductive state was elevated in hypertrophied heart. For comparison, we also included those intralinks in ATPA and ATPB, and ATPA-ATPB interlinks involved with those same lysine residues cross-linked to ATPIF1 (Supplemental Table 1). Nearly all those links showed unchanged or slightly decreased levels in TAC relative to sham, suggesting that F_o_F_1_-ATP synthase levels and conformations were unaltered in comparison of TAC and sham samples. This observation serves as an inherent internal control indicating that the increase of ATPIF1-F_1_ interactions in TAC is unique and not the result of a global increase in all link levels in TAC. Moreover, the increase in ATPIF1-F_1_ links is also consistent with and may result from increased ATPIF1 levels in mitochondria of the TAC group. It is worth noting that several linked lysine residue pairs, e.g., ATPA_427__ATPA_531_, ATPA_434__ATPA_531_, ATPB_522__ATPA_427_, etc., were detected multiple times in different forms, such as peptides containing modifications or missed tryptic cleavage sites. Although these peptide pairs provide redundant lysine linkage information, they provide valuable insight regarding the reliability or variance of the quantitation of link levels. In all cases, these modified or alternate-length species showed highly consistent log_2_ ratios, indicating that our quantitation results were reliable with high confidence. Collectively, these observations suggested that ATPIF1 upregulation in hypertrophied hearts impaired complex V function by promoting the formation of nonproductive F_o_F_1_-ATP synthase tetramers.

### Deletion of ATPIF1 prevented the increase of glycolysis and protected against pathological hypertrophy and dysfunction.

**Figure 6:**
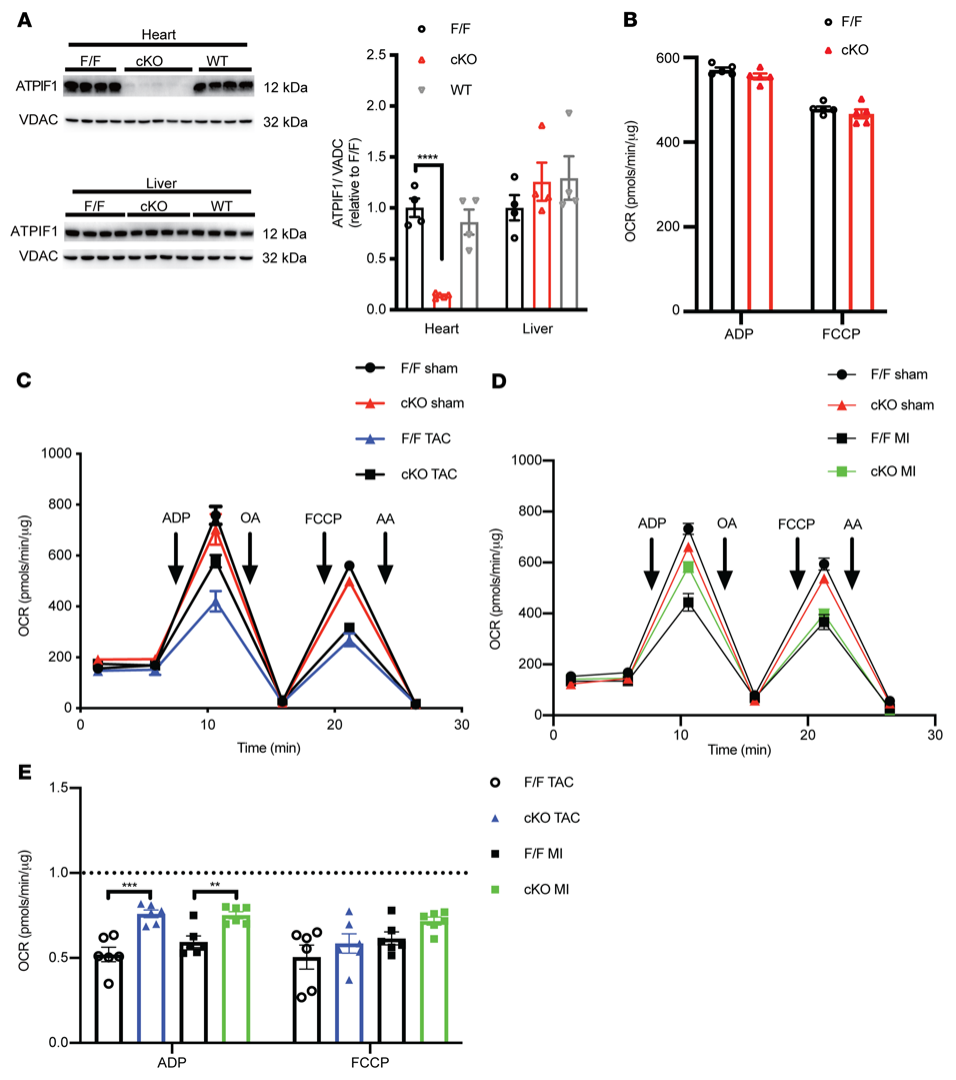
Cardiac-specific deletion of ATPIF1 partially restored respiratory function induced by pathological cardiac hypertrophy. (**A**) Immunoblot of ATPIF1 expression from liver or ventricular lysates (*n =* 4–5). (**B**) Analysis of isolated mouse heart mitochondrial OCR by Seahorse analyzer (*n =* 5). (**C** and **D**) Representative OCR tracing and tabulated data of isolated mouse heart mitochondrial OCR at 28 days after TAC (**C**), MI (**D**), or sham surgery. (**E**) Average OCR of TAC and MI groups during ADP or FCCP stimulation, normalized to the respective sham group (*n =* 6). Data are means ± SEM of the values. *P* values were determined using unpaired 2-tailed Student’s *t* test (**E**) or 1-way ANOVA followed by Dunnett’s multiple-comparison test (**A**); **P < 0.01, ***P < 0.001, ****P < 0.0001.

To investigate the in vivo role of ATPIF1 in hypertrophied hearts, we generated cardiac-specific ATPIF1-knockout (cKO) mice by deleting exon 3 of the *Atpif1* gene (Supplemental Figure 5A). As shown in Figure 6A and Supplemental Figure 5B, ATPIF1 protein and mRNA levels in the heart of cKO mice were reduced by approximately 90% in comparison with nontransgenic (WT) or ATPIF1-floxed (F/F) mice, but ATPIF1 level was unaltered in the liver of cKO mice. Deletion of ATPIF1 did not change mitochondrial respiration rate in unstressed heart (Figure 6B). Cardiac mitochondrial OXPHOS protein and supercomplex levels were maintained, including the α and β catalytic subunits in F_o_F_1_-ATP synthase (Supplemental Figure 5, C and D). These data are consistent with prior reports that whole-body deletion of ATPIF1 caused no obvious phenotypes in unstressed mice, suggesting that ATPIF1 plays a minor role under normal conditions (3). As expected, mitochondrial respiration was impaired in MI or TAC hearts. The decrease of OCR was mitigated in cKO even though the OXPHOS protein levels were not changed (Figure 6, C–E, and Supplemental Figure 5, E and F). These results corroborated the cross-linking finding above and suggested that upregulation of ATPIF1 likely impaired complex V function through an allosteric mechanism.

**Figure 7:**
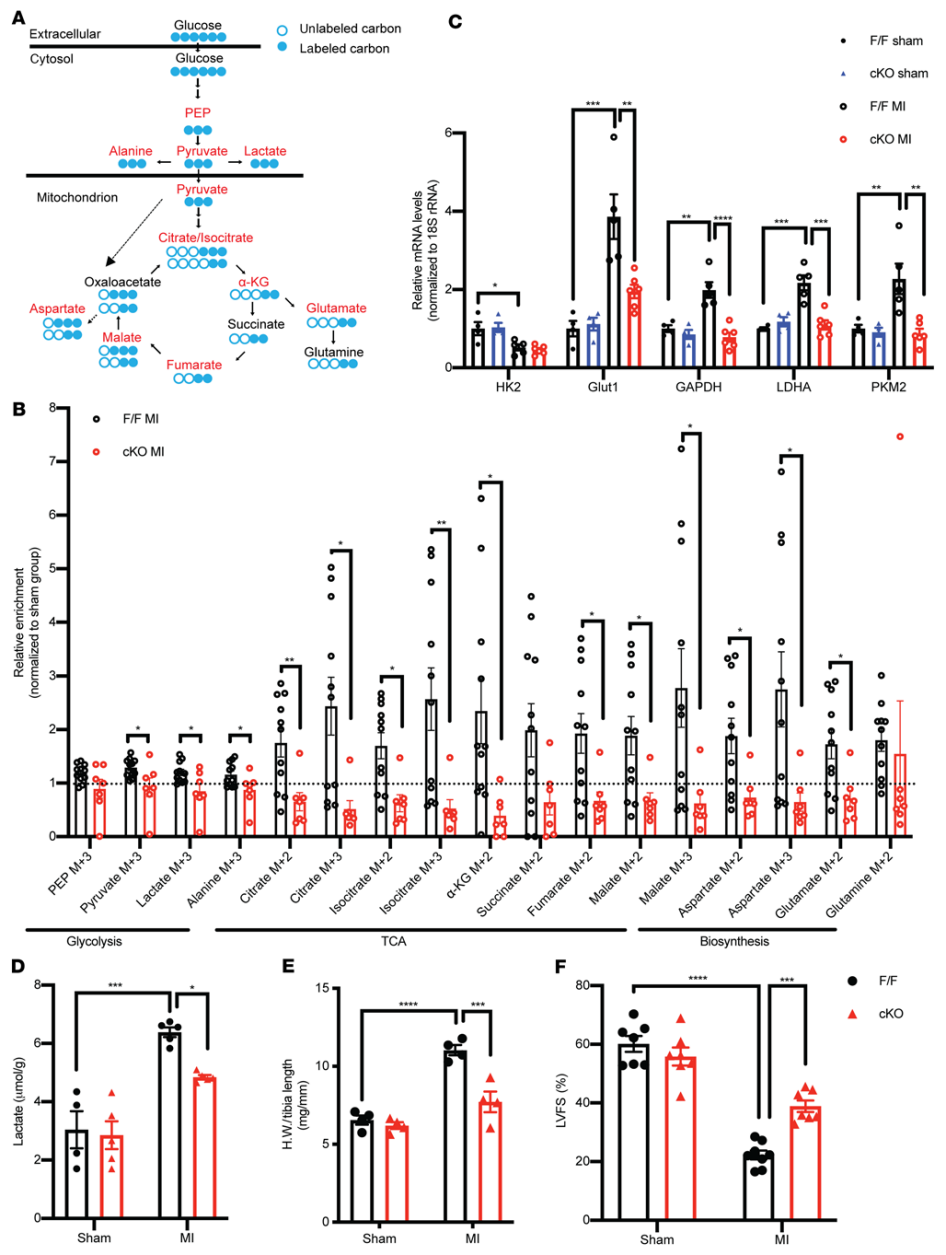
Cardiac-specific deletion of ATPIF1 mitigated metabolic switch and reduced pathological remodeling of the heart after MI. (**A**) Schematics of U-^13^C glucose metabolism via glycolysis and TCA cycle. PEP, phosphoenolpyruvate; α-KG, α-ketoglutarate. (**B**) Relative ^13^C enrichment of glycolysis, TCA cycle, and anaplerosis metabolites in F/F and cKO ventricular tissue at day 7 after MI (*n =* 7–11). The dotted line represents the level of the sham group. (**C** and **D**) Real-time PCR assay of glycolytic genes (**C**) and quantification of lactate levels (**D**) in ventricular lysates 14 days after operation (*n =* 4–6). (**E**) Ratio of heart weight (HW) to tibia length 28 days after operation (*n =* 4). (**F**) Left ventricular fractional shortening (LVFS) assessed by echocardiography 28 days after MI or sham operation (*n =* 7–8). 18S rRNA served as mRNA internal control. Data are means ± SEM of the values. *P* values were determined using unpaired 2-tailed Student’s *t* test (**B**) or 2-way ANOVA followed by Tukey’s multiple-comparison test (**C**–**F**); *P < 0.05, **P < 0.01, ***P < 0.001, ****P < 0.0001.

To ask whether ATPIF1 cKO could inhibit the upregulation of glycolysis in hypertrophied hearts, we first performed a time course study of the relationship between glycolysis and ATPIF1 expression at days 3, 7, and 14 after MI. We found that ATPIF1 protein was significantly increased at days 7 and 14 after MI, and tissue level of lactate, a glycolytic end product, followed a similar pattern (Supplemental Figure 6, A and B). Next, we tracked cardiac glucose metabolism in vivo with U-^13^C glucose at day 7 after MI in mice. Via glycolysis, U-^13^C glucose breaks down to ^13^C-pyruvate, lactate, and alanine (M+3). Pyruvate enters the TCA cycle in mitochondria either as acetyl-CoA (M+2), or as oxaloacetate (M+3) via anaplerosis (Figure 7A). In sham-operated hearts, the ^13^C-labeling pattern was similar between F/F and cKO (Supplemental Figure 6C). Compared with the sham group, flux of glucose into glycolysis, TCA intermediates, or amino acids was significantly increased in the F/F MI group, but the increase was not observed in cKO-MI mice (Figure 7B). Downstream of glycolysis, upregulation of anaplerotic flux and increased synthesis of aspartate and glutamate from glucose have been shown as part of the metabolic reprogramming in hypertrophic hearts required for the cell growth (21, 22). These changes were also mitigated in cKO-MI hearts (Figure 7B). Furthermore, the expression of glucose uptake and glycolytic genes was significantly increased in the myocardium with hypertrophy induced by MI or TAC compared with the respective sham-operated hearts, while the increases were prevented in cKO hearts (Figure 7C and Supplemental Figure 6D). Immunoblotting of heart lysates showed that upregulation of proteins in the glycolysis pathway was reduced in cKO-MI and cKO-TAC hearts (Supplemental Figure 6E). Tissue lactate level was about 2-fold higher in control TAC or MI hearts but was significantly reduced in the cKO hearts (Figure 7D and Supplemental Figure 6F). Cardiac glycogen content was similar between the genotypes, suggesting that it is unlikely that altered glycogen metabolism contributed to the differences in glycolysis in our model (Supplemental Figure 6G).

A previous study showed that whole-body deletion of ATPIF1 protected against TAC-induced cardiac dysfunction in mice (14). Here, we found that cKO demonstrated reduced cardiac hypertrophy and improved cardiac function 4 weeks after MI compared with the F/F control group (Figure 7, E and F). Although α-MHC-Cre mice were not used as controls in this study, it is unlikely that effects associated with Cre expression in cKO could account for the observation, as it would impair rather than improve cardiac function. It has been proposed that ATPIF1 could reduce the rate of ATP hydrolysis during ischemia and thus affect the outcome of MI (23). We, however, observed similar levels of ATP or phosphocreatine in F/F and cKO hearts before or during ischemia (Supplemental Figure 6, H and I). Moreover, ligation of left coronary artery resulted in similar infarct size 3 days after MI in cKO and control mice (Supplemental Figure 6J). Thus, the differential outcome in cKO-MI likely reflected the role of ATPIF1 in post-MI remodeling. Together, these findings substantiated the observations made in ARVCMs and suggested that upregulation of ATPIF1 is a trigger of increased glucose utilization in the hypertrophied heart, which modulated pathological remodeling of the heart through promoting metabolic reprogramming.

## Discussion

The present study demonstrates that upregulation of ATPIF1 contributes to the switch of oxidative metabolism to glycolysis in the heart during the development of pathological hypertrophy. We have found that ATPIF1 inhibits the function of F_o_F_1_-ATP synthase in the direction of ATP synthesis by promoting the formation of nonproductive tetramer structure. Stalled electron flow due to impaired ATP synthesis activity triggers ROS generation in the mitochondria, which stabilizes HIF1α, leading to transcriptional activation of glycolysis. Deletion of ATPIF1 in cardiomyocytes or scavenging mitochondrial ROS prevented the metabolic switch and protected against hypertrophy and dysfunction induced by pathological stress.

ATPIF1 was first identified as an endogenous inhibitor of F_o_F_1_-ATP synthase; binding of ATPIF1 and the β-subunit of F_1_-ATPase blocks the reverse rotation of the ATP synthase to hydrolyze ATP when the proton-pumping function of the ETC is compromised (24, 25). Because the interaction of ATPIF1 and the F_o_F_1_-ATP synthase is pH sensitive, it has long been thought to occur only under conditions of mitochondrial matrix acidification, i.e., hypoxia or inhibition of ETC activity. However, recent studies suggest that ATPIF1 may interact with F_o_F_1_-ATP synthase under normal conditions, although whether and how such interaction affects complex V function is poorly understood (4, 5, 26). Here we show that upregulation of ATPIF1 in the mouse heart inhibits ATP synthesis activity by promoting tetramerization of F_o_F_1_-ATP synthase. A recently published cryo-EM structure of porcine ATP synthase showed a similar configuration of 2 dimers of F_o_F_1_-ATP synthase pulled together by ATPIF1 dimers to form a tetramer in an inhibited state (18). The tetramer of F_o_F_1_-ATP synthase is likely rare under normal conditions, since deletion of ATPIF1 does not affect mitochondrial respiration, bioenergetics, or any cardiac phenotypes in unstressed mice (3, 14). We speculate that the binding of ATPIF1 and F_o_F_1_-ATP synthase is enhanced by the upregulation of ATPIF1 levels in hypertrophied hearts. In addition to pH, a recent study using homo-oligomerization modeling suggests that the fraction of ATPIF1 dimer, the active form that binds the β subunit of F_1_-ATPase, is dependent on protein concentration and cation strength, especially Ca^2+^, in the environment (27). Increased mitochondrial Ca^2+^ influx during the development of hypertrophy could also promote dimerization of ATPIF1 and binding with F_o_F_1_-ATP synthase (28).

It is well documented that cardiac metabolism switches from predominantly fatty acid oxidation (FAO) to increased reliance on glucose during the development of pathological hypertrophy (10, 29). The heart has a high energy demand for contraction, which is matched by ATP production via oxidative phosphorylation in the mitochondria. A switch from oxidative metabolism toward glycolysis impairs the capacity for ATP synthesis (9, 10). The change appears counterintuitive, as cardiac hypertrophy is commonly associated with increased workload for the heart. A number of mechanisms, such as downregulation of PPARα and PGC1α-mediated transcription, have been identified for the reduced FAO in cardiac hypertrophy, but the driver for the upregulation of glycolysis remains elusive. Since FAO has a high capacity for ATP production, it has been hypothesized that increased glycolysis is a compensatory response to energy deficit caused by impaired FAO (10, 30). However, it is puzzling that increased glucose utilization occurs early in cardiac hypertrophy prior to contractile dysfunction or energy deficiency. Moreover, we and others have recently demonstrated that increased glucose metabolism is required to prime mTOR signaling and to provide metabolites for biosynthesis, although increased glucose alone is not sufficient to drive hypertrophic growth of cardiomyocytes (22, 31–34). These observations raise the possibility that increased glucose metabolism is programmed to meet metabolic requirement for pathological hypertrophy. In support of this notion, we find that a transcriptional mechanism mediated by activator protein 1 (AP-1) is responsible for ATPIF1 upregulation in hypertrophied cardiomyocytes. AP-1 activation is a known mechanism for induction of fetal gene expression and cardiomyocyte hypertrophy (35–37). In this way, AP-1–induced upregulation of ATPIF1 during the development of hypertrophy serves as a signaling mechanism to coordinate cell growth with its metabolic requirement. These observations collectively challenge the dogma that hypertrophied hearts switch to glucose utilization as a simple compensatory response to downregulation of FAO. Instead, the metabolic switch appears to be programmed to promote cell growth at the expense of energy supply, which ultimately becomes maladaptive as the heart transitions from hypertrophy to failure (38).

We find that upregulation of ATPIF1 triggers the metabolic switch via activation of HIF1α signaling in pathological cardiac hypertrophy. Cardiac-specific deletion of HIF1α in mice has been shown to prevent the metabolic switch and cardiac hypertrophy (15). HIF1α is a powerful transcriptional stimulator of glycolysis, and its activation has been reported in pathological cardiac hypertrophy, but the upstream trigger of HIF1α activation is less clear. It has been suggested that cardiomyocyte hypertrophy could reduce capillary density and create a hypoxic environment in the heart (39–41). However, activation of HIF1α by such a mechanism would likely occur in a late stage of hypertrophy. It would not explain the upregulation of HIF1α in cultured cardiomyocytes subjected to hypertrophic stimuli. Under normoxic conditions, a number of mitochondria-derived mediators have been found to promote HIF1α accumulation, including TCA cycle intermediates and mtROS (16, 42–45). Our study shows that mtROS are required for the metabolic switch in hypertrophied cardiomyocytes. Although mtROS-induced activation of hypoxia signaling is well documented, the underlying mechanisms appear to be complex and even controversial in the current literature (16, 46). In cancer cells expressing high levels of ATPIF1, activation of the NF-κB pathway by mtROS is implicated (47–49). Previous findings suggest that the HIF1α promoter is a direct target of NF-κB in vascular smooth muscle cells (50). Whether NF-κB is involved in HIF1α activation induced by ATPIF1 in hypertrophied heart warrants further investigation. Furthermore, other mechanisms triggered by increased mtROS or suppression of ATP synthase also may contribute to pathological remodeling of the heart.

In summary, the study demonstrates that ATPIF1 upregulation in the heart dictates the metabolic switch toward increased glycolysis. The findings identify a mechanism that upregulates glucose metabolism at the expense of oxidative ATP production during the pathological growth of cardiomyocytes. Our results propose a critical role of ATP synthase in toggling anabolic and catabolic metabolism during the pathological remodeling and add to an emerging concept that metabolic reprogramming is necessary for hypertrophy of the heart.

## Methods

### Animal model.

ATPIF1 was floxed by flanking of exon 3 with *loxP* sequences (Supplemental Figure 5A). The ATPIF1 F/F embryos with C57BL/6NTAC background were obtained from the European Mutant Mouse Archive. Frozen embryos were delivered to the Transgenic Resources Program of the University of Washington and were implanted into mice with C57BL/6N background to generate founders. To obtain cardiac-specific ATPIF1-KO mice, ATPIF1-floxed mice were then crossed with transgenic mice carrying α-MHC-Cre recombinase. ATPIF1 F/F mice were used as controls. Female and male mice have different responses to pressure overload or MI. TAC or MI surgery causes only a mild and variable phenotype in females. Thus, only male mice were used in this study. Adult male Sprague-Dawley rats (200–220 g) were obtained from Harlan Laboratories for the isolation of cardiomyocytes. Mice and rats were housed at a constant room temperature of 25°C under a 12-hour light/12-hour dark cycle with free access to food and drinking water.

### TAC surgery.

Male 8- to 12-week-old mice weighing 22–28 g underwent transverse aortic constriction (TAC) or sham surgery as previously described (51). Briefly, mice were anesthetized with 4% isoflurane and intubated with a 20-gauge cannula. Ventilation was initiated and continued under 2.5% isoflurane at 135 breaths per minute by a small-animal TOPO ventilator (Kent Scientific). The aortic arch was exposed via a left thoracotomy and by careful separation of the thymus. A constriction of the transverse aorta was generated by tying of a 6-0 Ethilon ligature against a 27-gauge blunt needle around the aorta between the brachiocephalic and left common carotid arteries. Promptly the needle was removed, lungs were inflated, and the chest and skin were closed by 5-0 polypropylene suture. The animal was removed from ventilation, and sustained-release buprenorphine (subcutaneous, 0.05 mg/kg) and 0.9% saline (i.p., 0.2 mL) were administered for analgesia and hydration, respectively. Sham-operated mice underwent all the same procedures as TAC mice excluding the constriction of the aorta. ATPIF1 F/F and cKO mice were randomly assigned to sham or TAC surgery. Combined mortality (acute and chronic) was less than 10%, and all mice surviving surgery were included in the analysis. Sample size was determined by power analysis based on our previous experiments.

### Induction of myocardial infarction.

Male 8- to 12-week-old mice weighing 22–28 g underwent permanent left anterior descending artery ligation or sham operation as previously described (52). Briefly, mice were anesthetized with 4% isoflurane and intubated with a 20-gauge cannula. Ventilation was initiated and continued under 2 % isoflurane at 130 breaths per minute by a small-animal TOPO ventilator (Kent Scientific). A small skin cut (~1.2 cm) was made over the left chest. After dissection and retraction of the pectoral major and minor muscle, the fourth intercostal space was exposed. A small hole was made at the fourth intercostal space, and a mosquito clamp was used to open the pleural membrane and pericardium. With the clamp slightly open, the heart was smoothly and gently popped out through the hole. The left coronary artery was located, sutured, and ligated at a site about 3 mm from its origin using a 7-0 Ethilon silk suture. After ligation, the heart was immediately placed back into the intrathoracic space, followed by manual evacuation of air and closure of muscle and the skin, by means of the previously placed purse-string suture. Mice that did not survive the first 24 hours after the surgery were excluded from analysis. Sham-operated animals underwent the same procedure without coronary artery ligation.

### Transthoracic echocardiography.

Mice were anesthetized and maintained with 0.8%–2% isoflurane in 95% oxygen at heart rates of 560–580 bpm. Transthoracic echocardiography was conducted at 4 weeks after MI surgery with a Vevo 3100 high-frequency, high-resolution digital imaging system (VisualSonics) equipped with an MS500 MicroScan Transducer. A parasternal short-axis view was used to obtain M-mode images for analysis of left ventricular (LV) wall thickness and internal dimensions at the end systole and diastole, respectively. LV fractional shortening, ejection fraction, and other cardiac functional parameters were calculated as described previously (22).

### Measurement of LV infarct size in MI model.

Mice were sacrificed at 72 hours after MI, and the heart was quickly excised and frozen in –20°C for 1 hour. After this period, the heart was semifrozen and sliced into six 1.0-mm-thick sections parallel with the short axis using a razor blade on a cold surface. The blood from sections was briefly rinsed with phosphate solution (PBS, pH 7.4), and then incubated with freshly prepared 1% triphenyltetrazolium chloride (TTC; MilliporeSigma, T8877) in PBS for 15 minutes at 37°C. The sections were placed in 10% neutral-buffered formalin for a maximum of 30 minutes and then imaged. For infarct size analysis, TTC-stained area and TTC-negative staining area (infarct myocardium) were measured using ImageJ (NIH). Myocardial infarct size was expressed as a percentage of the total LV area.

### Isolation and culture of adult mouse and rat cardiomyocytes.

Primary adult ventricular cardiomyocytes were isolated from Sprague-Dawley rats (200–224 g) or adult mice by a modified method (53). Briefly, after anesthesia with i.p. injection of Pentobarbital Sodium (Med-Pharmex, catalog NDC:54925-045-10) (0.15 mg/g of body weight), beating hearts were quickly removed from the chests and mounted onto a Langendorff perfusion apparatus. All perfusates were maintained at 37°C, and the hearts were first perfused with buffer containing 118 mM NaCl, 4.8 mM KCl, 1.2 mM KH_2_PO_4_, 1.2 mM MgSO_4_·7H_2_O, 11 mM glucose, 25 mM HEPES (pH 7.4), followed by the buffer supplemented with 0.2 mM Ca^2+^, 80 U/mL collagenase II (Worthington). After 20–30 minutes of digestion, the heart was taken down from the Langendorff perfusion system, and the aorta, atria, and connective tissue were removed. The digested ventricles were cut into small pieces (1–2 mm^2^) and combined with enzyme buffer to shake in 37°C water bath for several minutes. The cell suspension was centrifuged at low speed (50*g*) for 30 seconds, and rod-shaped adult cardiomyocytes were collected and plated at a density of 5 × 10^4^ per well in laminin-coated 6-well plates. The primary rat cardiomyocytes were cultured in M199 (MilliporeSigma, M2520) supplemented with 10 mM glutathione, 26.2 mM sodium bicarbonate, 0.02% BSA, and 1% penicillin/streptomycin (Sigma-Aldrich). The primary mouse cardiomyocytes were directly lysed by RIPA buffer (50 mM Tris, 150 mM NaCl, 1% Triton X-100, 0.1% SDS, 1 mM EDTA) for immunoblot assay.

### U-^13^C glucose tracing in vivo.

Seven days after MI, mice were fasted for 8 hours, and 1.5% isoflurane was used for anesthesia. U-^13^C glucose (2 mg/g; MilliporeSigma, catalog 389374) was injected i.p. Hearts were harvested 30 minutes after injection, and the ventricular tissue was rapidly flushed with cold 0.9% NaCl solution and freeze-clamped with Wollenberger tongs precooled in liquid nitrogen. Heart tissue (30 mg) was homogenized in 2 mL extraction buffer comprising methanol, chloroform, and H_2_O (1:3:1). Methylsuccinate was added as internal standard. The extract was stirred for 1 hour at 4°C and then centrifuged at 13,000*g*, 4°C, for 15 minutes. In total, 300 µL of supernatant was dried at 30°C on SpeedVac (Thermo Fisher Scientific) followed by 90 minutes of incubation with 30 μL of 20 mg/mL methoxyamine-HCl (MilliporeSigma, catalog 226904) in pyridine at 37°C. For unlabeled metabolites, 70 μL of MSTFA (Thermo Scientific, catalog TS48915) was then added and heated at 37°C for 30 minutes. For ^13^C-labeled metabolites, 70 μL of MTBSTFA (MilliporeSigma, catalog 394882) was then added and heated at 70°C for 30 minutes. Samples were analyzed using an Agilent Biosciences 7890 GC instrument/5977 mass-selective detector (MSD) with HP-5MS UI GC column. Gas chromatography–mass spectrometry (GC/MS) conditions were used according to the Fiehn library instructions from Agilent Biosciences. Retention times of individual metabolites were annotated according to known standards. For unlabeled metabolites, peak intensities were normalized to methylsuccinate intensity and tissue weight. For ^13^C-labeled metabolites, IsoCor software was used to correct the fractional labeling for natural isotopic abundance and to quantify isotopologue distribution of labeled metabolites.

### Isolated heart perfusion experiments and NMR spectroscopy.

Isolated mouse hearts were perfused in Langendorff mode at 37°C and a constant pressure as previously described (54). The perfusate contained 118 mM NaCl, 25 mM NaHCO_3_, 5.3 mM KCl, 2 mM CaCl_2_, 1.2 mM MgSO_4_, 0.5 mM EDTA, 5.5 mM glucose, 0.4 mM mixed long-chain fatty acids (bound to 1.2% albumin), 1.2 mM lactate, and 50 μU/mL insulin, equilibrated with 95% O_2_ and 5% CO_2_ (pH 7.4). Changes in cardiac high-energy phosphate content were monitored by ^31^P NMR spectroscopy simultaneously with a continuous recording of LV function via the PowerLab data acquisition system (AD Instruments). The ATP level was calculated by the average of the peak areas from γ-ATP and β-ATP.

### Construction of plasmid and adenoviral vectors.

pBHGloxΔE1,3Cre (Microbix), including the ΔE1 adenoviral genome, was cotransfected with pDC shuttle vector containing the gene of interest into HEK293 cells using Lipofectamine 2000 (Invitrogen). The ATPIF1 promoter sequence (928 bp) or truncated fragments fused with a dual-luciferase reporter sequence (pNLCoI1 66/2726 bp; Promega, catalog N146A) were inserted into XbaI and SalI sites in the plasmid pDC316 with the deletion of the mCMV promoter. Adenovirus harboring the ATPIF1 promoter sequence with luciferase reporter was used to test the mechanism of ATPIF1 transcriptional activity. Adenovirus harboring β-galactosidase (Ad-LacZ) was used as a control for overexpressing cells. Adenovirus harboring scrambled sequence was used as a control for knockdown cells. Full-length rat ATPIF1 was inserted into EcoRI and SalI sites in the plasmid pDC316. Adenoviruses harboring shRNA for rat ATPIF1 were generated using the following hairpin-forming oligonucleotides: ATPIF1: 5′-GTGTGCTACTAACAGATAATATTCAAGAGATATTATCTGTTAGTAGCACAC-3′.

Oligonucleotides flanked with HindIII and ApaI were synthesized, annealed, and inserted into vector pDC311. The loop sequences are underlined.

### Luciferase activity assay.

Primary adult rat ventricular cardiomyocytes (5 × 10^4^ per well) were cultured in a 6-well plate and treated with luciferase adenovirus or with 20 μM T-5224 (Cayman Chemical, catalog 22904) the same day. After 24 hours of culture, phenylephrine (PE) at a final concentration of 10 μM was added and cultured for another 24 hours until assay. The luciferase activity was determined using the dual-luciferase reporter assay system (Promega, catalog E1910). The ratio of firefly to Renilla luciferase activity was calculated.

### Cell culture, transfection, and nuclear isolation.

HEK293 cells were purchased from American Type Culture Collection (ATCC) and grown in DMEM high-glucose medium (Gibco) supplemented with 10% FBS (MilliporeSigma) at 37°C and 5% CO_2_. Transfection of the plasmid DNA was performed with Lipofectamine 2000 (Invitrogen). Nuclei from HEK293 cells were isolated using a commercial kit (Abcam, catalog ab113474).

### Metabolite assessment.

Left ventricular tissues were collected by snap-freezing and homogenized in PBS at 4°C. The homogenized lysates were centrifuged at 12,000*g* at 4°C to collect supernatant. The primary cardiomyocytes were cultured in M199 with indicated treatments, and culture medium was collected. Lactate concentration was determined using an L-Lactate Assay Kit (Trinity Biotech, 736-10). Intracellular NAD(H) level and glutathione were determined by an EnzyChrom NAD^+^/NADH Assay Kit (Bioassay Systems, E2ND-100) and a GSH/GSSG Ratio Detection Assay Kit (Abcam, ab239709), respectively, according to the manufacturers’ protocols. To assay glycogen level, hearts were harvested 7 days after MI or sham surgery. Mice were fasted for 8 hours before tissue harvesting. Ventricular tissues were quickly excised and briefly washed with cold 0.9% NaCl before snap-freezing. Glycogen was separated from exogenous glucose in 10 mg cardiac tissue by an alkaline extraction procedure and measured using a commercially available kit (BioVision, catalog k960-400).

### Glycolytic enzyme activity assay.

The primary cardiomyocytes were cultured in M199 with indicated treatments, and the cells were harvested in 0.1 M KPO_4_ buffer (pH 7.5) and immediately freeze-thawed using liquid nitrogen 3 times to release intracellular proteins. Subsequently, lysates were centrifuged for 10 minutes at 12,000*g* at 4°C to collect supernatant. The reaction mixture contained 1.9 mL of the assay buffer (0.15 M CAPS, pH 10.0), 0.5 mL of 6 mM NAD^+^ solution, 0.5 mL of 0.15 M l-lactate solution, and 0.1 mL of supernatant plus water in a total volume of 3 mL, and the absorption at 340 nm was measured for 5 minutes. The ratio of supernatant to water was decided by the absorption rate at A_340_ (0.1 or 0.2 per minute). The LDH activity was determined via measurement of the conversion of NAD^+^ to NADH. The results were normalized to the protein content. The protein concentrations were quantified by bicinchoninic acid (BCA) assay. The GAPDH activity was determined by a KDalert GAPDH Assay Kit (Life Technologies, AM1639) according to the manufacturer’s protocol.

### ATP synthesis rate.

The ATP synthesis rate was determined in digitonin-permeabilized cardiomyocytes (55). ATP content at multiple time points was determined using an ATP Bioluminescence Assay Kit CLS II (Roche, 11699695001) to assess the linear rate of ATP synthesis (7).

### Cell imaging.

To monitor the generation of mitochondrial ROS (mtROS) in real time, cardiomyocytes were stained with 5 μM MitoSOX (Molecular Probes) in M199 medium for 15 minutes. To assay ATPase activity in real time, cardiomyocytes were stained with 5 μM MgGreen (Invitrogen) in M199 medium for 30 minutes in a CO_2_ incubator. The live cardiomyocytes were subsequently cultured in cardiomyocyte isolation buffer (118 mM NaCl, 4.8 mM KCl, 1.2 mM KH_2_PO_4_, 1.2 mM MgSO_4_·7H_2_O, 11 mM glucose, and 25 mM HEPES, pH 7.4). To observe EGFP-HIF1α (http://www.addgene.org/87204/) localization induced by ATPIF1, transfected cells were fixed with 4% paraformaldehyde in PBS for 15 minutes and then permeabilized with 0.1% Triton X-100 in PBS for 30 minutes at room temperature. Cells were stained with 200 nM DAPI for 30 minutes, and fluorescent signals of the cells were examined using a Leica TCS SP8 confocal microscope.

### ROS detection in H9C2 cells.

H9C2 cells were purchased from ATCC. To measure mitochondrial and cytosol ROS production, the treated H9C2 cells were incubated with 5 μM MitoSOX Red (Invitrogen, M36008) or 2.5 μM CM-H2DCFDA (Invitrogen, C6827) at 37°C for 30 minutes in high-glucose DMEM (Gibco, 11995) without FBS, followed by washing twice with PBS. The cells were analyzed on a spectral flow cytometer (Aurora, Cytek Biosciences), and the results were analyzed using FlowJo software (BD Life Sciences).

### Assay of extracellular acidification rates.

Extracellular acidification rate (ECAR) measurement was performed using a Seahorse XF^e^24 Extracellular Flux Analyzer (Agilent Biosciences). Adult rat cardiomyocytes were plated in M199 medium at 10,000 cells per well and treated with adenovirus to overexpress (48 hours) or knock down (72 hours) ATPIF1. To induce cardiomyocyte hypertrophy, PE at a final concentration of 10 μM was added into M199 medium for 24 hours before the assay. Before assay, cells were washed twice with XF assay medium (supplemented with 2 mM l-glutamine, pH 7.4). Then, cells were cultured in a non-CO_2_ incubator for 30 minutes before assay. For ECAR measurements, glucose (10 mM), rotenone and antimycin A (1 μM), and 2-deoxyglucose (50 mM) were sequentially injected, so that basal glycolysis, maximum glycolysis, and non-glycolysis ECAR were calculated.

### Isolation of mitochondria from mouse heart and adult rat cardiomyocytes.

Hearts were excised and placed into ice-cold MSE buffer (70 mM sucrose, 210 mM mannitol, 5 mM MOPS, 2 mM taurine, 1.6 mM carnitine hydrochloride, and 1 mM EDTA, pH 7.4) to rinse off blood. Hearts were minced at 4°C and rinsed in MSE buffer. The heart tissue was resuspended with MSE buffer supplemented with 0.1 mg/mL trypsin and incubated on ice for 10 minutes. Then the same volume of MSE buffer with 0.2% of fatty acid–free BSA, 0.5 mg/mL trypsin inhibitor was added to neutralize the trypsin. All subsequent steps were performed on ice or at 4°C. The heart tissue was resuspended in 4 mL MSE buffer with 0.2% BSA and homogenized with a glass Dounce homogenizer (6 strokes at 1200 rpm). The homogenate was centrifuged at 600*g* for 5 minutes to remove tissue debris and nuclei. The supernatant was transferred into dolphin-shaped tubes and centrifuged at 8000*g* for 10 minutes. The mitochondrial pellet was resuspended in MSE buffer and recentrifuged at 8000*g* for 5 minutes. Cardiomyocytes were cultured for 48 hours and collected in ice-cold MSE buffer. The cells were centrifuged at 600*g* for 5 minutes at 4°C and resuspended with ice-cold MSE buffer. The mitochondria isolation was performed as described above.

### Measurement of mitochondrial respiration.

Isolated cardiac mitochondrial respiration was determined using a Seahorse XF^e^24 analyzer. Mitochondrial pellet was loaded in 50 μL of MAS buffer (70 mM sucrose, 220 mM mannitol, 10 mM KH_2_PO_4_, 5 mM MgCl_2_, 2 mM HEPES, 1 mM EGTA, and 0.2% fatty acid–free BSA, pH 7.4) supplemented with 10 mM pyruvate and 2 mM malate. For the state 3 respiration assay, mitochondria were isolated from mouse heart and cultured cardiomyocytes. Isolated mitochondria (1.5 μg/well) in MAS buffer supplemented with 10 mM pyruvate and 2 mM malate were seeded into a Seahorse XF^e^24 plate by centrifugation at 2000*g* for 20 minutes at 4°C. Then another 450 μL of MAS buffer supplemented with 10 mM pyruvate and 2 mM malate was added in a total volume of 500 μL and incubated at 37°C for 10 minutes before assay. A final concentration of 4 mM ADP, 2.5 μM oligomycin A, 4 μM FCCP, and 4 μM antimycin A was injected sequentially. For the electron flow assay, mitochondria were isolated from cultured cardiomyocytes. MAS buffer was supplemented with 10 mM pyruvate, 2 mM malate, and 4 mM FCCP, and the mitochondria were loaded in the same way as described above. Two micromolar rotenone, 10 μM succinate, 4 μM antimycin A, and 10 mM/0.5 mM ascorbate/TMPD were injected sequentially.

### Western blotting.

Heart homogenates or cell lysates were made in RIPA buffer (50 mM Tris, 150 mM NaCl, 1% Triton X-100, 0.1% SDS, 1 mM EDTA) with a protease inhibitor cocktail. The protein concentrations were determined using a Pierce BCA Protein Assay Kit. Protein samples (10–20 μg) were loaded per lane on an SDS-polyacrylamide gel. Antibodies were obtained from the following sources: ATPIF1 (catalog 8528), LDHA (catalog 2012), PKM2 (catalog 4053), VDAC (catalog 4661), vinculin (catalog 13901), and histone 3 (catalog 4499) were from Cell Signaling Technology; OXPHOS cocktail (catalog ab110413), cytochrome *c* core 1 (catalog ab110252), COX-4 (catalog ab14744), and HIF1α (catalog ab1) were from Abcam; NDUFS4 (catalog MA5-19432) and COX-1 (catalog PA5-26688) were from Thermo Fisher Scientific; cytochrome *b* (catalog sc-11436), ATP5A (catalog sc-136178), and ATP5B (catalog sc-33618) were from Santa Cruz Biotechnology; GFP (catalog 11814460001), β-actin (catalog A2103), and α-tubulin (catalog T6199) were from MilliporeSigma.

### Total RNA isolation and quantitative real-time PCR.

Total RNA was isolated from cardiomyocytes using Trizol reagent (Invitrogen) or using the RNeasy Fibrous Tissue Mini Kit (Qiagen) according to the manufacturer’s instructions. The cDNA was synthesized using iScript Reverse Transcription Supermix (Bio-Rad). cDNA levels were quantified by the Applied Biosystems 7900HT Fast Real-Time PCR System using SYBR Green (Bio-Rad). mRNA expression was normalized with 18S rRNA expression, and the quantification evaluation was calculated by ΔΔCt values. The primers used for the measurement were as follows: mouse ATPIF1 forward: GGTGTCTGGGGTATGAAGGTC; mouse ATPIF1 reverse: CCTTTTCTCGTTTTCCGAAGGC; mouse BNP forward: GCCAGTCTCCAGAGCAATTCA; mouse BNP reverse: GGGCCATTTCCTCCGACT; mouse GAPDH forward: CTTTGTCAAGCTCATTTCCTGG; mouse GAPDH reverse: TCTTGCTCAGTGTCCTTGC; mouse Glut1 forward: GATTGGTTCCTTCTCTGTCGG; mouse Glut1 reverse: CCCAGGATCAGCATCTCAAAG; mouse HK2 forward: TCAAAGAGAACAAGGGCGAG; mouse HK2 reverse: AGGAAGCGGACATCACAATC; mouse LDHA forward: GCTCCCCAGAACAAGATTACAG; mouse LDHA reverse: TCGCCCTTGAGTTTGTCTTC; mouse PKM2 forward: CCATTCTCTACCGTCCTGTTG; mouse PKM2 reverse: TCCATGTAAGCGTTGTCCAG; rat ATPIF1 forward: TGTCTGGGGTATGAGGGTCCT; rat ATPIF1 reverse: TTCAGCCTTCTCTCGTTTCCCG; rat BNP forward: CTTTTCCTTAATCTGTCGCCG; rat BNP reverse: GTCTCTGAGCCATTTCCTCTG; rat GAPDH forward: CCATCAACGACCCCTTCATT; rat GAPDH reverse: GACCAGCTTCCCATTCTCAG; rat Glut1 forward: TGATTGGTTCCTTCTCTGTGG; rat Glut1 reverse: CCCAGGATCAGCATCTCAAAG; rat LDHA forward: ACTGCTCATCGTCTCAAACC; rat LDHA reverse: CTTTCTCCCATCAGGTAACGG; rat PKM2 forward: GTGGAGATGCTGAAGGAGATG; rat PKM2 reverse: AGGTCGGTAGAGAATGGGATC; rat c-fos forward: ACGGAGAATCCGAAGGGAAAGGAA; rat c-fos reverse: TCTGCAACGCAGACTTCTCGTCTT; universal 18S rRNA forward: GTAACCCGTTGAACCCCATT; universal 18S rRNA reverse: CCATCCAATCGGTAGTAGCG.

### Chemical cross-linking, liquid chromatography–mass spectrometry analysis, and structural analysis.

Chemical cross-linking of heart tissue was performed using a previously published method (4). Heart tissue from 5 TAC and 5 sham-operated mice was harvested and minced into 1-mm^3^ pieces. Minced mouse heart tissue was rinsed in MSE buffer without taurine and centrifuged at 1500*g* for 3 minutes at 4°C, and supernatant was removed. Tissue was suspended in 0.25 mL of 170 mM Na_2_HPO_4_ (pH 8.0). The isobaric quantitative PIR cross-linker GG-BDP-NHP (reporter light or reporter heavy isotope forms) was added to a final concentration of 10 mM from a 233 mM stock solution in DMSO. The sample was mixed on an Eppendorf ThermoMixer at 800 rpm for 30 minutes at room temperature. The tissue was then centrifuged at 1500*g* for 3 minutes, and the supernatant was removed. Mitochondria were isolated from the cross-linked heart tissue as described above.

Isolated mitochondria were lysed in 0.1 mL 8 M urea, 0.1 M NH_4_HCO_3_. Sample viscosity was reduced by sonication (5 times, 5-second pulses at amplitude 40–60) using a GE-130 ultrasonic processor, followed by reduction and alkylation of cysteine residues by incubation with 5 mM tris(2-carboxyethyl)phosphine (TCEP; Thermo Fisher Scientific) for 30 minutes, followed by a 45-minute incubation with 10 mM iodoacetamide (Thermo Fisher Scientific). To reduce the urea concentration to less than 1 M, the samples were diluted by a factor of 10 with fresh 0.1 M NH_4_HCO_3_. The protein concentration was measured using the Pierce Coomassie protein assay (Thermo Fisher Scientific). Protein originating from TAC and sham samples were mixed at a 1:1 ratio resulting in 5 paired TAC/sham samples. Protein samples were digested with trypsin at a 1:100 (trypsin/protein) concentration at 37°C for 16 hours with constant mixing.

Digested peptides were desalted with a C18 Sep-Pak cartridge (Waters), and the eluted peptides were dried to completion before being resuspended in SCX buffer A (7 mM KH_2_PO_4_, pH 2.6, 30% [vol/vol] acetonitrile [ACN]). Resuspended peptides were injected into a Phenomenex Luna SCX column and were fractionated using a 97.5-minute gradient of buffer B (7 mM KH_2_PO_4_ [pH 2.6], 30% [vol/vol] ACN, 350 mM KCl) as follows: 0% buffer B at 0 minute, 5% B at 7.5 minutes, 60% B at 47.5 minutes, 100% B at 67.5 minutes, 100% B at 77.5 minutes, 0% B at 77.51 minutes, and 0% B at 97.5 minutes. Fractions were taken every 5 minutes starting at 17.5 minutes and were pooled into 6 pools as follows: fractions 1–5, fractions 6–7, fraction 8, fraction 9, fraction 10, and fractions 11–14. Fraction pools were dried to a final volume of approximately 2 mL in a vacuum centrifuge, and the pH was adjusted to 8.0 with 1.5 M NaOH. Pooled cross-linked peptides were enriched by the addition of 200 μL of monomeric avidin bead slurry (UltraLink, Pierce) and were incubated at room temperature on an orbital shaker at maximum speed. Cross-linked peptides were washed with 100 mM ammonium bicarbonate (pH 8.0) and eluted with 70% (vol/vol) ACN/1% formic acid before being dried to completion in a vacuum centrifuge and stored at −80°C until analyzed by liquid chromatography–tandem mass spectrometry (LC-MS/MS).

Samples containing cross-linked peptides were dissolved in 30 μL of 0.1% formic acid in H_2_O. LC-MS/MS analysis was performed using an Easy-nLC (Thermo Fisher Scientific) coupled to a Q-Exactive Plus mass spectrometer (Thermo Fisher Scientific). In total 3 µL of each sample was injected by the autosampler of the Easy-nLC and loaded onto a fused silica trap column (3 cm × 100 μm inner diameter) packed with ReproSil C8 (particles 5 μm diameter, 120 Å pore size) (Dr. Maisch GmbH) with a flow rate of 2 μL/min of solvent A (0.1% formic acid in H_2_O) for 10 minutes. Peptides were fractionated over a fused silica analytical column (60 cm × 75 μm inner diameter) packed with ReproSil C8 (particles 5 μm diameter, 120 Å pore size) by application of a 2-hour linear gradient ramping the mobile phase composition from 90% solvent A, 10% solvent B (0.1% formic acid in acetonitrile), to 60% solvent A, 40% solvent B, at a flow rate of 300 nL/min. Eluting peptide ions were ionized by nano-electrospray ionization by application of a positive 2 kV potential to a laser-pulled spray tip at the end of the analytical column. The Q-Exactive Plus mass spectrometer operated using a data-dependent analysis method consisting of an MS1 scan at 70,000 resolving power at *m*/*z* 200 followed by MS2 scans at 70,000 resolving power on the 5 most abundant ions in the MS1. Ions with charge states 4–7 were selected for MS2, isolated with a 3 *m*/*z* window, and fragmented with a normalized collision energy of 20.

Raw files were converted to mzXML format and searched using Comet (56) against the MitoCarta2 (57) protein sequence database containing both forward and reverse protein sequences (2084 total). The search results were processed with XLinkProphet (58) and filtered to an estimated false discovery rate of less than 1% at the nonredundant cross-linked peptide pair level. Quantification of cross-linked peptide pairs was performed by comparison of the relative intensities of isotopically light and heavy fragment ions in the MS2 spectra of identified cross-linked peptide pairs (19). Cross-links were analyzed in context with the PDB structure 6j5k using XLinkDB (59).

### Statistics.

Statistical analyses were performed using Prism 8 software (GraphPad Software). Unpaired 2-tailed Student’s *t* test was used to test the differences between 2 groups. One- or two-way ANOVA was used to test the difference among multiple groups, followed by a post hoc examination of the *P* value between 2 groups. A 2-tailed *P* value of 0.05 was considered statistically significant.

### Study approval.

All studies, including animal and cell culture studies, were approved by the Institutional Animal Care and Use Committee at the University of Washington (protocol 4214-01).

## Author contributions

BZ and RT designed the experiments. BZ, XT, JEB, and RT wrote the manuscript. BZ performed most of the experiments. AC helped isolate mitochondria from mouse heart. AC, XT, and JDC performed cross-link experiments. AK developed computational tools to map mouse protein sequences against available structures from other species. SCK exercised mice to induce cardiac physiological hypertrophy. OV performed TAC surgery and MZ performed MI surgery to induce cardiac pathological hypertrophy. YL performed ROS assay in H9C2 cells and analyzed data. TSM perfused hearts and performed the NMR experiment. BZ, TSM, and JR performed the GC/MS experiment and analyzed data. PW isolated adult mouse cardiomyocytes. WW, JEB, and RT supervised the project.

## Supplementary Material

Supplemental data

## Version 1 05/16/2022

Electronic publication
