# Supplementary material for: Upregulation of mitochondrial ATPase inhibitory factor 1 (ATPIF1) mediates increased glycolysis in mouse hearts
Source: J Clin Invest. 2022 May 16;132(10):e155333. doi: 10.1172/JCI155333 (PMC9106352; doi:10.1172/JCI155333)
Supplement: Supplemental data [file jci-132-155333-s116.pdf]

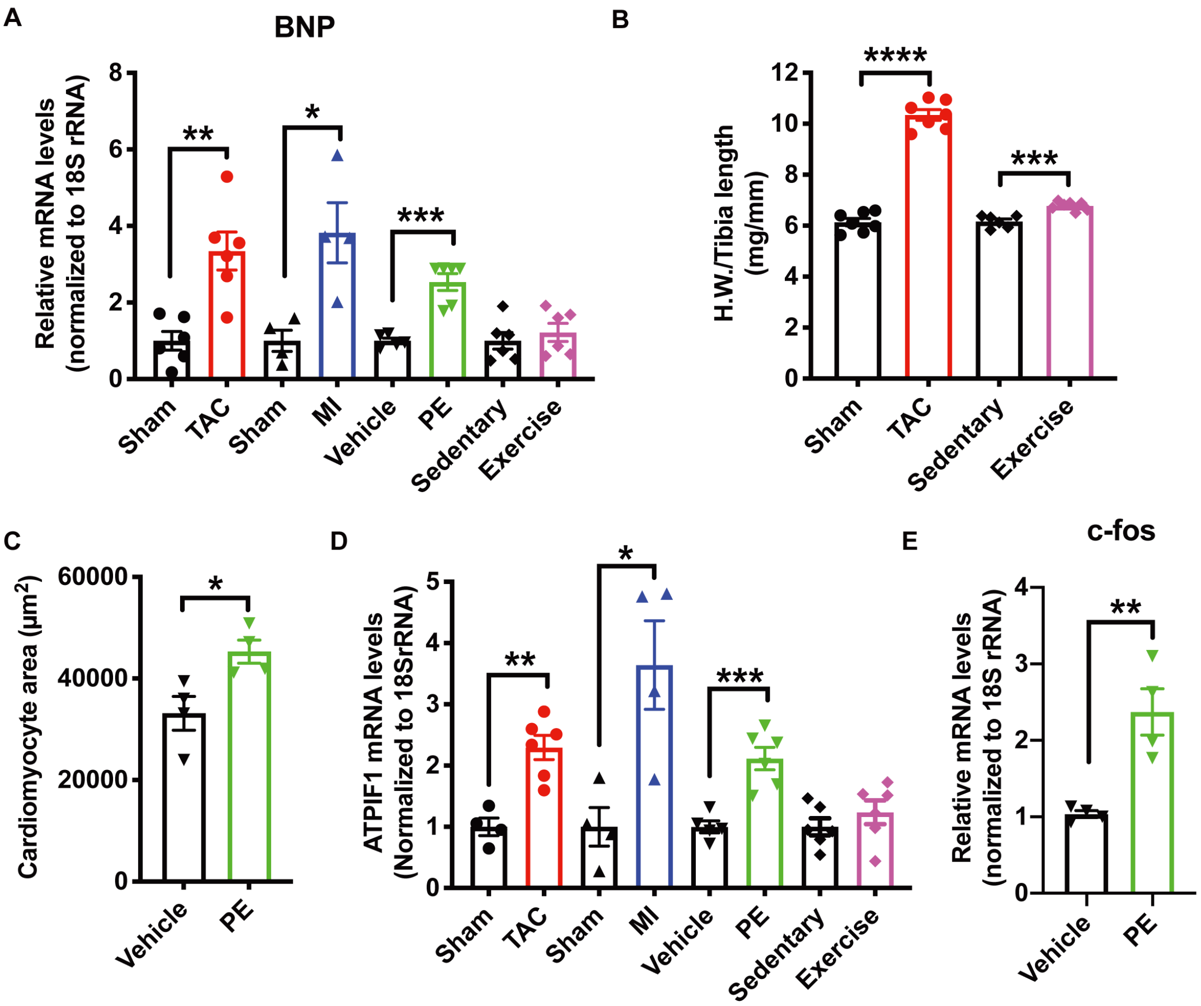

F

```
                                     ATGGGGGGTGTGGGTGTTTATTCGTTTG  -901
TTTCTGAAAACAGGATCTTGTTATGAAGCCCTGGCTGGCCCAGAATTCCCTTTGTCGAAT  -841
AGGCTAGATTAAGCTAGATTAAGCTCTTCCTGTGTGCCTCAGGAGTGCTGGGATTACAGG  -781
TTTGTACCACCAGTTCGCTTGATGTTCTTTTCAGTGTCCTTGTAGCCCGTCACCAAGTTA  -721
TTAGGTCCATTCACCGACACATTGATACAGTTCAAAATTACTAATTCACTACCTACTAAG  -661
GCTCTGATGGCTTCACAAAGTAAACTGTGAGAAAGAACTCAGAAGCAAGGATAAAAAAAA  -601
ATTGCTGTCAGGATGACTAGGAAGGTCAAAGAAGGGTTTCTCTGAGAAAGTGACATTTAA  -541
GGTGAGAGAAGGCACTGCAGTGGCAATATAACCAAAGAACAAGAACACCTCTTAAGAGAT  -481
AAACACGATTTCATATGTATAAACAGAGGTTTGAGGTCGATATCCAGGCTGTCGGCATCC  -421
TAGGCATGGCTACCACAAACTTTGTGTTTCCCCAATCTATTCATGTCCATAGGGCTTGCC  -361
ATCGTGTACATTTTTAATAAAATCGACCTTTGGACGGAAATTGTGCAGTGGGAGTGAACG  -301
AAATGCCTCATTCTAAAGAGACCAAAAGCCAAGATTCATCTTTTGACTTCGCTCTGTAG  -241
ACTCCTGGGTCATGATGCGTTAGAGCCTTGTTTATTAACTGTTAACTAAGGGATAAATCC  -181
GCCTCCCTCTCAACAAACGCTGCAGATATCATAAGCCCCGCCCCCTCCCTCCTGATTCGC  -121
GGTTTCCGTGCTCGTCCCACCTCCCCGGCGTCTAGATTGGCAGTTCAGCCGCCCTCGCAG  -61
TTAGCGCGCTAGGAGAGAAGCAGCCACGCCCGCAACGCGAGCTGAGCAACGCCGAAGACA  0
ATGGCAGGCTCGGCGTTGGCAGTTCGGGCTCGGTTCGGTGTCTGGGGTATGAAGGTCCTG  60
```

Supplementary 1. Assessment of cardiac hypertrophy and ATPIF1 gene expression.

(A) Brain natriuretic peptide (BNP) mRNA levels (n=4-6); (B) Heart weight (HW) to tibia length ratio (n=7); (C) Cross sectional area of primary adult rat cardiomyocytes treated with 10 μM phenylephrine (PE) for 48 hrs (n=4); and (D) ATPIF1 mRNA expression were assessed (n=4-6) in each group. (E) c-fos mRNA expression was assayed in primary adult rat cardiomyocytes (n=4). (F) The upstream nucleotide sequence (-928/-1bp) of mouse ATPIF1 gene. The candidate AP-1 complex binding sites are denoted in red and underlined. 18S rRNA was served as mRNA internal control. Data are means ± SEM of the values. P values were determined using unpaired Student's t-test comparing the treatment group with its respective controls; *p < 0.05; **p<0.01; ***p<0.001; ****p<0.0001.

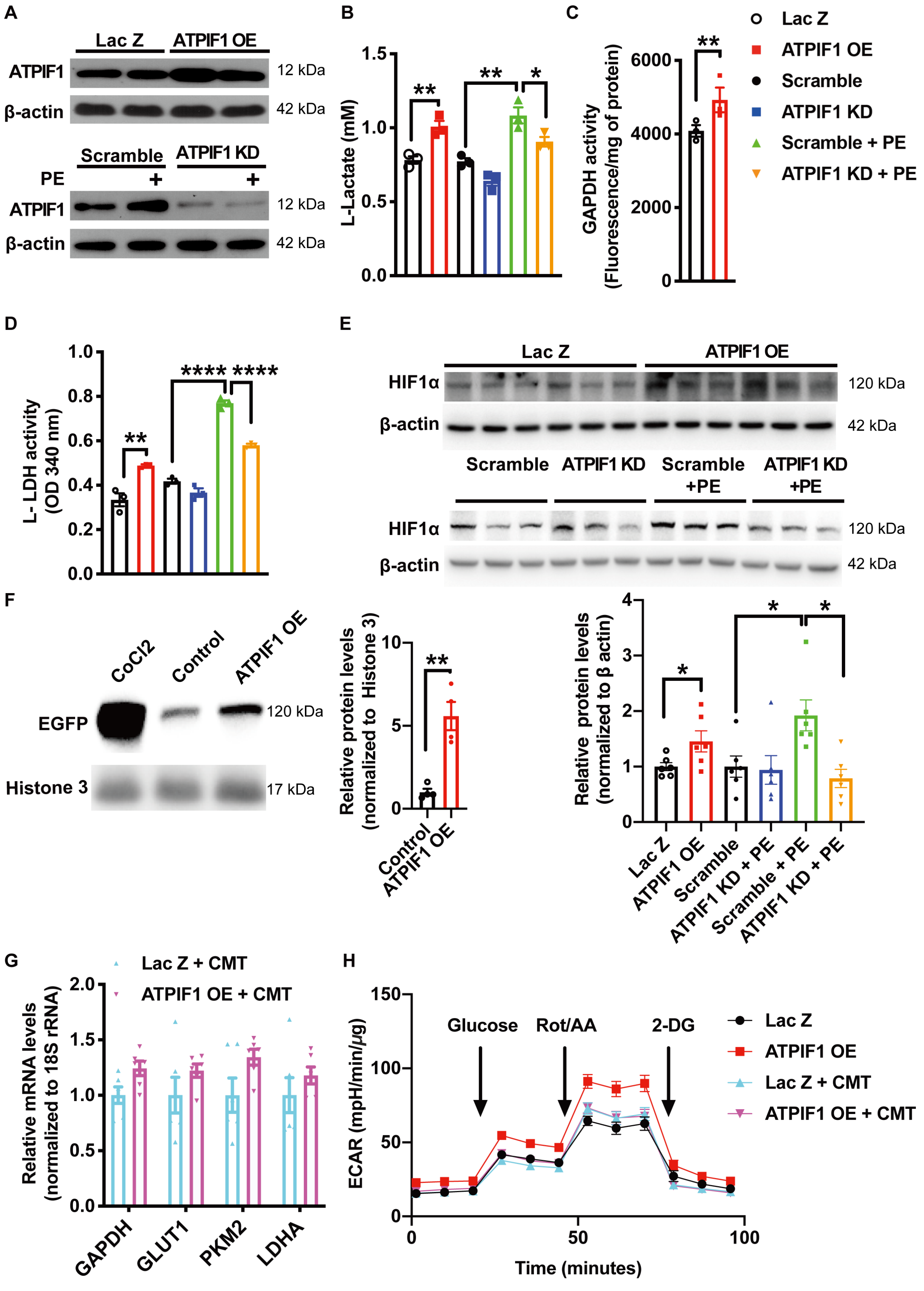
A
Lac Z
ATPIF1 OE
ATPIF1
β-actin
12 kDa
42 kDa
Scramble
ATPIF1 KD
PE
+
+
ATPIF1
β-actin
12 kDa
42 kDa
B
L-Lactate (mM)
**
**
*
C
GAPDH activity
(Fluorescence/mg of protein)
**
Lac Z
ATPIF1 OE
Scramble
ATPIF1 KD
Scramble + PE
ATPIF1 KD + PE
D
L- LDH activity
(OD 340 nm)
**
**** ****
E
Lac Z
ATPIF1 OE
HIF1α
β-actin
120 kDa
42 kDa
Scramble
ATPIF1 KD
Scramble +PE
ATPIF1 KD +PE
HIF1α
β-actin
120 kDa
42 kDa
F
CoCl2
Control
ATPIF1 OE
EGFP
120 kDa
Histone 3
17 kDa
Relative protein levels
(normalized to Histone 3)
**
Control
ATPIF1 OE
Relative protein levels
(normalized to β actin)
*
*
*
Lac Z
ATPIF1 OE
Scramble
ATPIF1 KD + PE
Scramble + PE
ATPIF1 KD + PE
G
Lac Z + CMT
ATPIF1 OE + CMT
Relative mRNA levels
(normalized to 18S rRNA)
GAPDH
GLUT1
PKM2
LDHA
H
ECAR (mpH/min/μg)
Glucose
Rot/AA
2-DG
Lac Z
ATPIF1 OE
Lac Z + CMT
ATPIF1 OE + CMT
Time (minutes)

Supplementary 2. Upregulation of ATPIF1 enhanced glycolytic activity in cardiomyocytes

(A) ATPIF1 protein level assay in cardiomyocytes transduced by ATPIF1 adenovirus or ATPIF1 adenoviral shRNA with or without phenylephrine (PE, 10 μM) after 48 or 72 hrs culture. (B) Measurement of lactate level from the culture medium of cardiomyocytes (n=3). (C) GAPDH activity assay in cardiomyocytes after 48 hrs of culture (n=3). (D and E) LDHA activity assay (D) and HIF1α immunoblot analysis (E) of cardiomyocytes in the overexpression or knockdown of ATPIF1 with or without PE after 48 or 72 hrs culture (Lac Z, control for overexpressing cells; Scramble, control for knockdown cells; n=3-6). (F) Representative immunoblot and group average of EGFP level from isolated nuclei in HEK293 expressing HIF1α-EGFP and ATPIF1 or empty vector controls (n=4). (G) Glycolytic gene expression in cardiomyocytes overexpressing ATPIF1 after the inhibition of HIF1α activity by CMT (n=6). (H) ECAR tracing of cardiomyocytes overexpressing ATPIF1 or LacZ with or without the addition of HIF1α inhibitor (chetomin, CTM). Data represent the average from 4 independent experiments, each experiment has 4-5 replicate wells. All values were normalized to 18S rRNA expression and shown as the change over LacZ + CMT. Data are means ± SEM of the values. P values were determined using unpaired Student's t-test for Lac Z vs. ATPIF1 OE comparison or two-way ANOVA followed by Tukey's multiple comparison test for comparison of Scramble vs. ATPIF1 KD vs. Scramble+PE vs. ATPIF1 KD+PE; *$p < 0.05$; **$p<0.01$; ****$p<0.0001$).

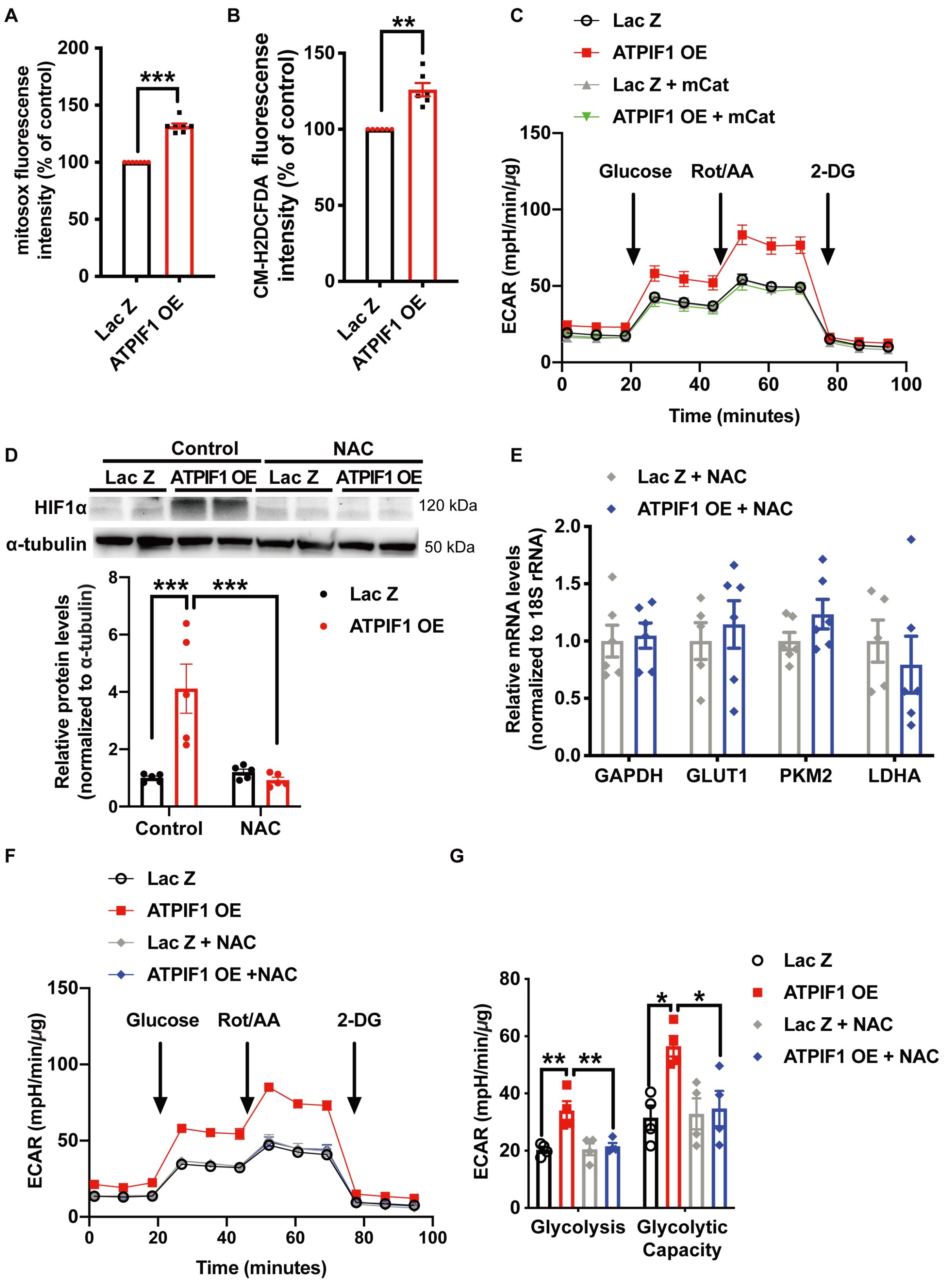
A
mitosox fluorescense intensity (% of control)
Lac Z
ATPIF1 OE
***
B
CM-H2DCFDA fluorescense intensity (% of control)
**
C
Lac Z
ATPIF1 OE
Lac Z + mCat
ATPIF1 OE + mCat
Glucose
Rot/AA
2-DG
ECAR (mpH/min/μg)
Time (minutes)
D
Control
NAC
Lac Z
ATPIF1 OE
HIF1α
α-tubulin
120 kDa
50 kDa
Relative protein levels (normalized to α-tubulin)
E
Lac Z + NAC
ATPIF1 OE + NAC
Relative mRNA levels (normalized to 18S rRNA)
GAPDH
GLUT1
PKM2
LDHA
F
Lac Z + NAC
ATPIF1 OE +NAC
G
Glycolysis
Glycolytic Capacity

Supplementary 3. ROS accumulation contributed to increased glycolysis by ATPIF1

(A and B) Mitochondrial superoxide production assessed with mitoSOX (A, n=5) and reactive oxygen species (ROS) assessed with CM-H2DCFDA (B, n=3) in H9C2 cells. (C) ECAR tracing of cardiomyocytes with co-expression of ATPIF1 and mitochondrial catalase (mCat). Data represent the average from 5 independent experiments, each experiment has 4-5 replicate wells. (D, E, F and G) Representative immunoblot of HIF1α protein (D, n=5), real-time PCR analysis of glycolytic gene expression (E, n=6) and ECAR tracing and tabulated data (F and G, n=4) in cardiomyocytes with or without 0.2 mM NAC (N-acetylcysteine) treatment for 24 hrs. Data represent the average from 4 independent experiments, each experiment has 4-5 replicate wells (F and G). Gene expression values were normalized to 18S rRNA expression and shown as relative change over the average of the control sets (E). Data are means ± SEM of the values. P values were determined using unpaired Student's t-test (A, B, E) or two-way ANOVA followed by Tukey's multiple comparison test (D); *$p < 0.05$; **$p<0.01$; ****$p<0.0001$.

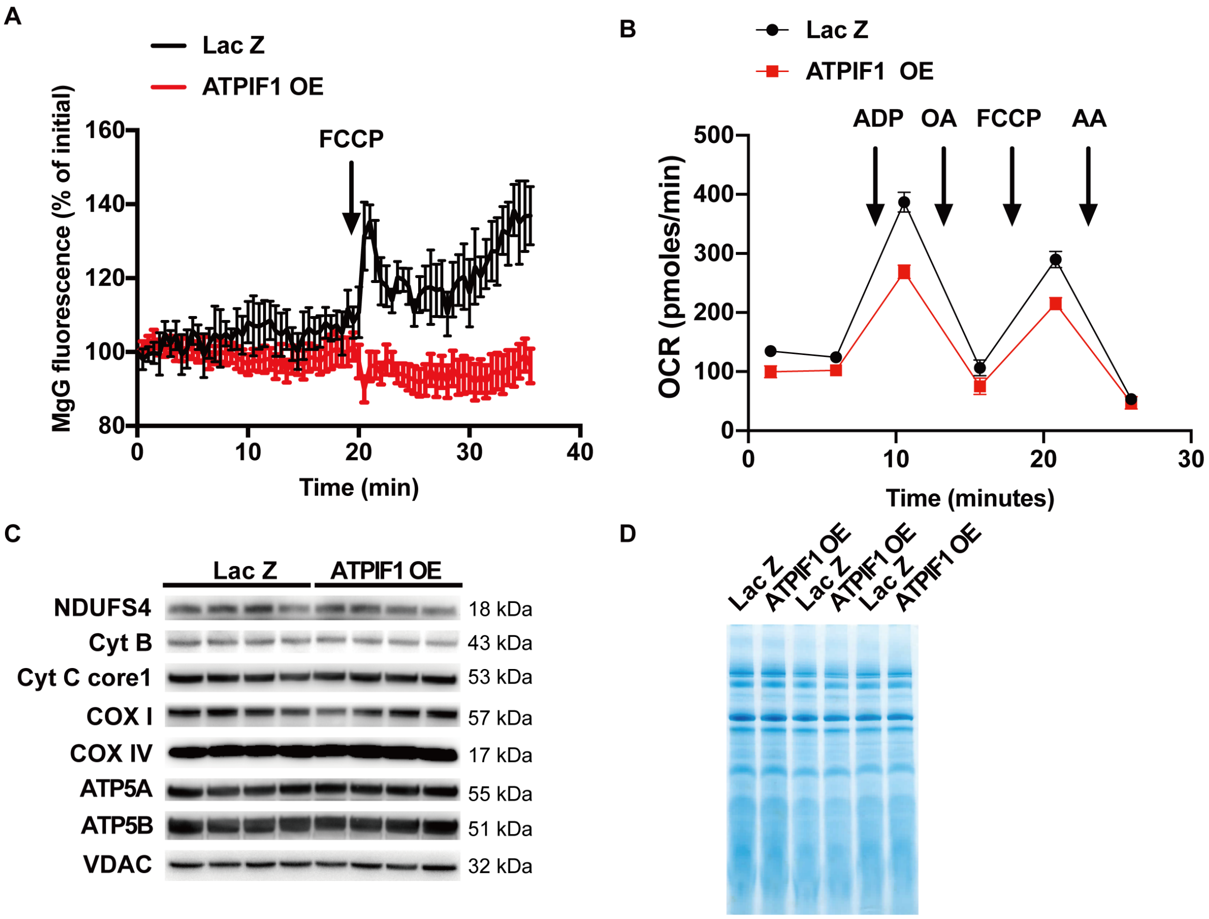

Supplementary 4. ATPIF1 upregulation has no effect on mitochondrial electron transport chain protein

(A) Rate of ATP hydrolysis assessed by real-time assay of Mg^{2+} efflux-based fluorescence signal using Magnesium green (MgG) in cardiomyocytes during 5µM FCCP treatment (n=4). (B) OCR tracing of cardiomyocytes overexpressing ATPIF1. Data represent the average from 5 independent experiments, each experiment has 4-5 replicate wells. (C) Western blot assay of mitochondrial OXPHOS protein (n=4). (D) Measurement of supercomplex abundance in 50µg isolated mitochondria from cardiomyocytes, using Clear-Native PAGE followed by Coomassie staining and destaining.

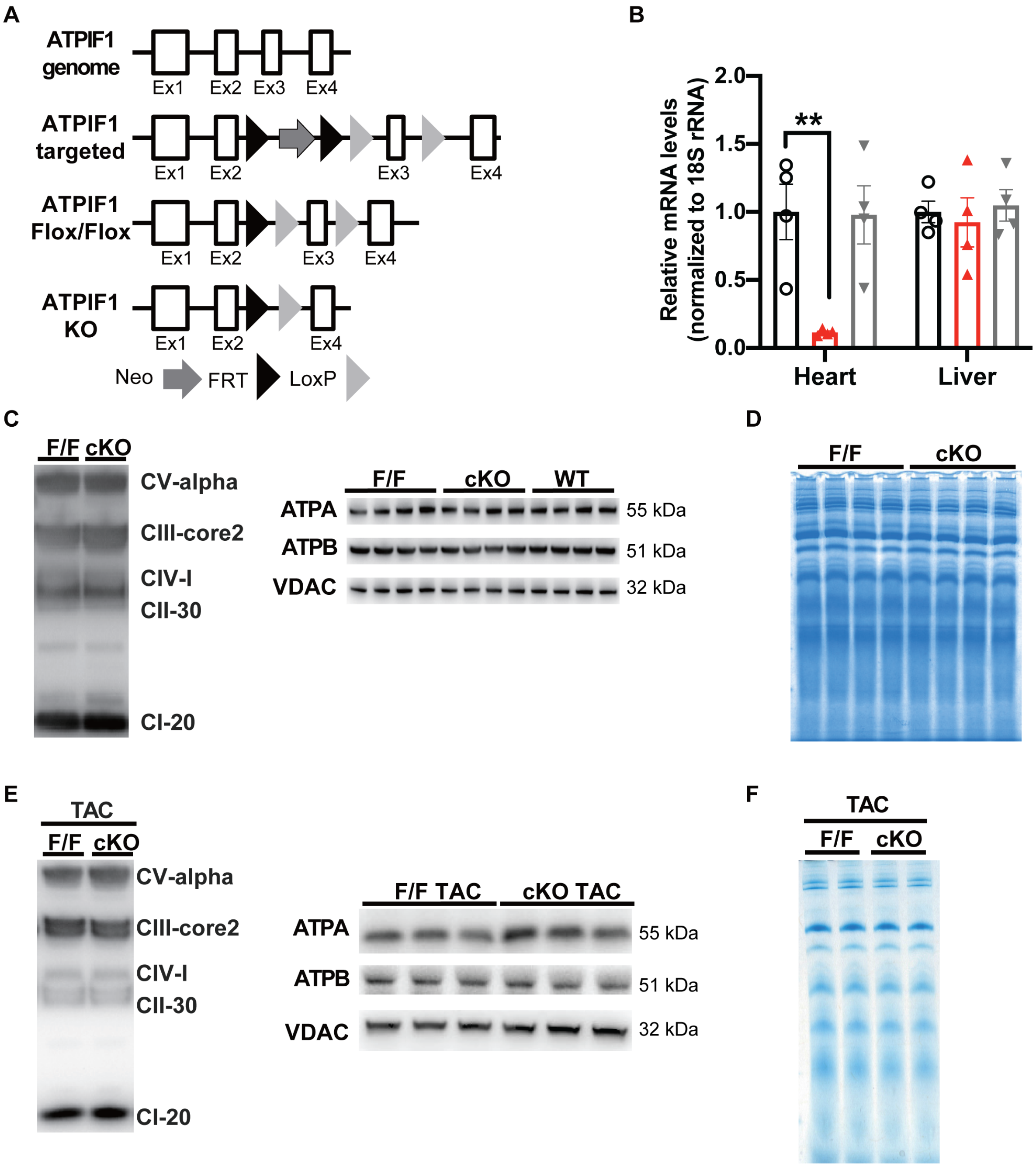

Supplementary 5. Cardiac-specific deletion of ATPIF1 in mice does not change protein levels of the mitochondrial electron transport chain

(A) Strategy to generate cardiac-specific ATPIF1 KO mice is shown. Exon 3 of ATPIF1 was flanked by LoxP sequences and deleted after αMHC-Cre-mediated homologous recombination. (B) Real-time PCR analysis of ATPIF1 expression from liver or ventricular lysates (n=4-5). (C

and D) Representative immunoblot analysis of OXPHOS protein (C, n=4), α and β subunit of F_oF_1-ATP synthase (C, n=3-4) and measurement of supercomplex abundance (D, n=4) using clear-Native PAGE followed by Coomassie staining and destaining in isolated mitochondria. (E and F) Representative immunoblot analysis of OXPHOS protein (E, n=6) and measurement of supercomplex abundance (F, n=3-4) using clear-Native PAGE followed by Coomassie staining and destaining in isolated mitochondria at 28 days after TAC surgery. 18S rRNA was served as mRNA internal control. VDAC was used as protein loading control. Data are means ± SEM of the values. P values were determined using one-way ANOVA followed by Dunnett's multiple comparison (F); $^{**}p<0.01$.

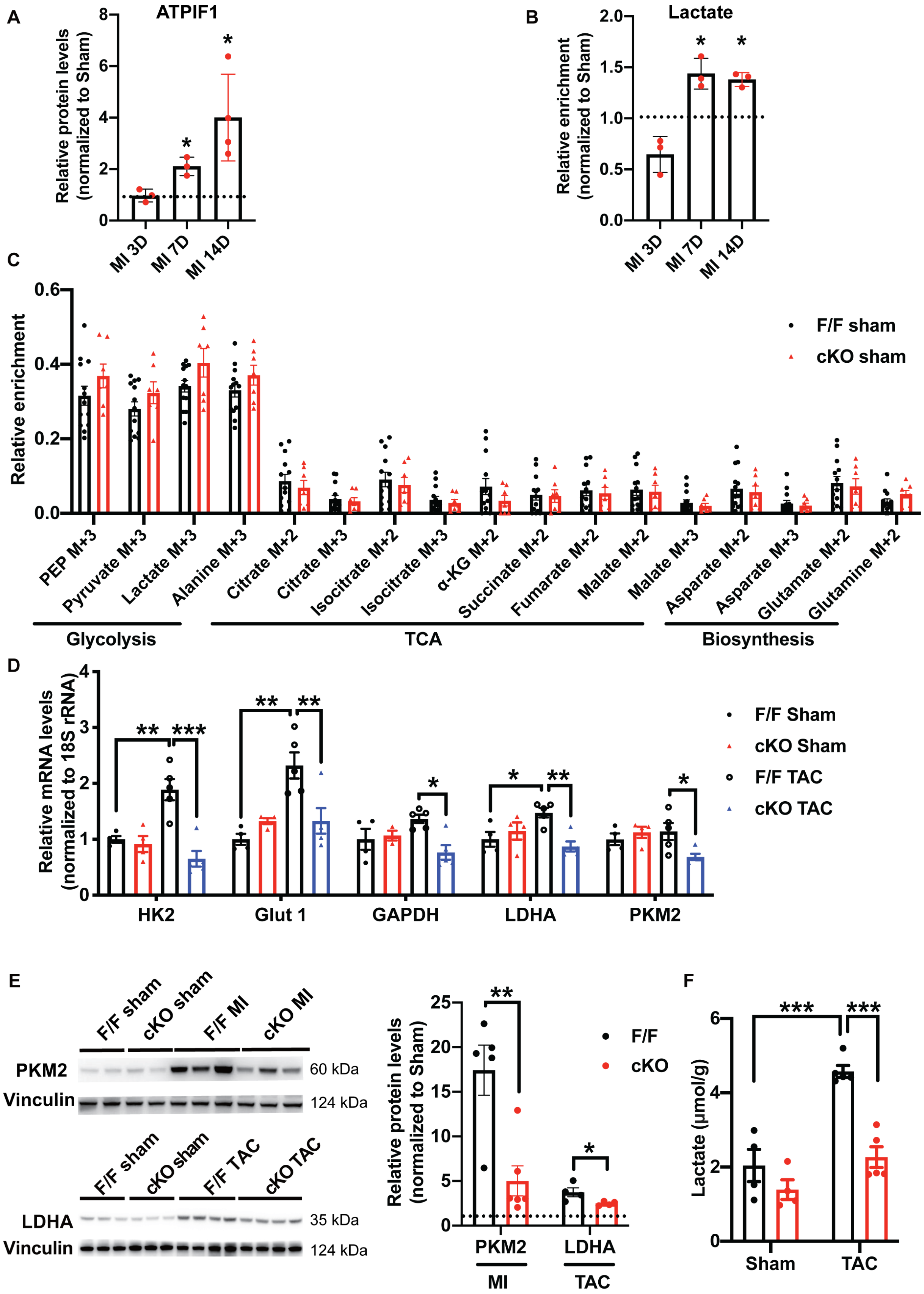
A
ATPIF1
Relative protein levels (normalized to Sham)
MI 3D
MI 7D
MI 14D
B
Lactate
Relative enrichment (normalized to Sham)
C
Relative enrichment
F/F sham
cKO sham
PEP M+3
Pyruvate M+3
Lactate M+3
Alanine M+3
Citrate M+2
Citrate M+3
Isocitrate M+2
Isocitrate M+3
α-KG M+2
Succinate M+2
Fumarate M+2
Malate M+2
Malate M+3
Asparate M+2
Asparate M+3
Glutamate M+2
Glutamine M+2
Glycolysis
TCA
Biosynthesis
D
Relative mRNA levels (normalized to 18S rRNA)
F/F Sham
cKO Sham
F/F TAC
cKO TAC
HK2
Glut 1
GAPDH
LDHA
PKM2
E
F/F sham
cKO sham
F/F MI
cKO MI
PKM2
60 kDa
Vinculin
124 kDa
F/F TAC
cKO TAC
LDHA
35 kDa
Relative protein levels (normalized to Sham)
F/F
cKO
MI
TAC
F
Lactate (μmol/g)
Sham

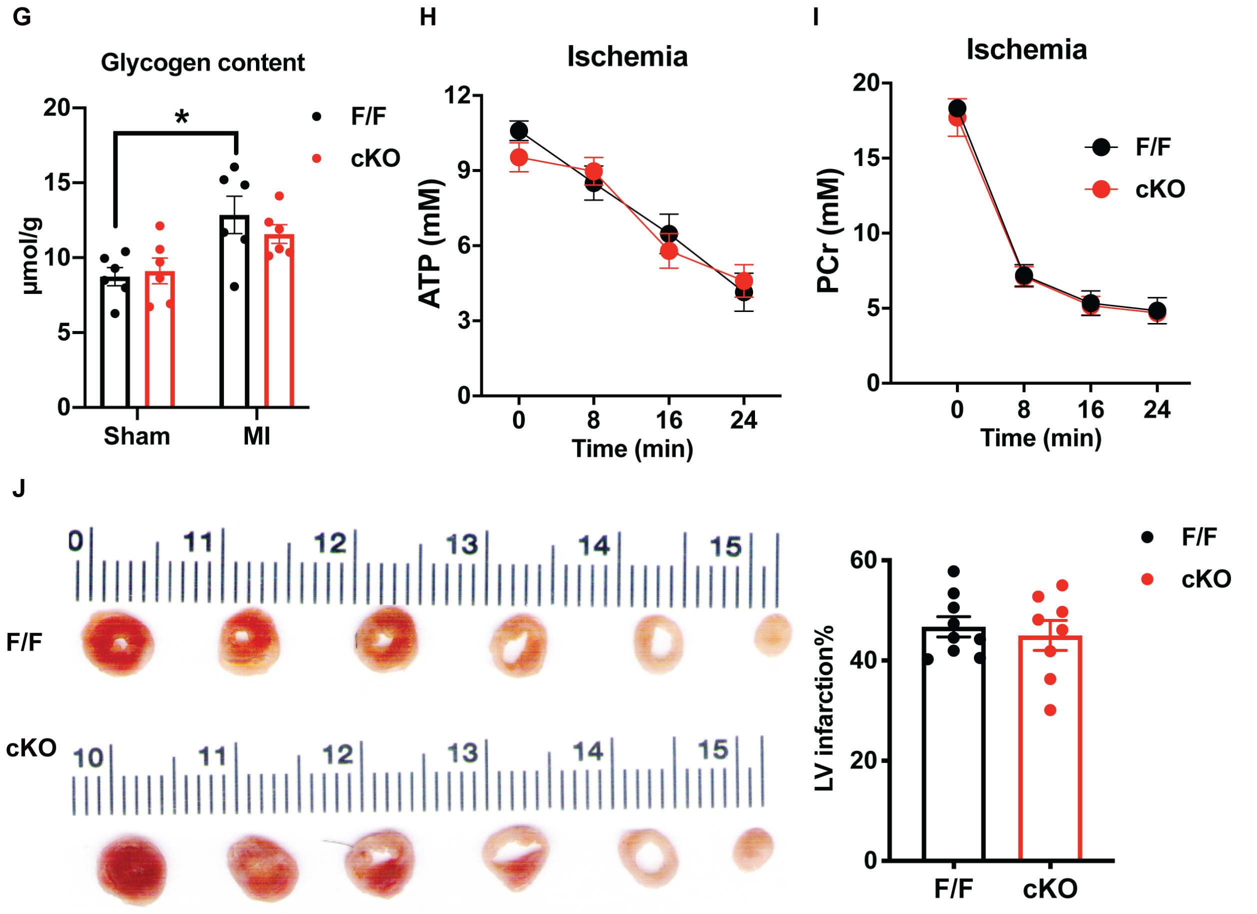

Supplementary 6 Cardiac-specific deletion of ATPIF1 prevented the switch to increased glycolysis induced by pathological cardiac hypertrophy

(A) Immunoblot analysis for ATPIF1 protein expression at day 3, 7 and 14 after MI. (B) Quantification of lactate level in the ventricular tissue at day 3, 7 and 14 after MI by GC/MS (n=3). (C) Relative ^{13}C enrichment of glycolysis, TCA and anaplerosis metabolites from F/F and cKO ventricular tissue at day 7 after sham operation (n=7-13). (D, E and F) Real-time PCR assay of glycolytic genes (D, n=4-5), immunoblot analysis for PKM2 and LDHA protein expression (E, n=3-6), quantification of lactate level (F, n=4-5) in ventricular lysates 14 days after TAC or MI and sham operation. (G) Glycogen content in the ventricular tissue 7 days after MI or sham surgery (n=6). (H and I) ATP (H) and Phosphocreatine concentration (I) measured by ^{31}P NMR

spectroscopy in isolated perfused hearts before and during global no-flow ischemia (n=11). (J) Representative image of heart sections and percentage of infarcted area to total left ventricular area 3 days after MI surgery. 18S rRNA was served as mRNA internal control. Vinculin was used as protein loading control. Data are means ± SEM of the values. P values were determined using Student unpaired t-test (A, B and E) or two-way ANOVA followed by Tukey's multiple comparison test (D, F and G); $*p < 0.05$; $**p<0.01$; $***p<0.001$.

B) Supplemental Table:

Table 1. Quantitative analysis of the crosslinks by pair-wise comparison of TAC and sham hearts.

Protein XLs	Peptide XLs	T1/S1	T2/S2	T3/S3	T4/S4	T5/S5
ATPA$_{427}$_ATPA$_{498}$	AMKQVAGTMK_<br>GYLDKLEPSK	-0.04<br>(0.259)		0<br>(0.363)	-0.02<br>(0.3)	-0.06<br>(0.132)
ATPA$_{427}$_ATPA$_{531}$	AMKQVAGTMK_<br>SDGKISEQSDAK	0.24<br>(0.039)	0.36<br>(0.091)	0.07<br>(0.074)	0.13<br>(0.106)	0.25<br>(0.08)
ATPA$_{427}$_ATPA$_{531}$	AMKQVAGTMK_<br>SDGKISEQSDAKLK	-0.03	0.45	0.77	0.19	0.06<br>(0.17)
ATPA$_{427}$_ATPA$_{531}$	AMKQVAGTM(Ox)K_<br>SDGKISEQSDAK	-0.12<br>(0.346)	0.34<br>(0.182)	0.38<br>(0.166)	0.03<br>(0.108)	0.34<br>(0.076)
ATPA$_{427}$_ATPA$_{531}$	AM(Ox)KQVAGTMK_<br>SDGKISEQSDAK	0.17<br>(0.069)	0.16<br>(0.12)	0.04<br>(0.121)	0.24<br>(0.071)	0.22<br>(0.082)
ATPA$_{427}$_ATPA$_{531}$	AM(Ox)KQVAGTM(Ox)K_<br>SDGKISEQSDAK	0.17<br>(0.387)	0.47<br>(0.308)	0.13<br>(0.117)	0.14<br>(0.099)	0.29<br>(0.161)

$ATPA_{531}$_$ATPA_{427}$	SDGKISEQSDAK_ AMKQVAGTMKLELAQYR	-0.26 (0.039)	0.66 (0.255)	0.06 (0.18)	0.59 (0.638)	0.36 (0.11)
$ATPA_{531}$_$ATPA_{427}$	SDGKISEQSDAK_ AMKQVAGTMKLELAQYREVA AFAQFGSDLDAATQQLLSR				0.52	
$ATPA_{531}$_$ATPA_{427}$	SDGKISEQSDAK_ AMKQVAGTM(Ox)KLELAQYR		0.4 (0.625)	0.23 (0.149)	0.18 (0.175)	0.65
$ATPA_{531}$_$ATPA_{427}$	SDGKISEQSDAK_ AM(Ox)KQVAGTMKLELAQYR				0.52 (0.638)	
$ATPA_{531}$_$ATPA_{434}$	SDGKISEQSDAK_ AMKQVAGTMKLELAQYREVA AFAQFGSDLDAATQQLLSR				0.47	
$ATPA_{531}$_$ATPA_{434}$	SDGKISEQSDAK_ QVAGTMKLELAQYR	0.39 (0.141)	0.2 (0.096)	0.07 (0.08)	0.34 (0.069)	0.42 (0.065)
$ATPA_{531}$_$ATPA_{434}$	SDGKISEQSDAK_ QVAGTM(Ox)KLELAQYR	0.22 (0.376)	-0.1 (0.185)	0.41 (0.055)	0.38 (0.199)	0.39 (0.106)

ATPA$_{498}$_ATPA$_{531}$	GYLDKLEPSK_ SDGKISEQSDAK		0.39 (0.119)	0.13 (0.29)	-0.02 (0.181)	-0.03 (0.079)
ATPB$_{522}$_ATPA$_{434}$	ADKLAEEHGS_ QVAGTMKLELAQYR	0.03 (0.218)	0.49	-0.19 (0.471)	-0.16 (0.164)	-0.26 (0.149)
ATPB$_{522}$_ATPA$_{434}$	ADKLAEEHGS_ QVAGTM(Ox)KLELAQYR	0.12 (0.62)		0.17	-0.33 (0.588)	
ATPB$_{485}$_ATPB$_{522}$	LVPLKETIK_ ADKLAEEHGS	-0.27 (0.117)	0.41 (0.134)	-0.21 (0.226)	-0.62 (0.274)	-0.56 (0.07)
ATPB$_{522}$_ATPA$_{498}$	ADKLAEEHGS_ GYLDKLEPSK	-0.06 (0.103)			-0.51 (0.128)	-0.56 (0.333)
ATPB$_{522}$_ATPA$_{531}$	ADKLAEEHGS_ SDGKISEQSDAK	0.13 (0.265)	0.4 (0.301)	0.02 (0.096)	-0.03 (0.061)	0.09 (0.098)
ATPB$_{485}$_ATPA$_{427}$	LVPLKETIK_ AMKQVAGTMK	0.15 (0.255)	0.48 (0.069)	0.09 (0.074)	-0.09 (0.086)	-0.19 (0.16)
ATPB$_{485}$_ATPA$_{427}$	LVPLKETIK_ AMKQVAGTM(Ox)K	0.04 (0.18)				

$ATPB_{485}$_$ATPA_{531}$	LVPLKETIK_ SDGKISEQSDAK	0.04 (0.028)	0.32 (0.223)	0.03 (0.167)	-0.02 (0.063)	-0.13 (0.041)
$ATPB_{522}$_$ATPA_{427}$	ADKLAEEHGS_ AMKQVAGTMK	-0.07 (0.091)	0.52 (0.098)	0.01 (0.078)	-0.07 (0.054)	0.04 (0.074)
$ATPB_{522}$_$ATPA_{427}$	ADKLAEEHGS_ AMKQVAGTM(Ox)K		0.32 (0.085)	-0.24 (0.203)	-0.03 (0.248)	-0.12 (0.201)
$ATPB_{522}$_$ATPA_{427}$	ADKLAEEHGS_ AM(Ox)KQVAGTMK	0 (0.159)	0.39 (0.118)	0.15 (0.193)	-0.12 (0.045)	-0.09 (0.086)
$ATPB_{522}$_$ATPA_{427}$	ADKLAEEHGS_ AM(Ox)KQVAGTM(Ox)K	0.2 (0.298)	0.52 (0.411)	-0.04 (0.128)	-0.03 (0.136)	-0.11 (0.082)

Log2 ratios of TAC vs sham from 5 biological replicates for crosslinks identified between ATPA dimer, ATPB dimer, ATPA-ATPB. (TAC/sham: T/S; 95% confidence interval for each cross-linked peptide pairs are shown in parenthesis.)

Full unedited gel for Figure 1A

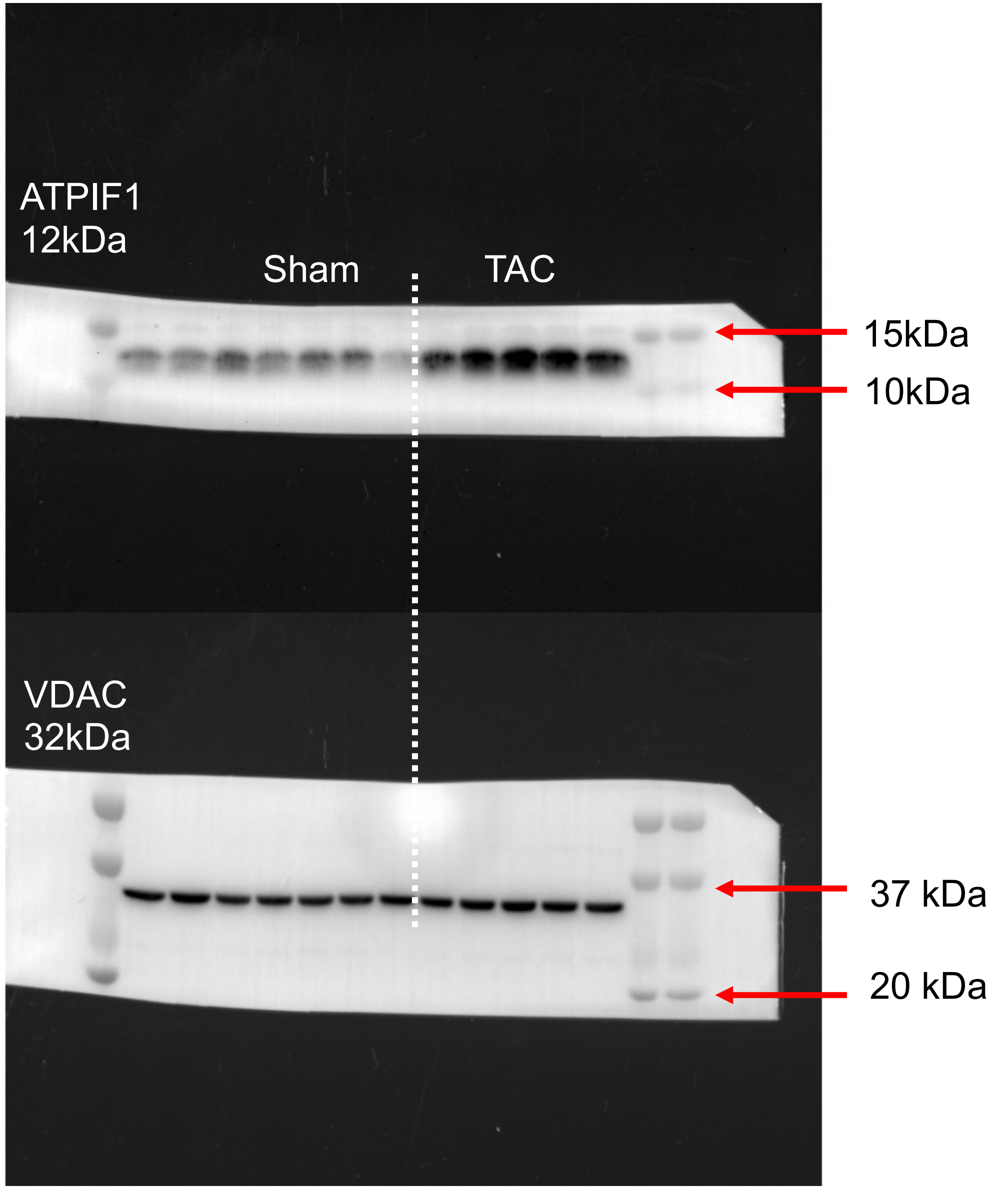

Full unedited gel for Figure 1B

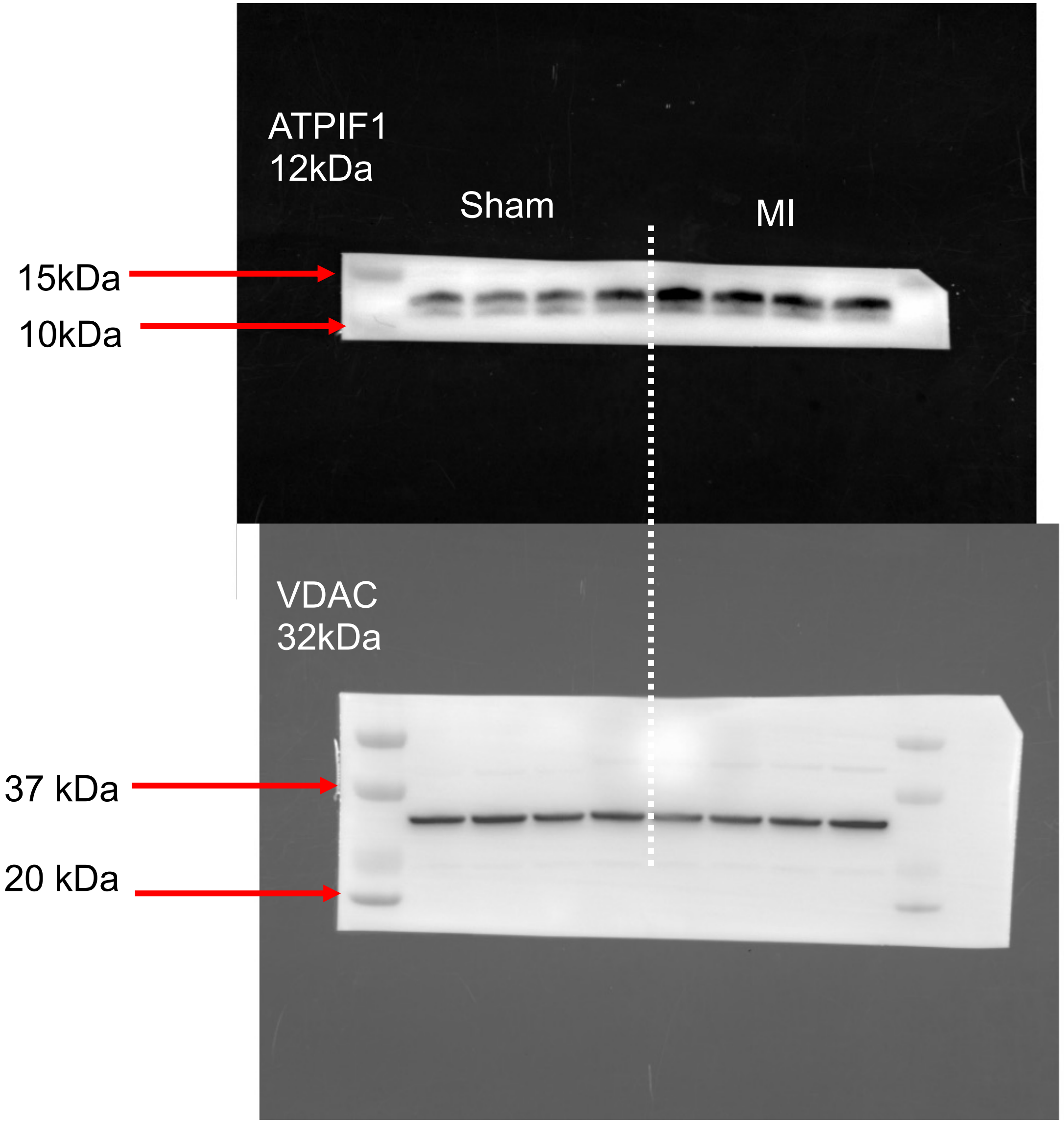

ATPIF1: Cell Signaling Technology 8528
VDAC: Cell Signaling Technology 4661

Full unedited gel for Figure 1C

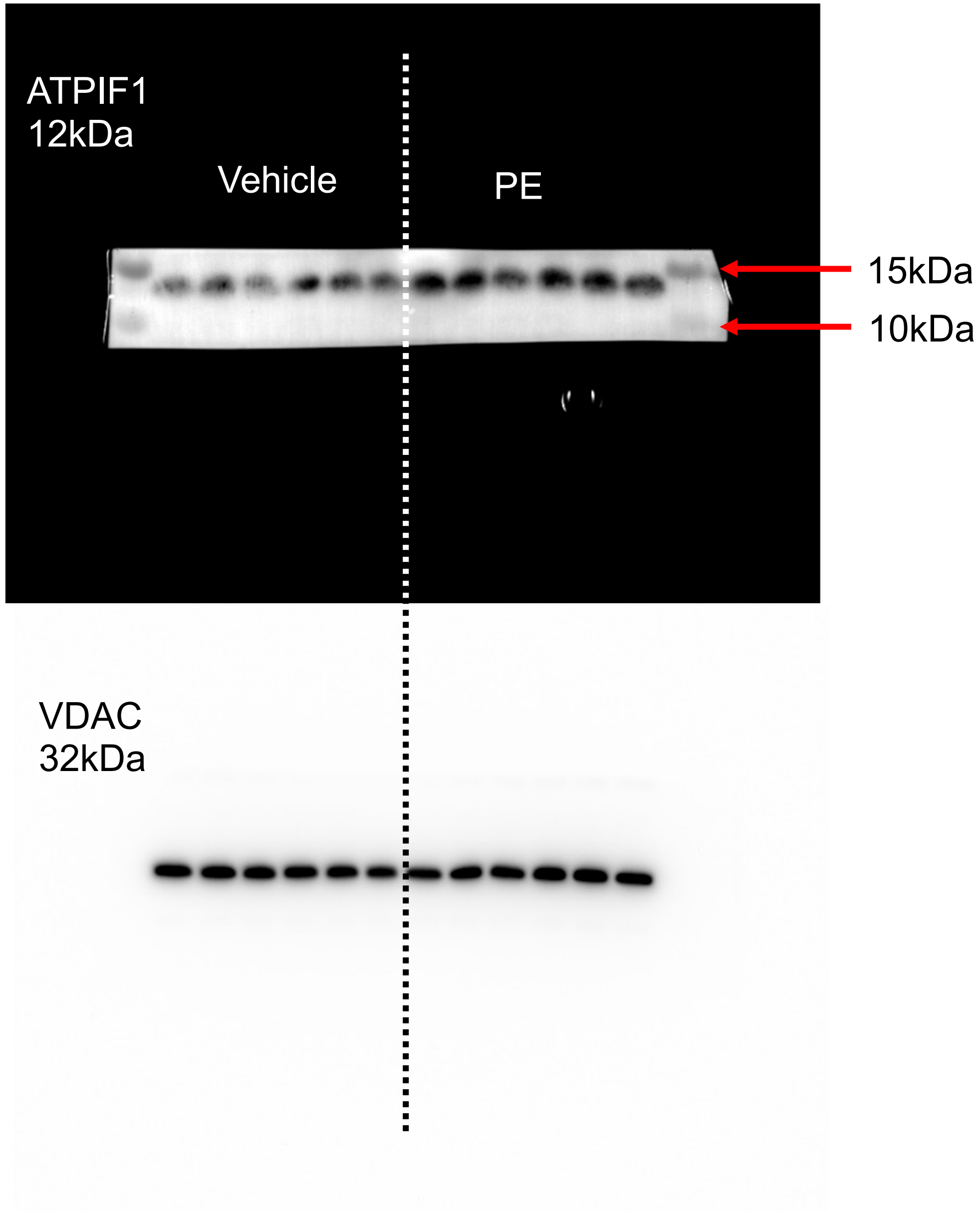

Full unedited gel for Figure 1D

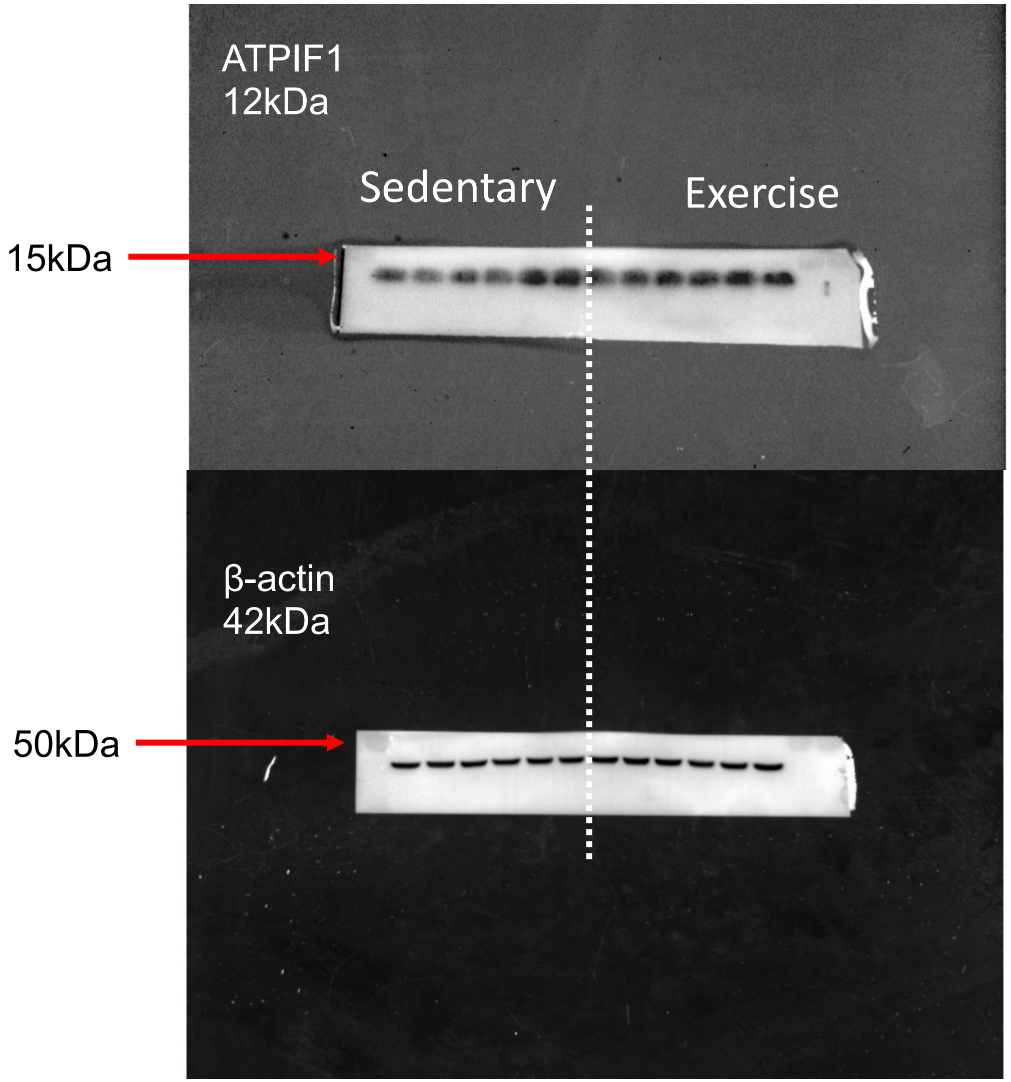

ATPIF1: Cell Signaling Technology 8528
VDAC: Cell Signaling Technology 4661
β-actin: Sigma A2103

Full unedited gel for Figure 1E

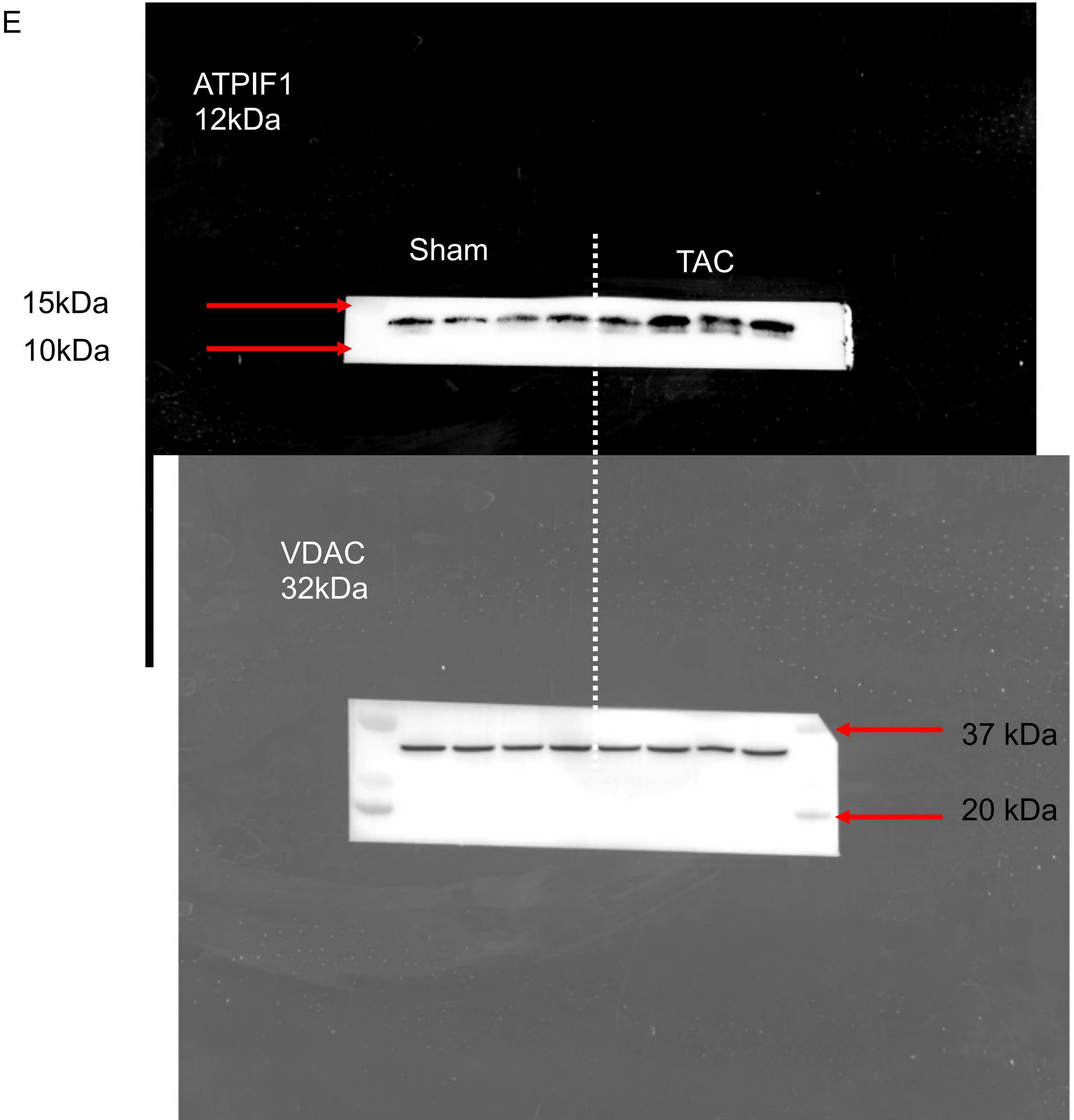

ATPIF1: Cell Signaling Technology 8528
VDAC: Cell Signaling Technology 4661

Full unedited gel for Figure 2C

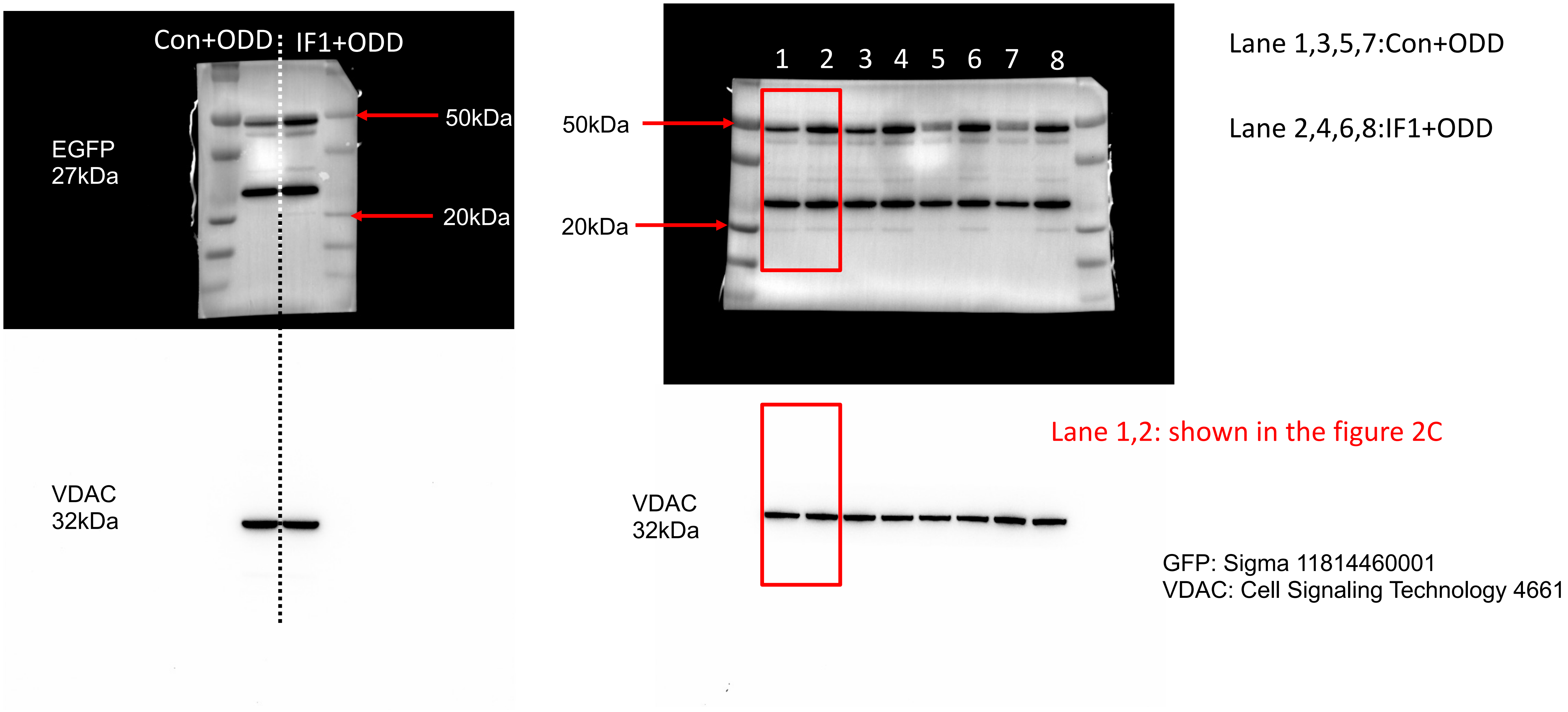

Full unedited gel for Fig 3B

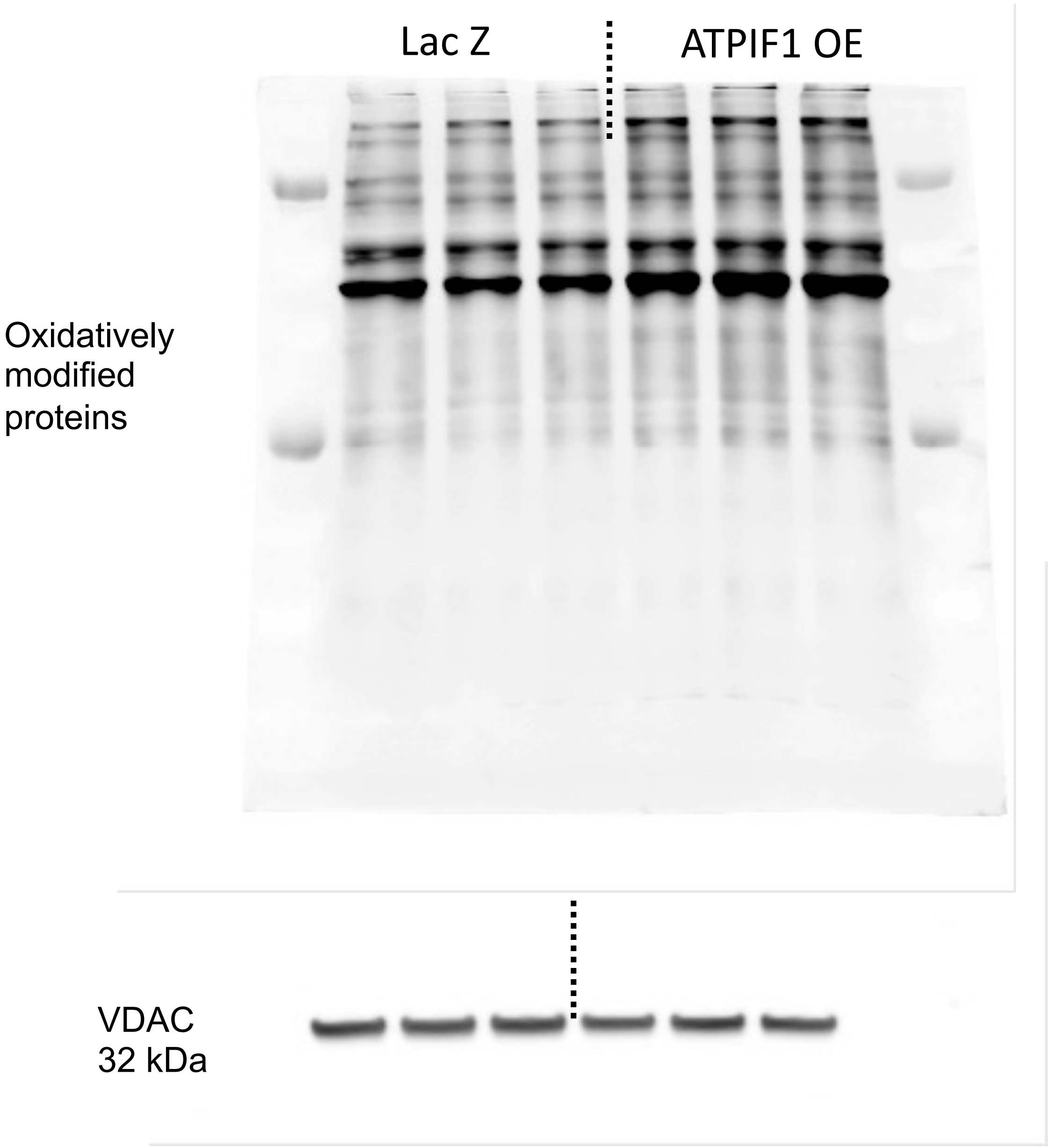

OxyBlot Protein Oxidation Detection Kit : Sigma S7150
VDAC: Cell Signaling Technology 4661

Full unedited gel for Fig 3D

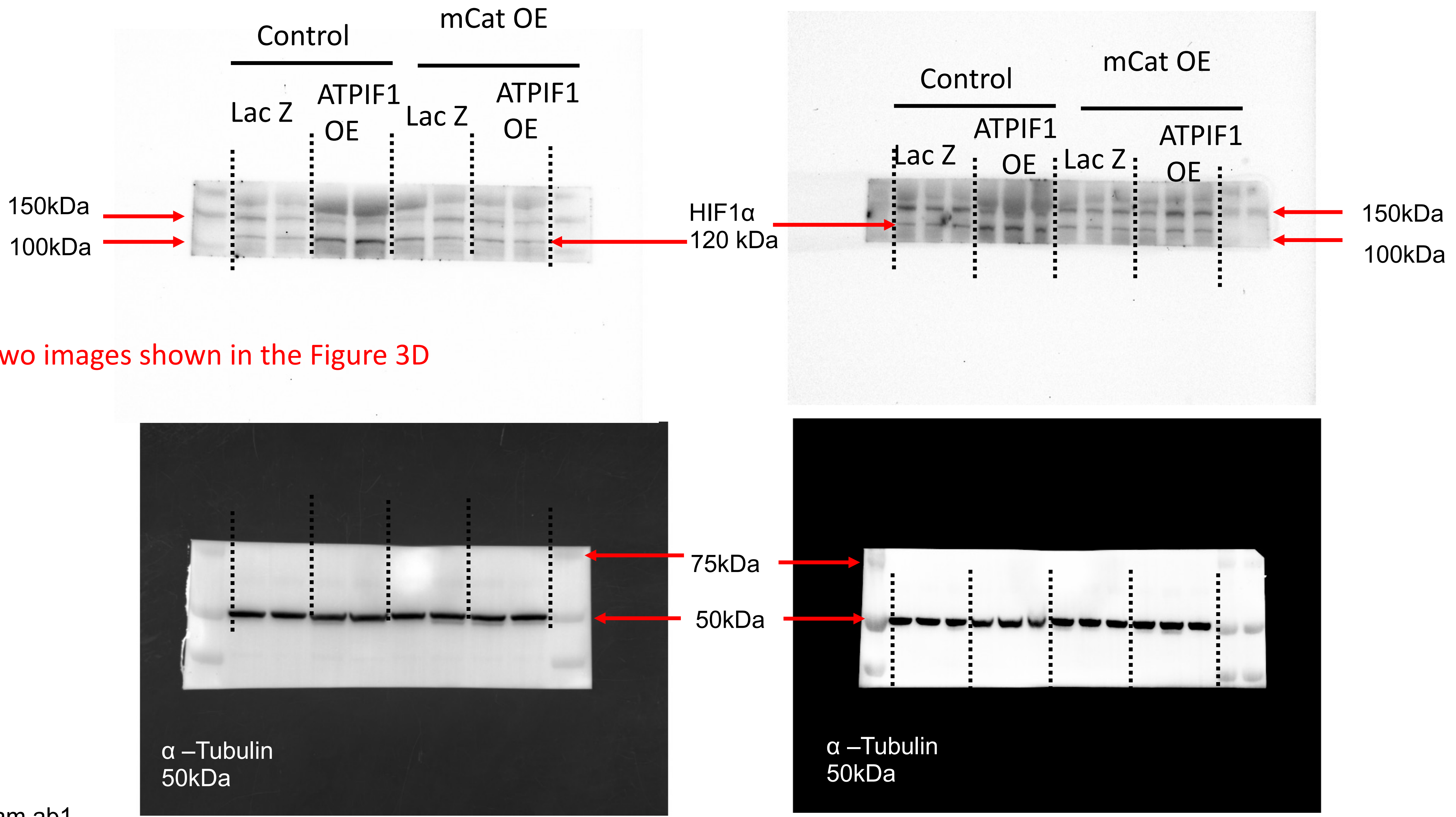

HIF1α: Abcam ab1
α –Tubulin: Sigma T6199

Full unedited gel for Fig 6A

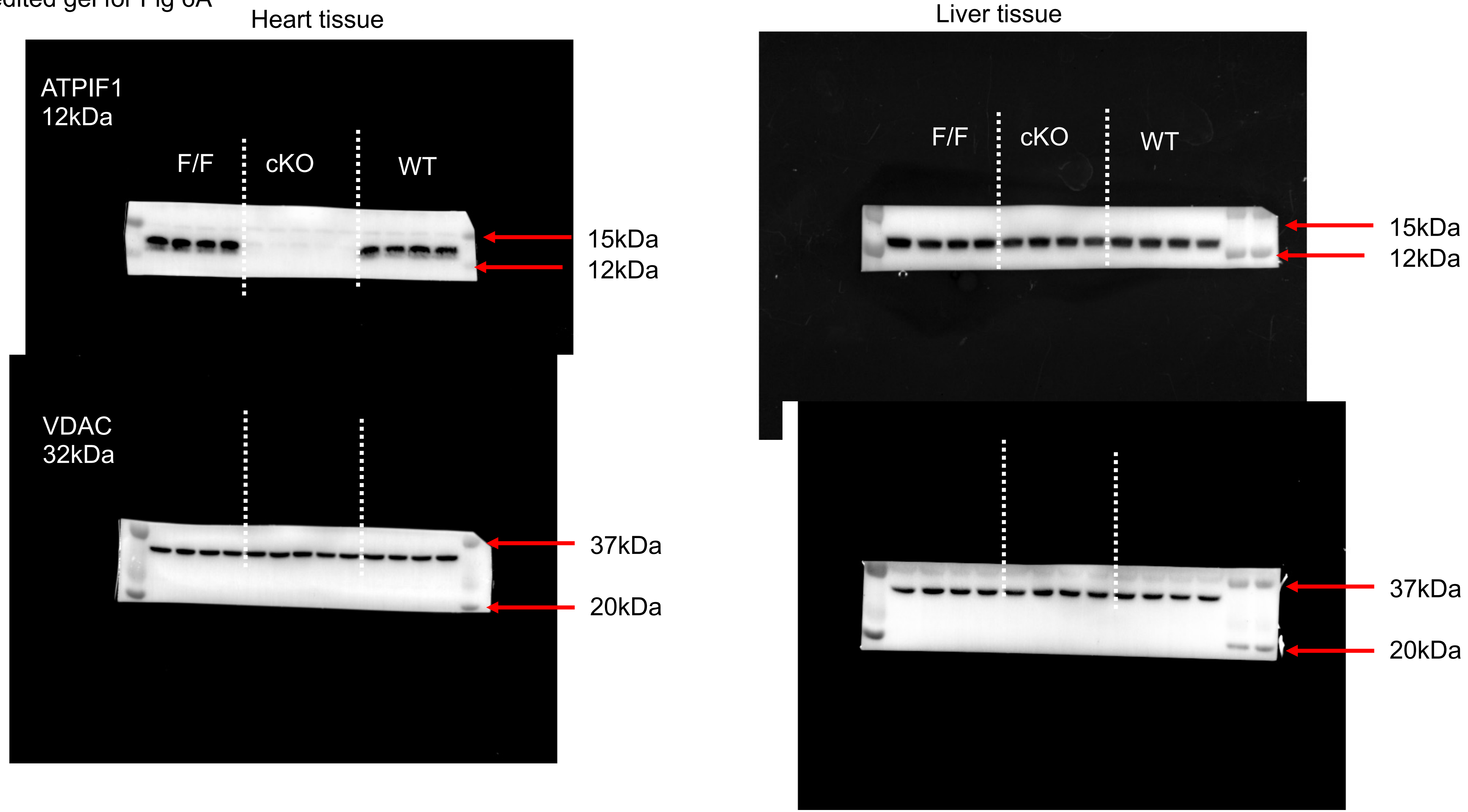

ATPIF1: Cell Signaling Technology 8528
VDAC: Cell Signaling Technology 4661

Full unedited gel for Supplementary 2A

ATPIF1: Cell Signaling Technology 8528
β-actin: Sigma A2103

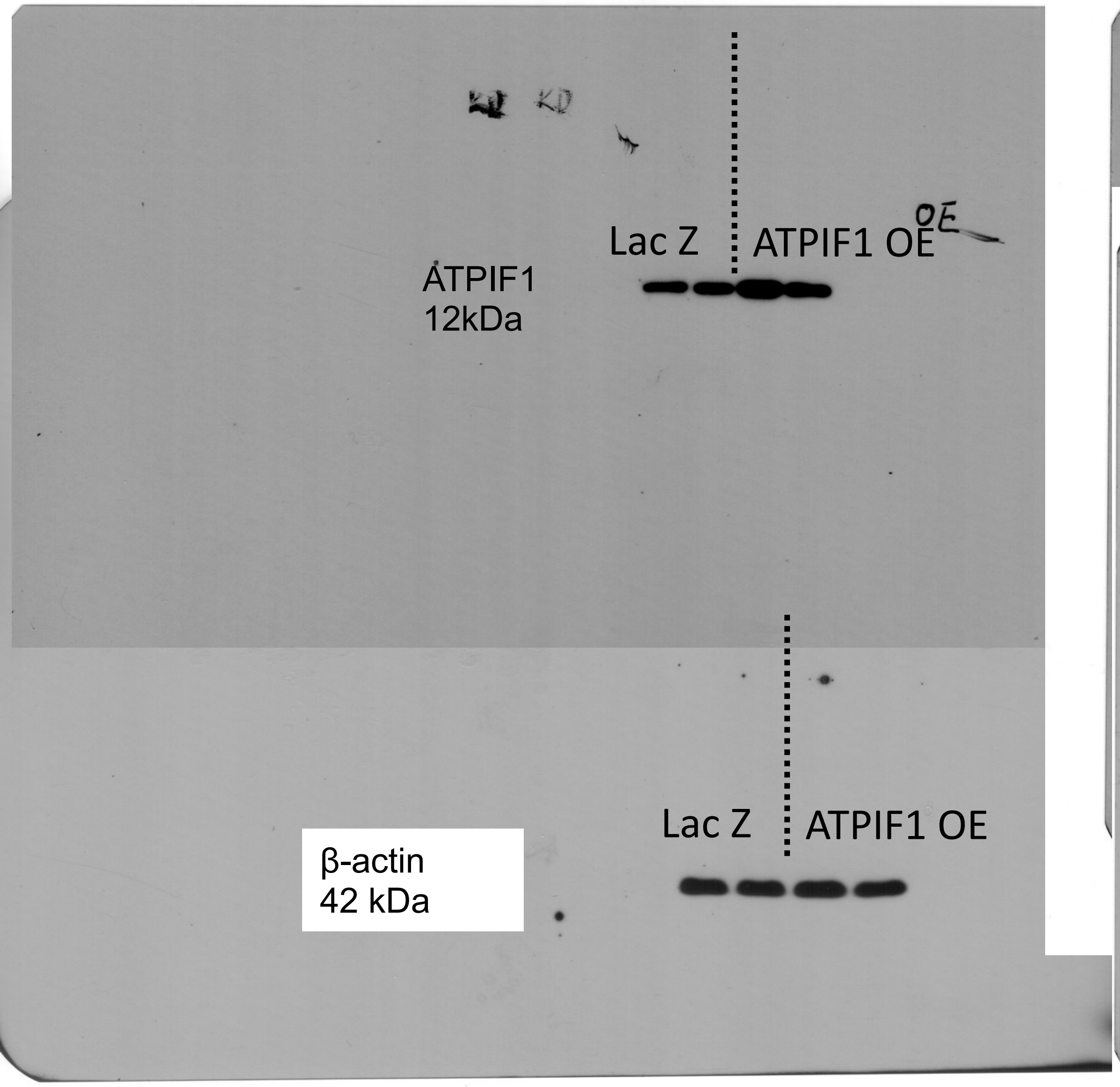

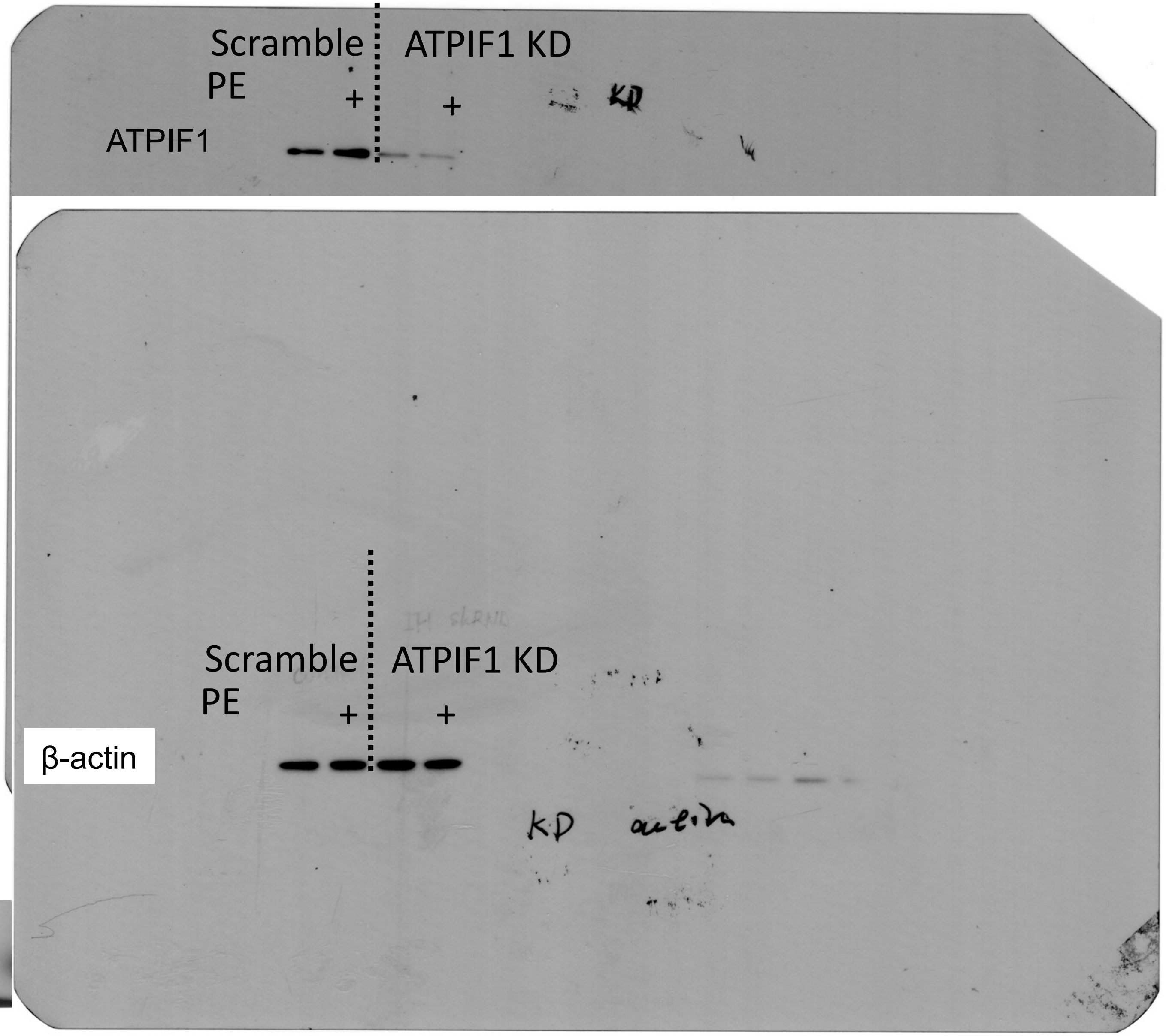

Full unedited gel for Supplementary 2E

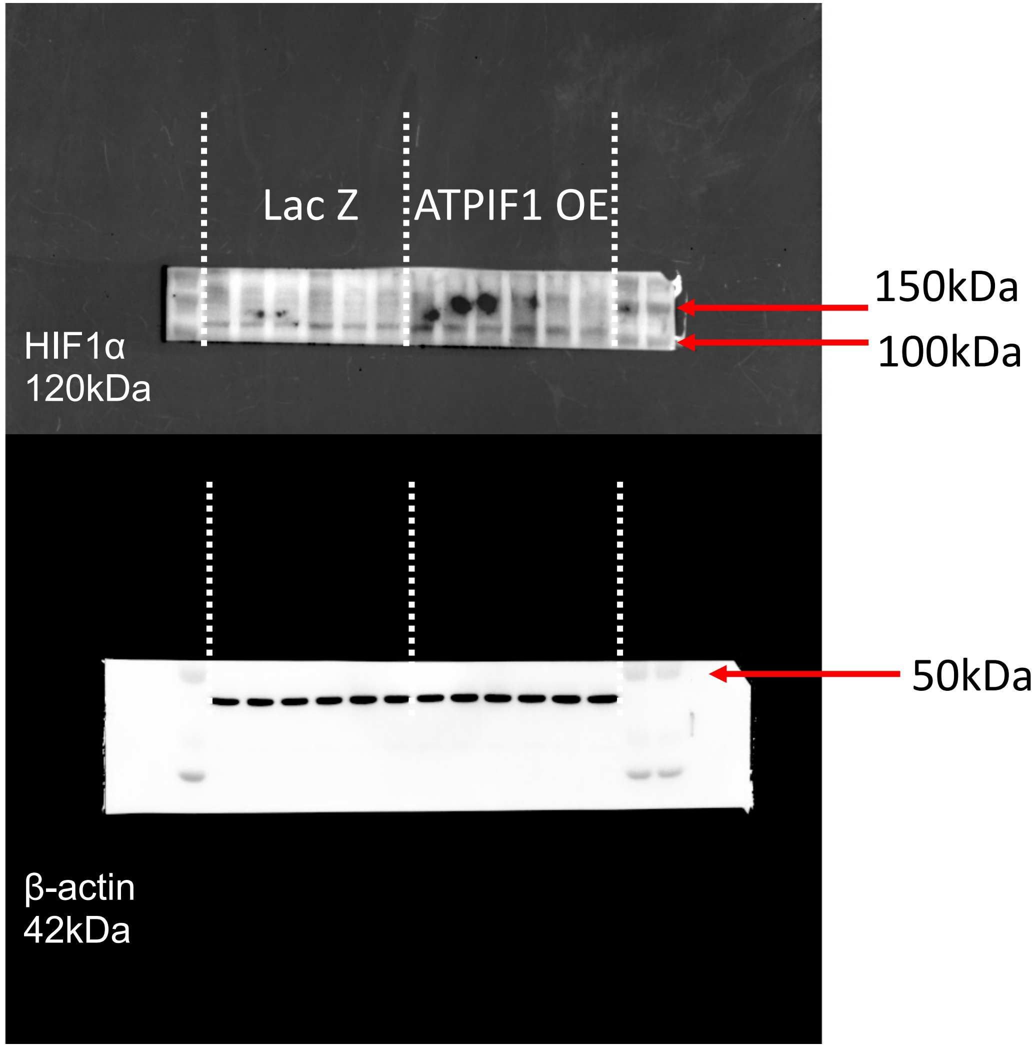

HIF1α: Abcam ab1
β-actin: Sigma A2103

Full unedited gel for Supplementary 2E

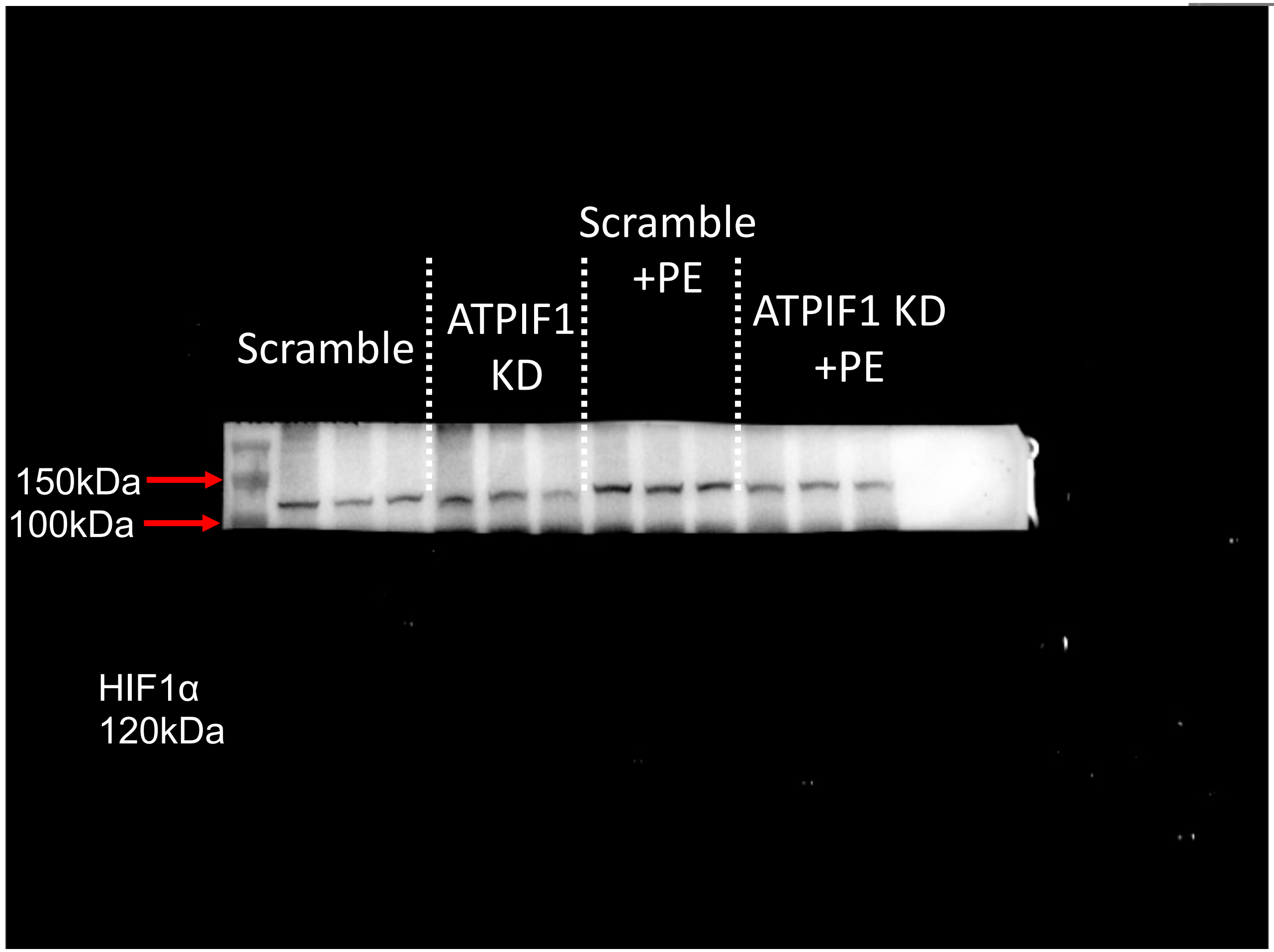

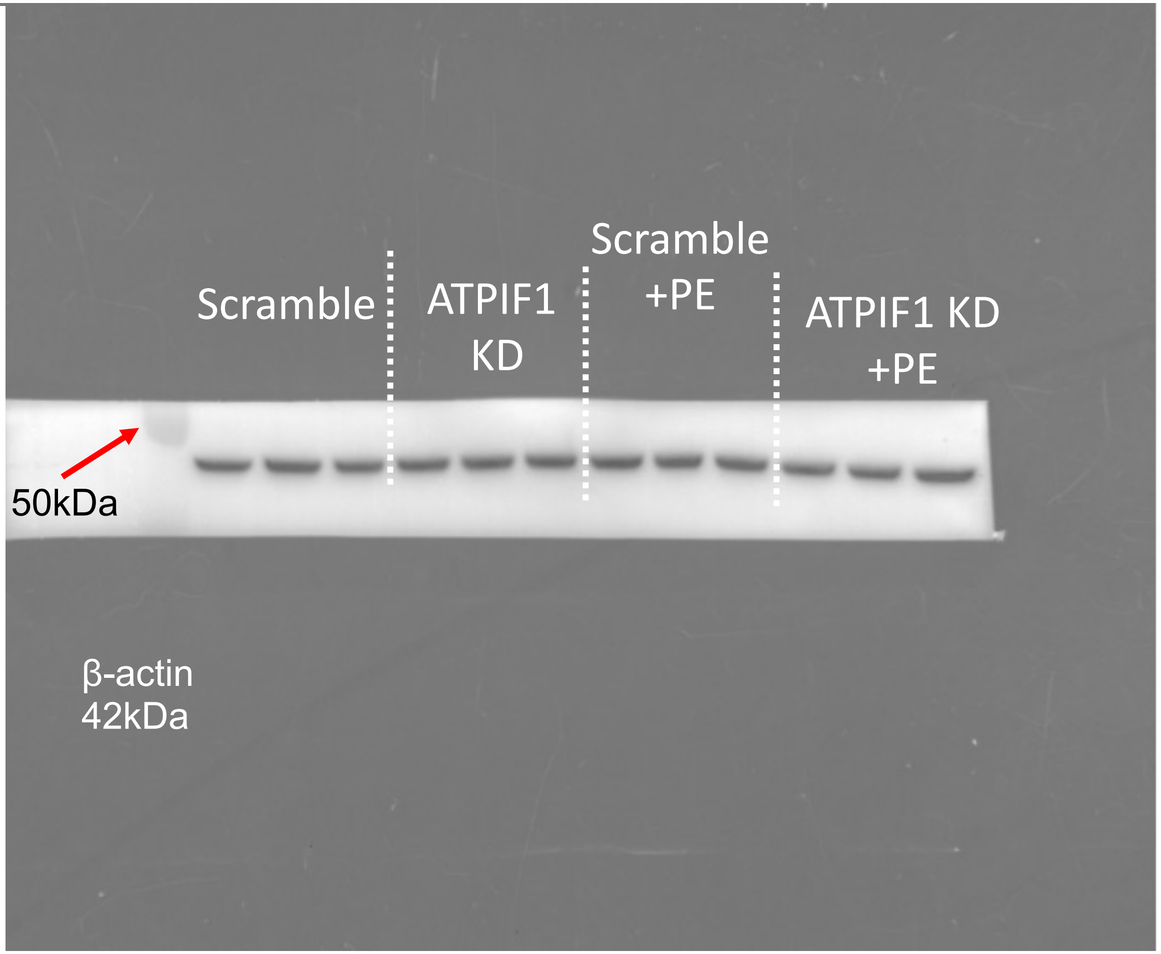

These two images shown in the Supplementary 2E

HIF1α: Abcam ab1
β-actin: Sigma A2103

Full unedited gel for Supplementary 2E

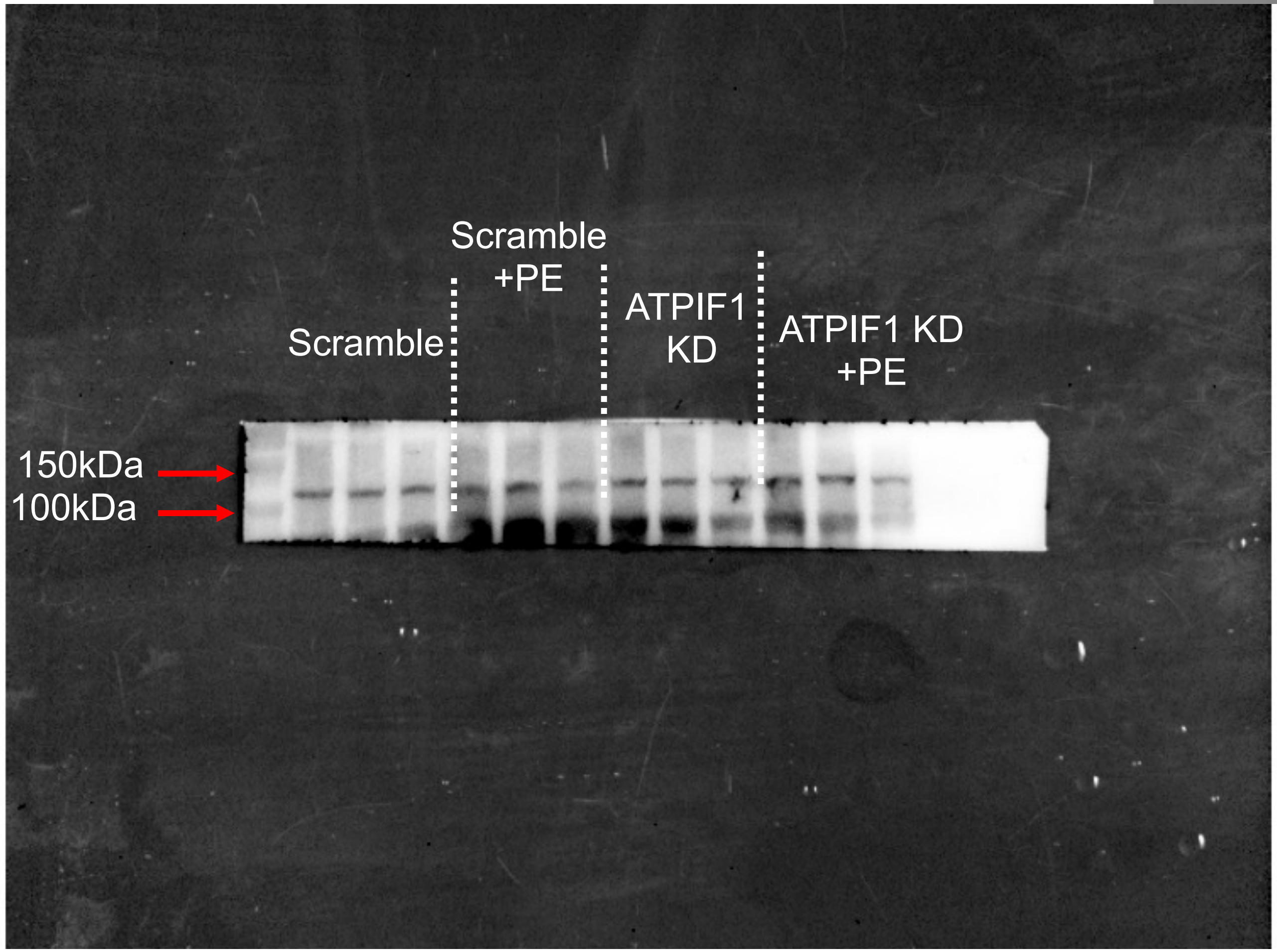

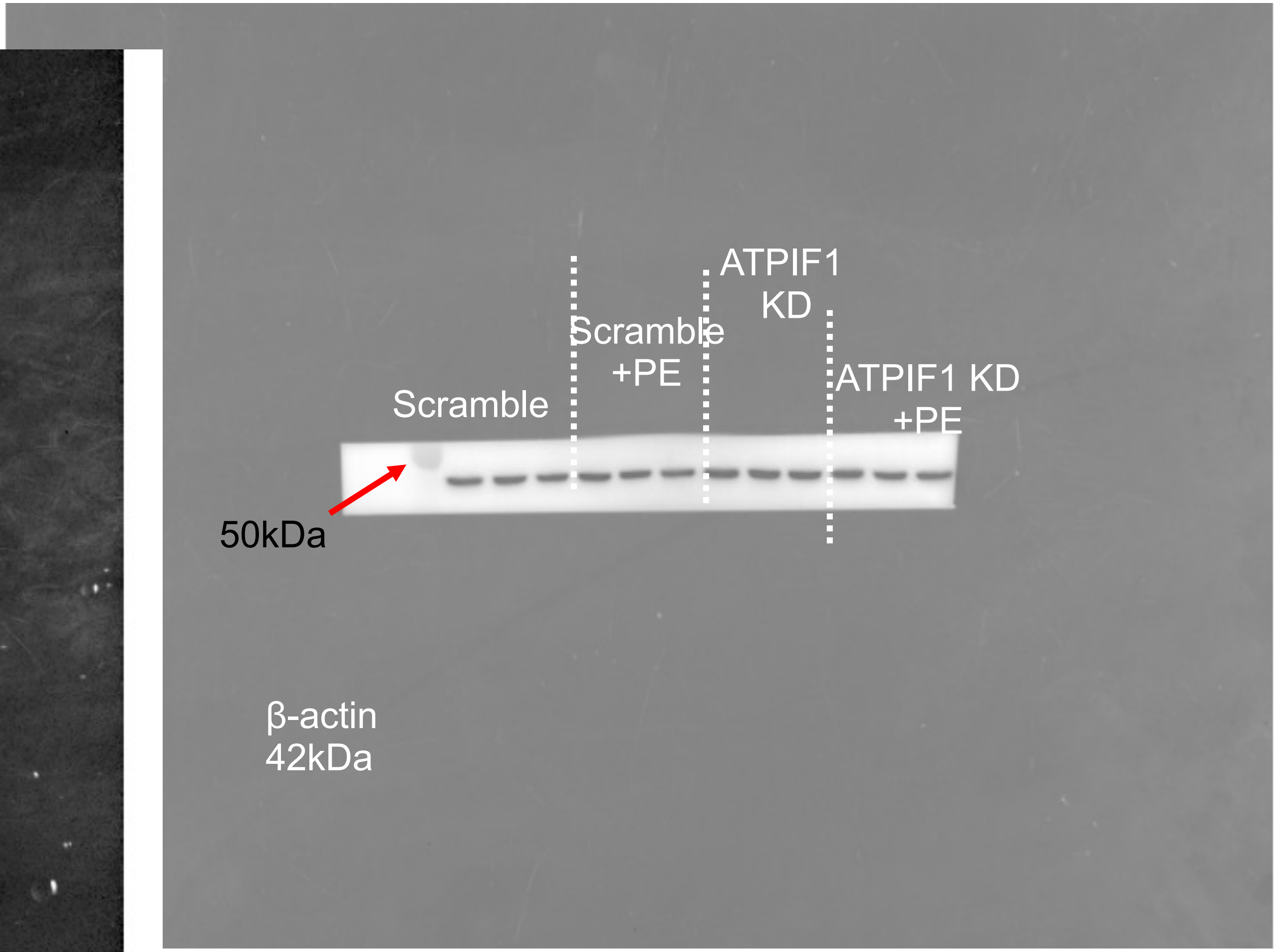

HIF1α: Abcam ab1
β-actin: Sigma A2103

Full unedited gel for Supplementary 2F

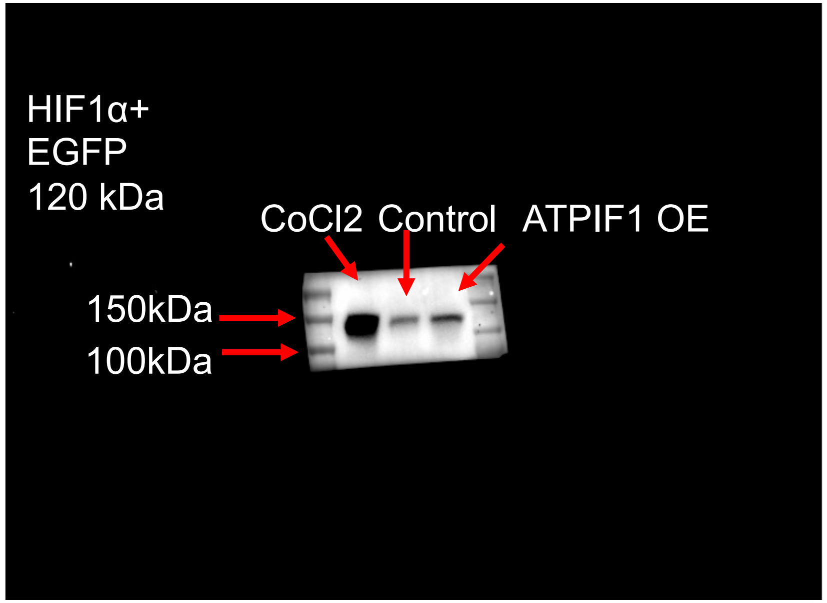

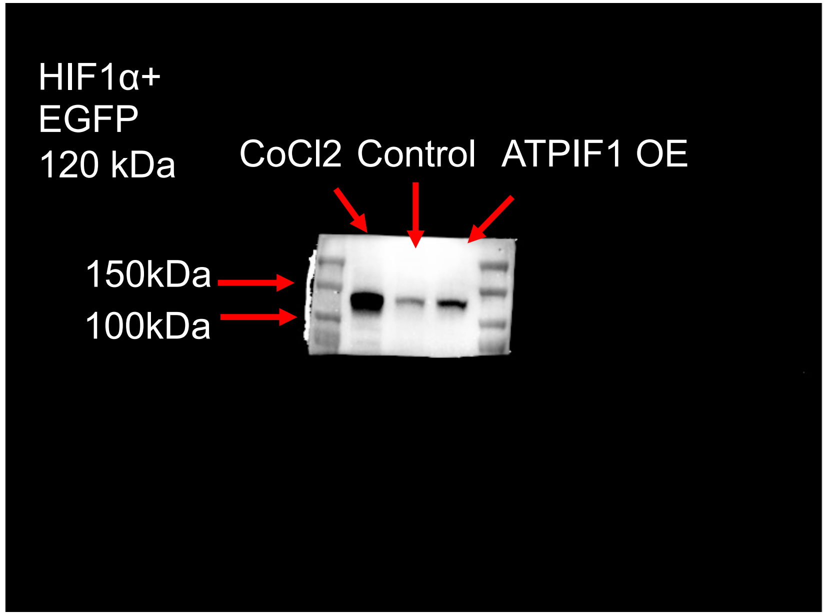

These two images shown in the Supplementary 2F

Histone 3
17kDa

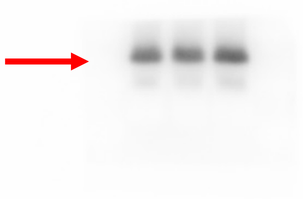

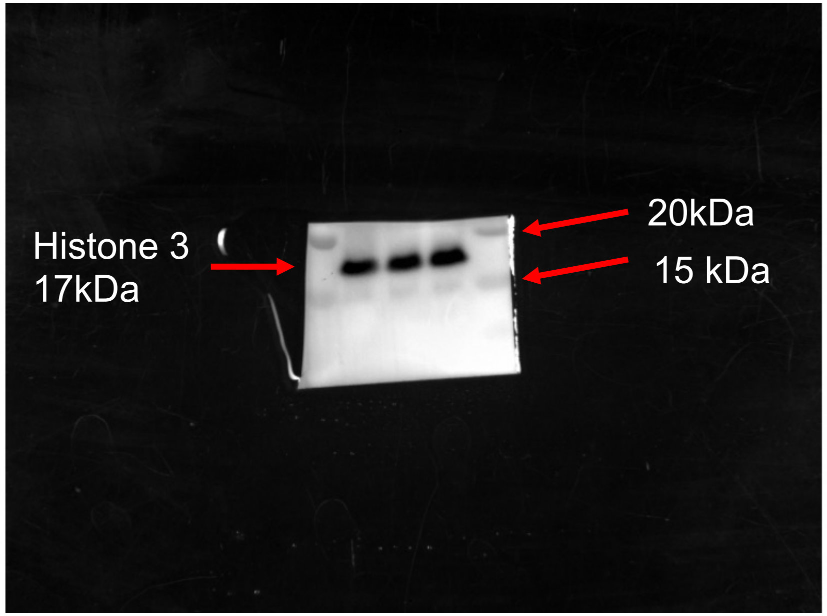

GFP: Sigma 11814460001
Histone 3: Cell Signaling Technology
4499

Full unedited gel for Supplementary 2F

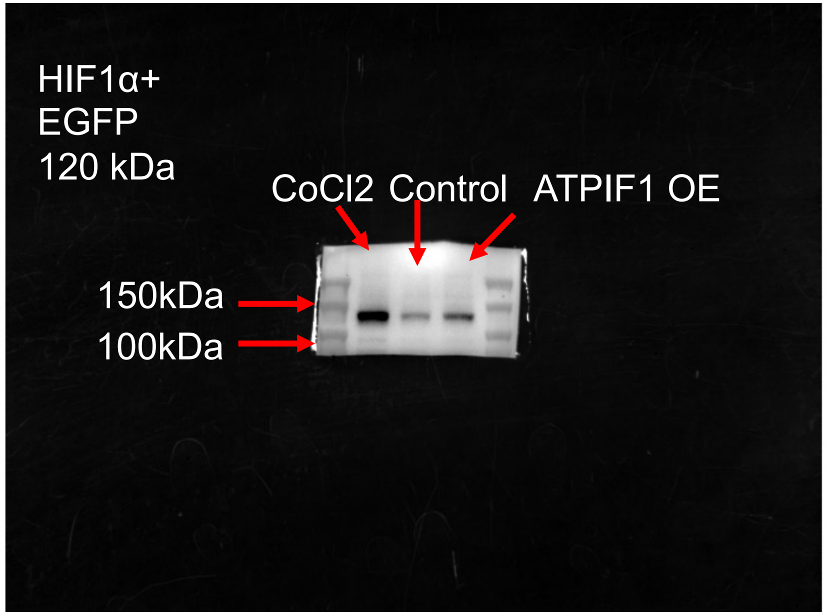

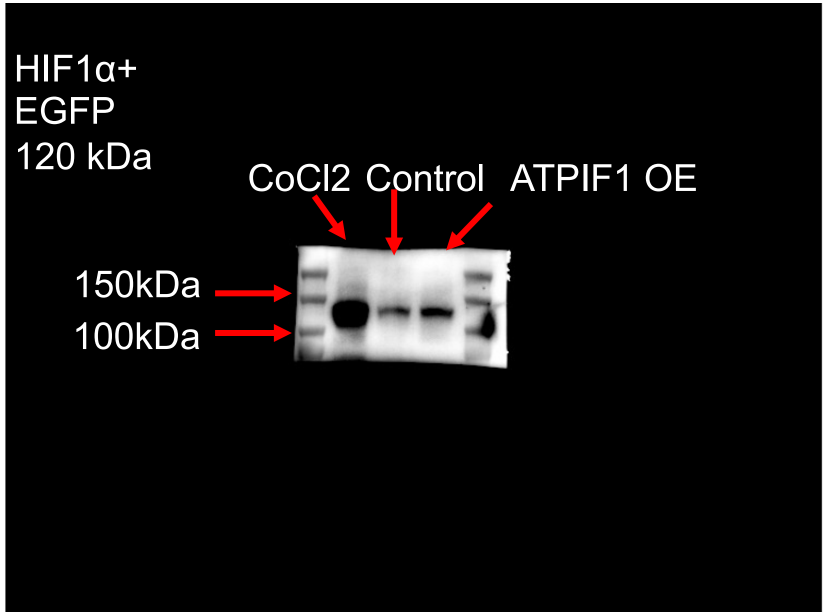

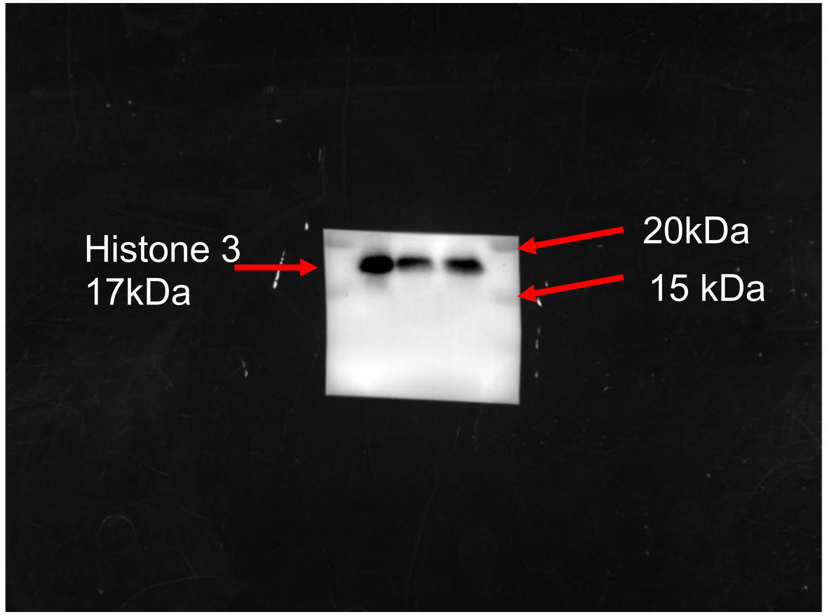

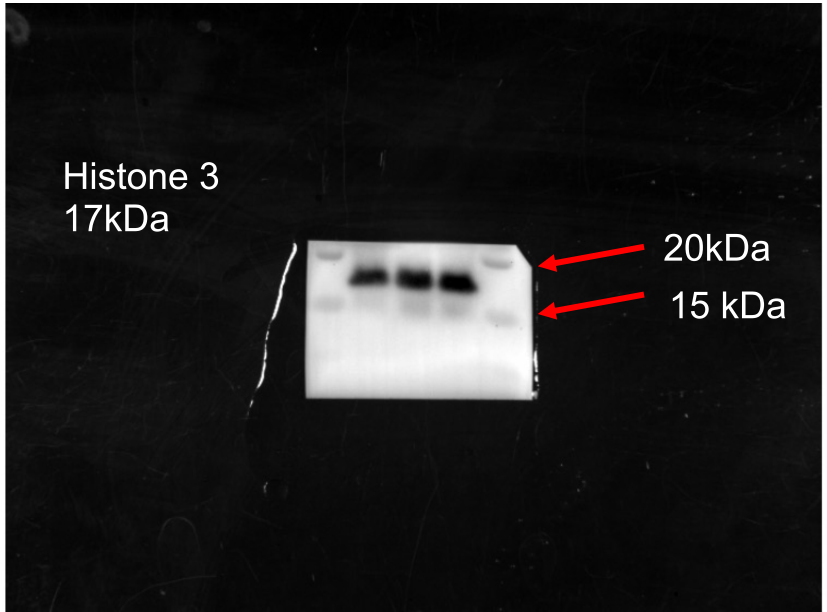

GFP: Sigma 11814460001
Histone 3: Cell Signaling Technology 4499

Full unedited gel for Supplementary 3D

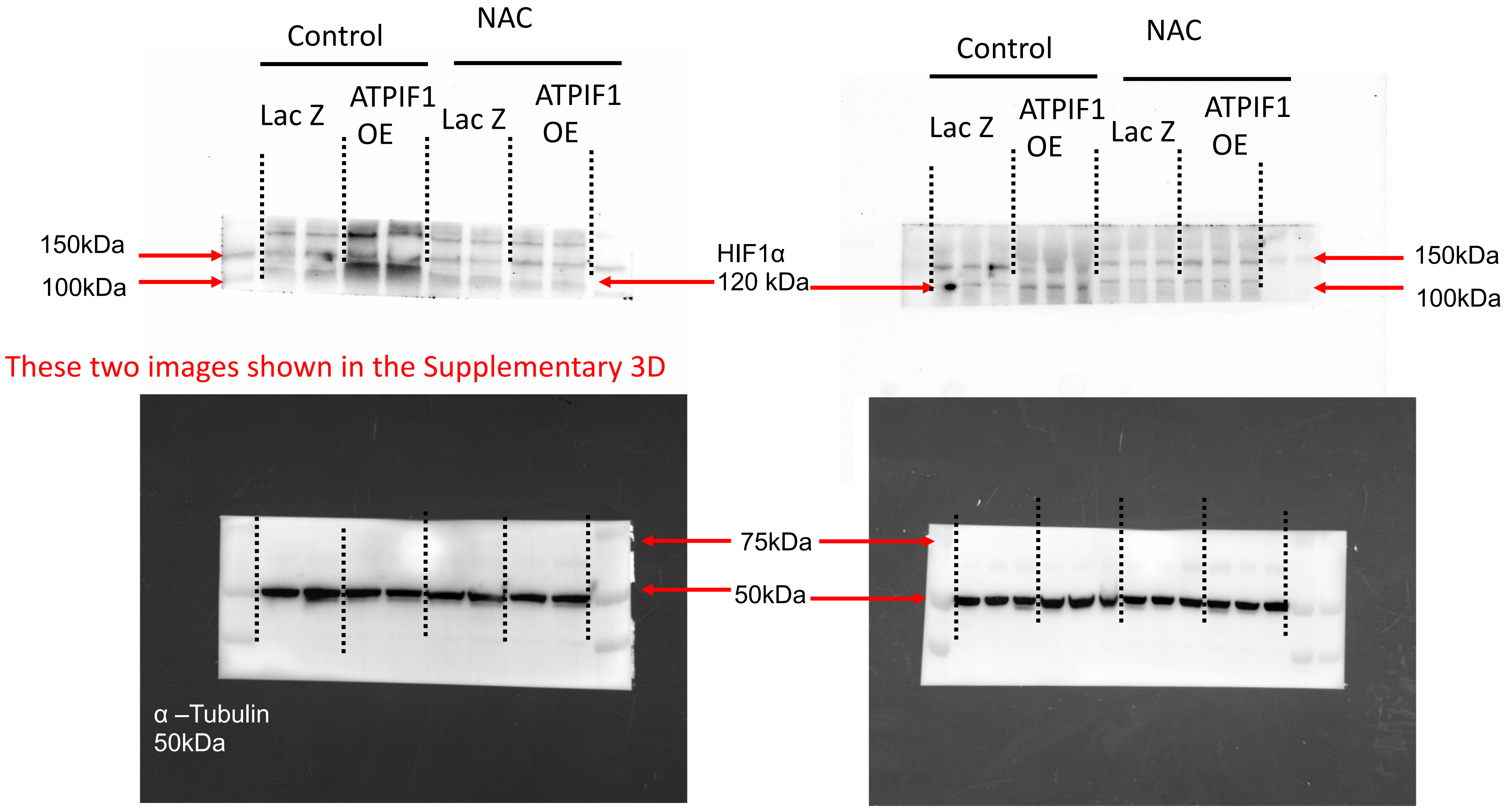

HIF1α: Abcam ab1
α –Tubulin: Sigma T6199

Full unedited gel for Supplementary 4C

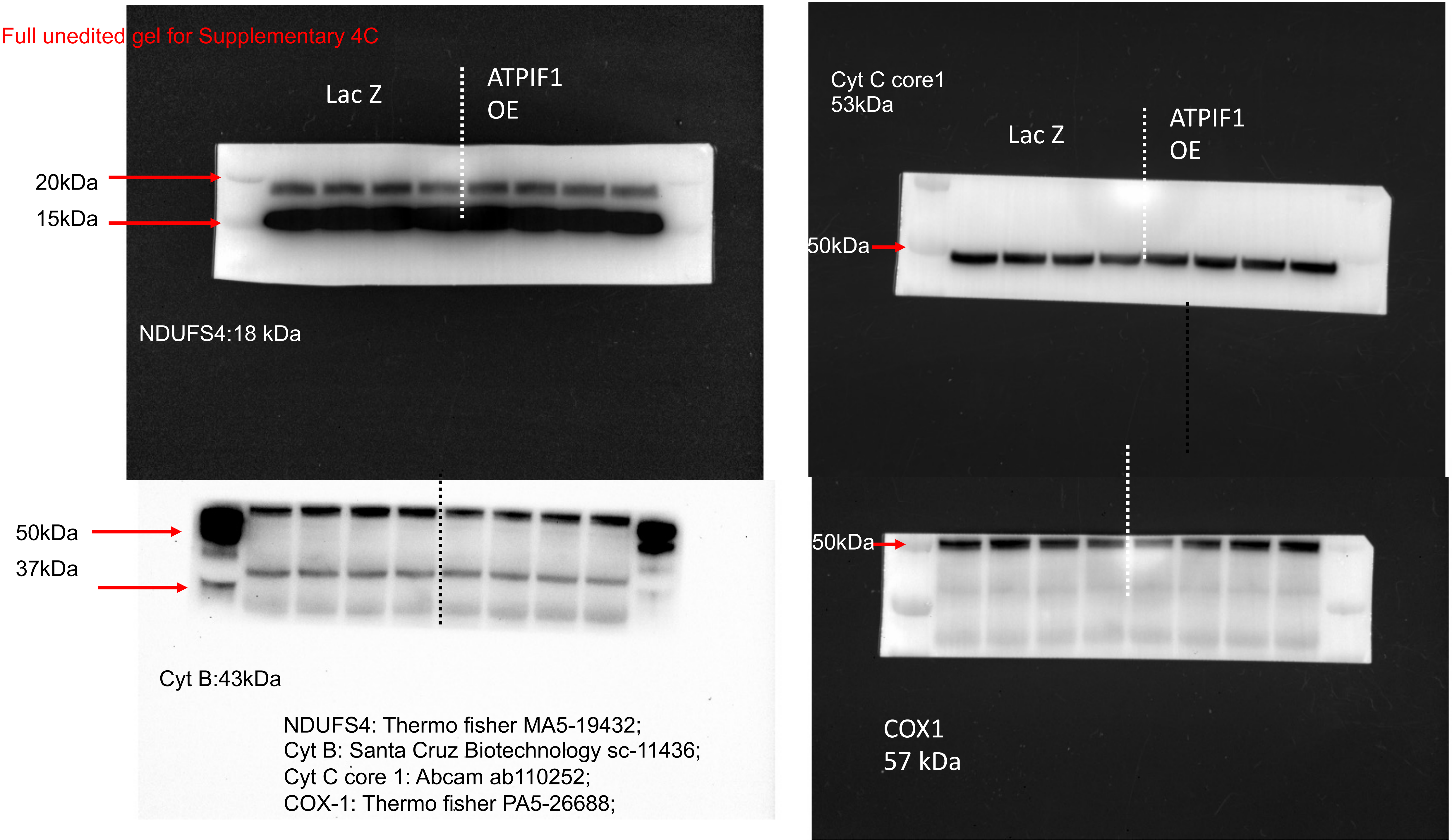

NDUFS4: Thermo fisher MA5-19432;
Cyt B: Santa Cruz Biotechnology sc-11436;
Cyt C core 1: Abcam ab110252;
COX-1: Thermo fisher PA5-26688;

Full unedited gel for Supplementary 4C

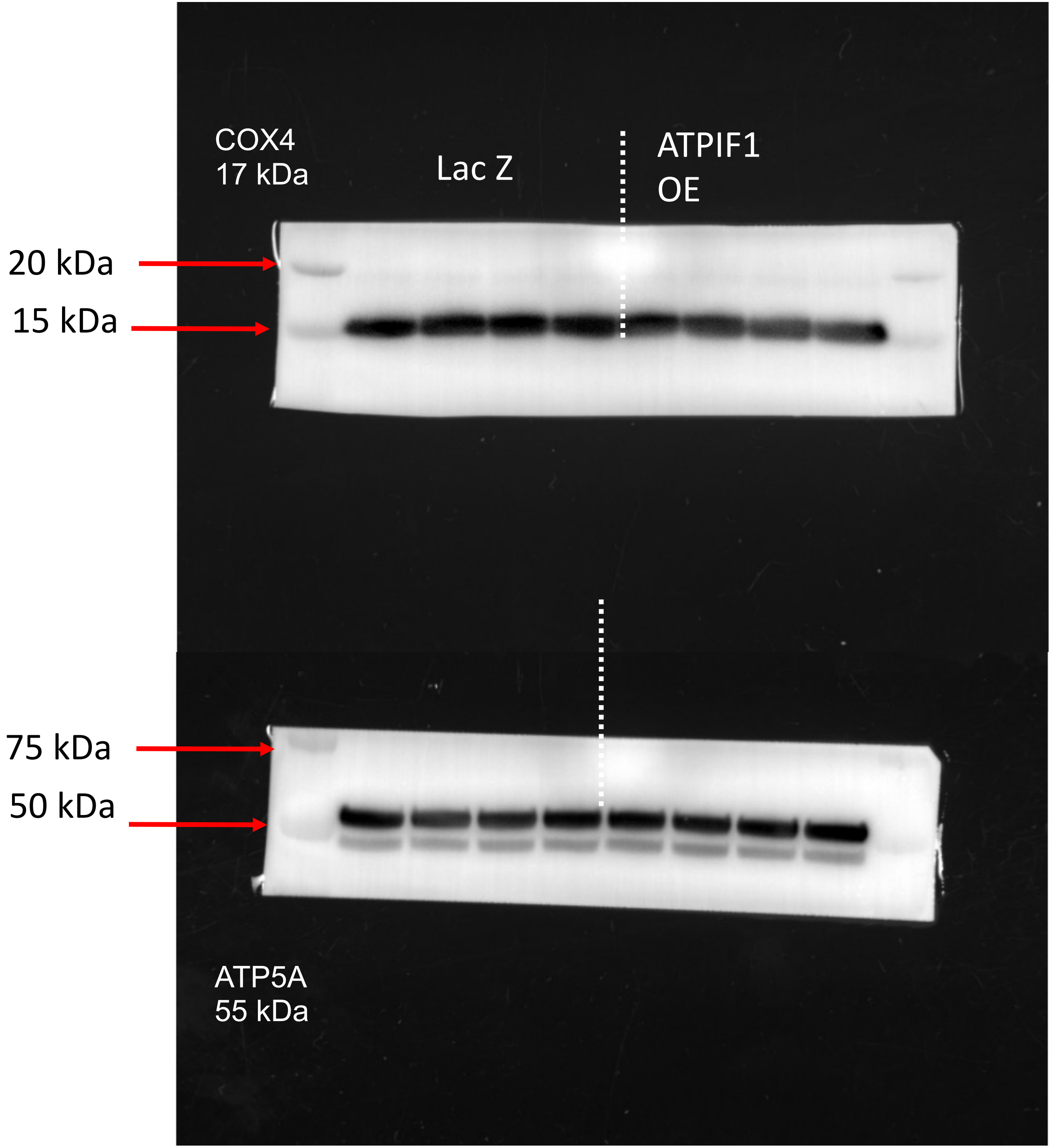

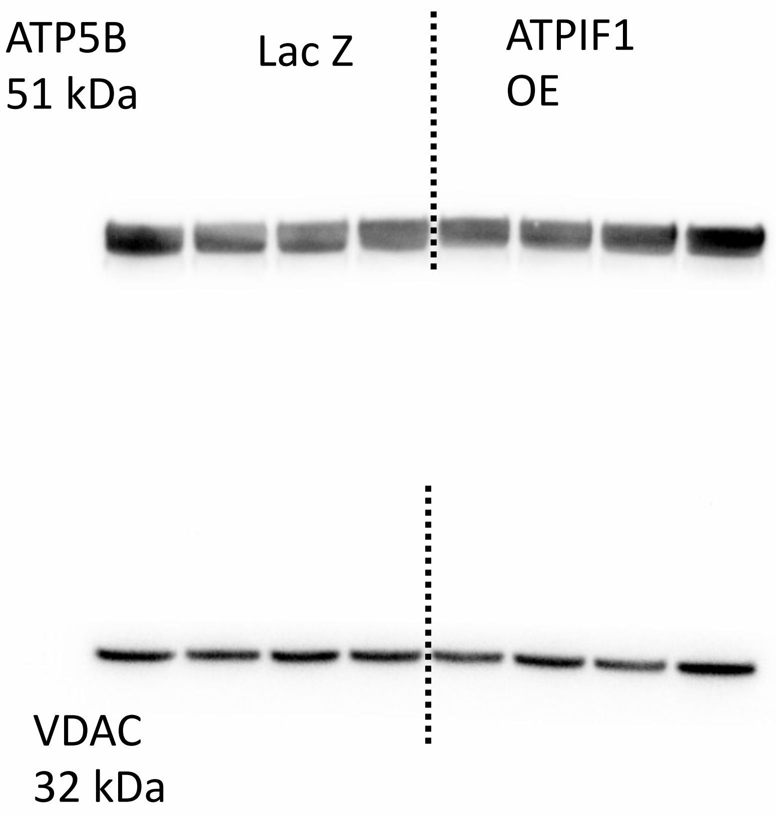

COX 4: Abcam ab14744;
ATP5A: Santa Cruz Biotechnology sc-136178
ATP5B: Santa Cruz Biotechnology sc-33618
VDAC: Cell Signaling Technology 4661

Full unedited gel for Supplementary 4D

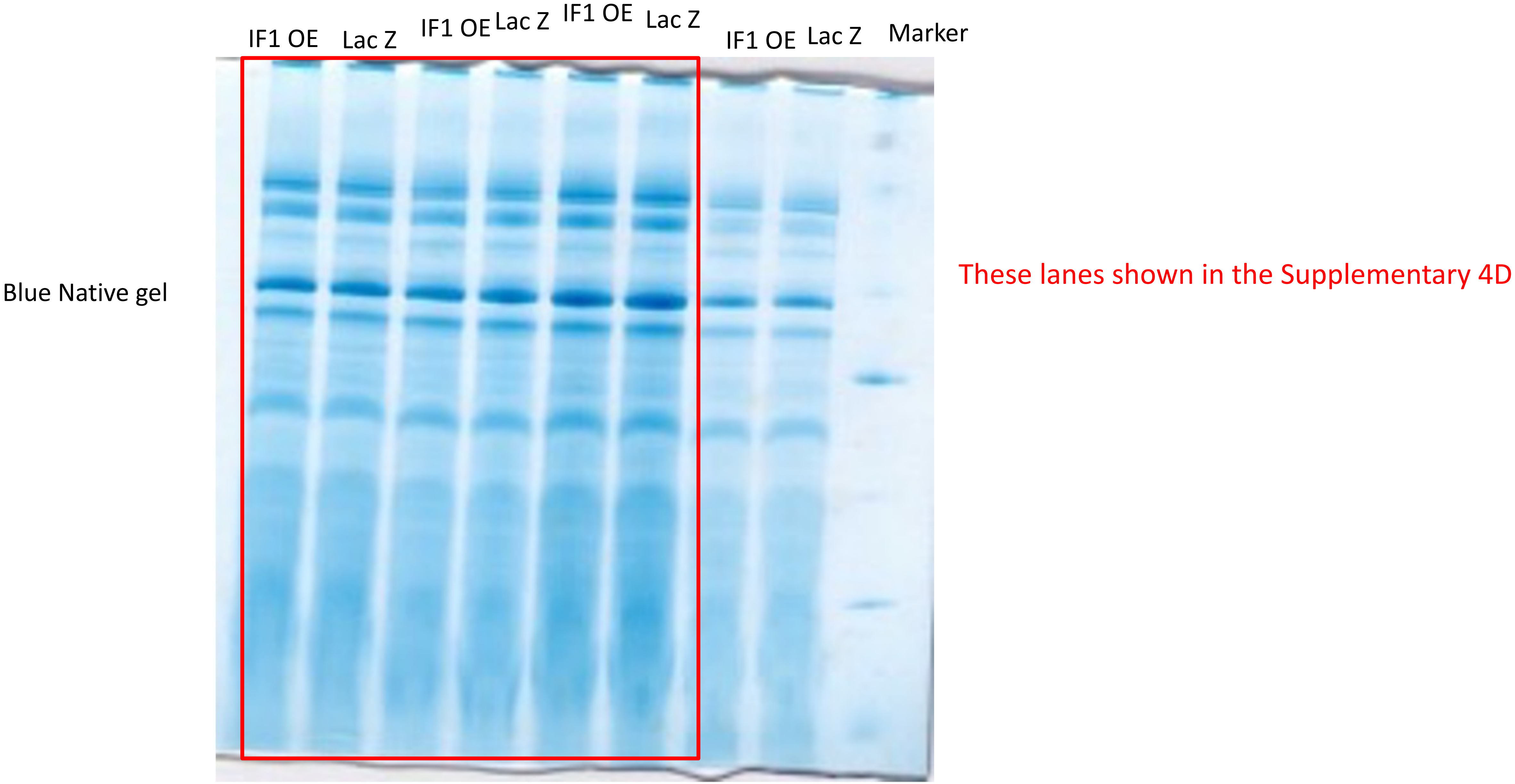

Full unedited gel for Supplementary 5C

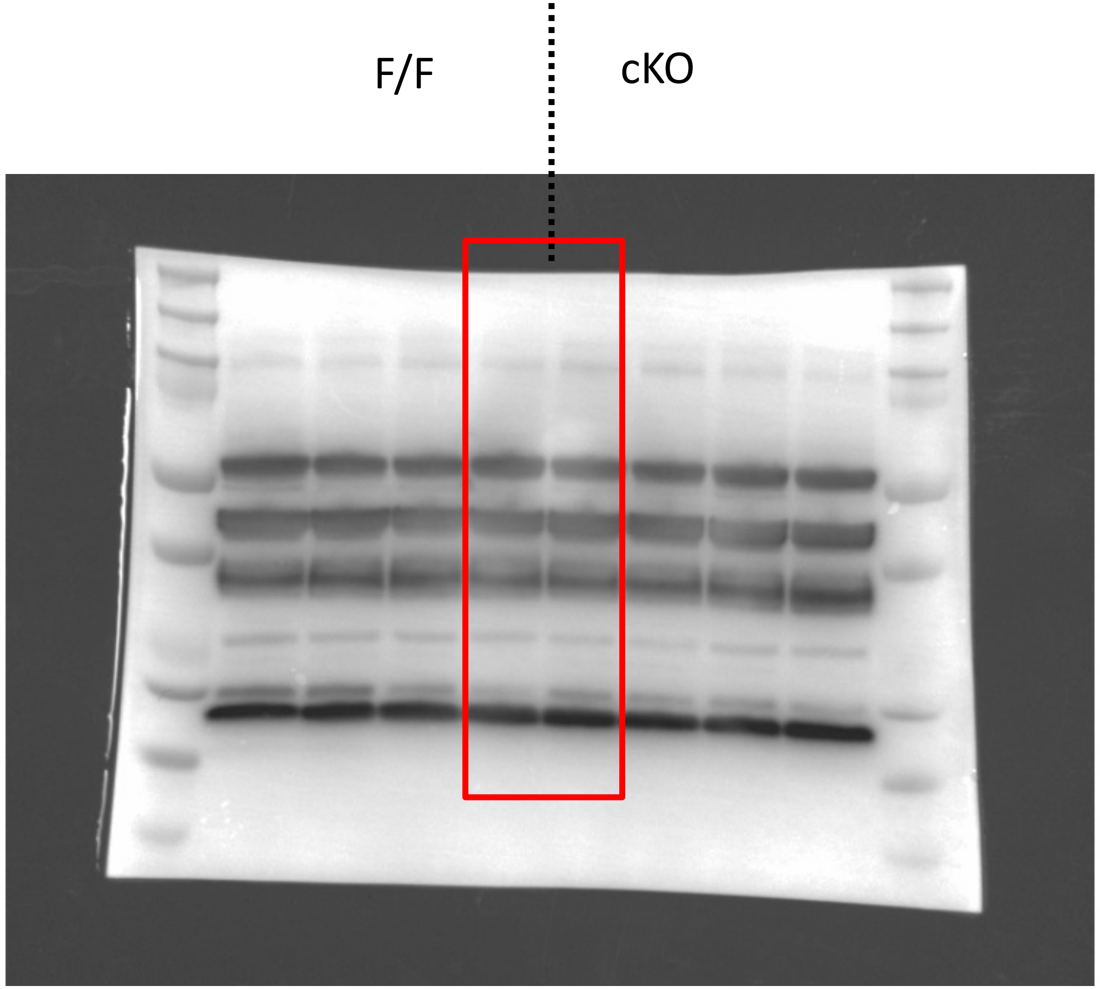

These 2 lanes shown in the Supplementary 5C

OXPHOS cocktail antibody: Abcam ab110413

Full unedited gel for Supplementary 5C

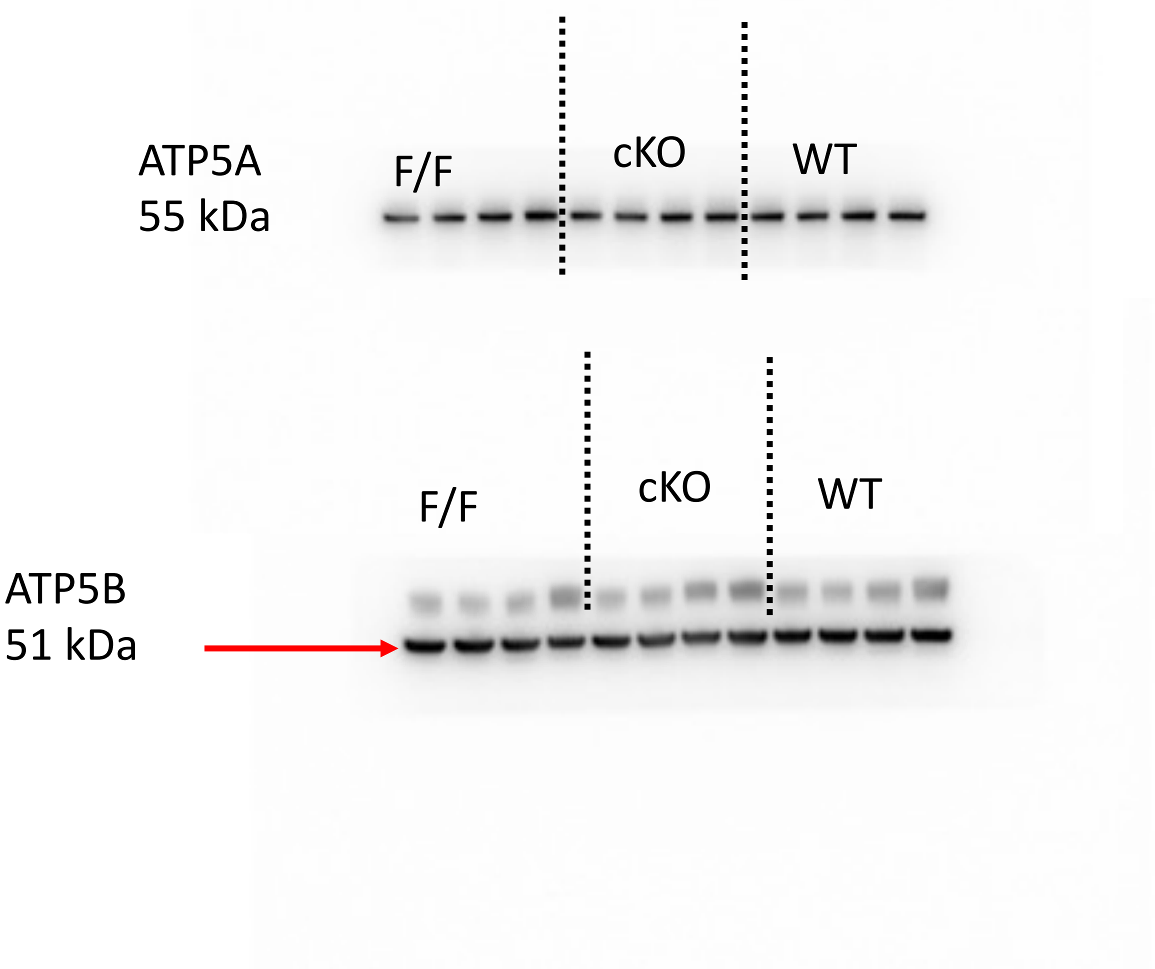

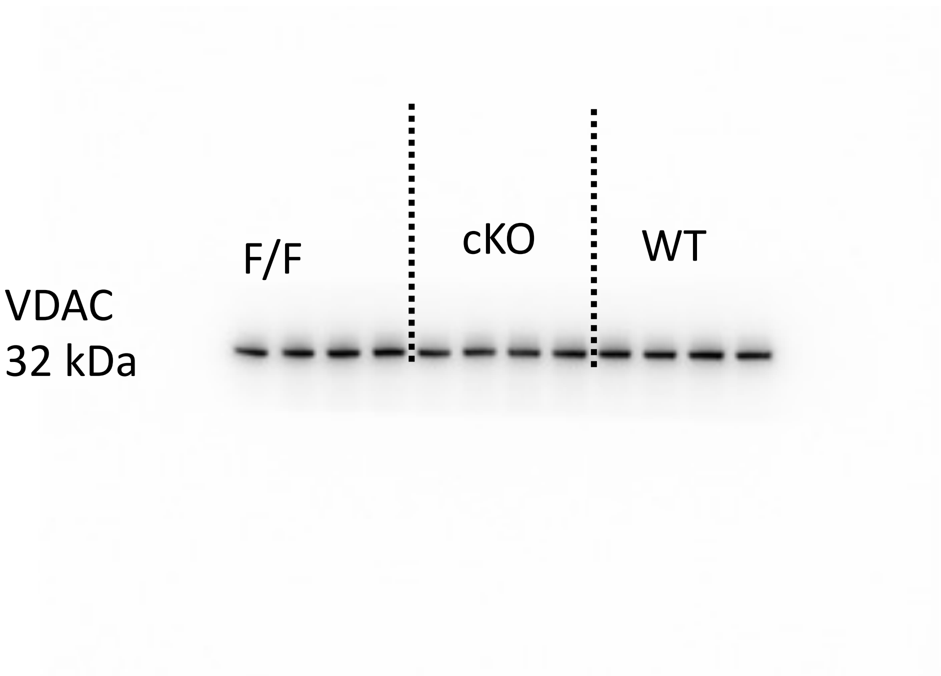

ATP5A: Santa Cruz Biotechnology sc-136178
ATP5B: Santa Cruz Biotechnology (sc-33618)
VDAC: Cell Signaling Technology 4661

Full unedited gel for Supplementary 5D

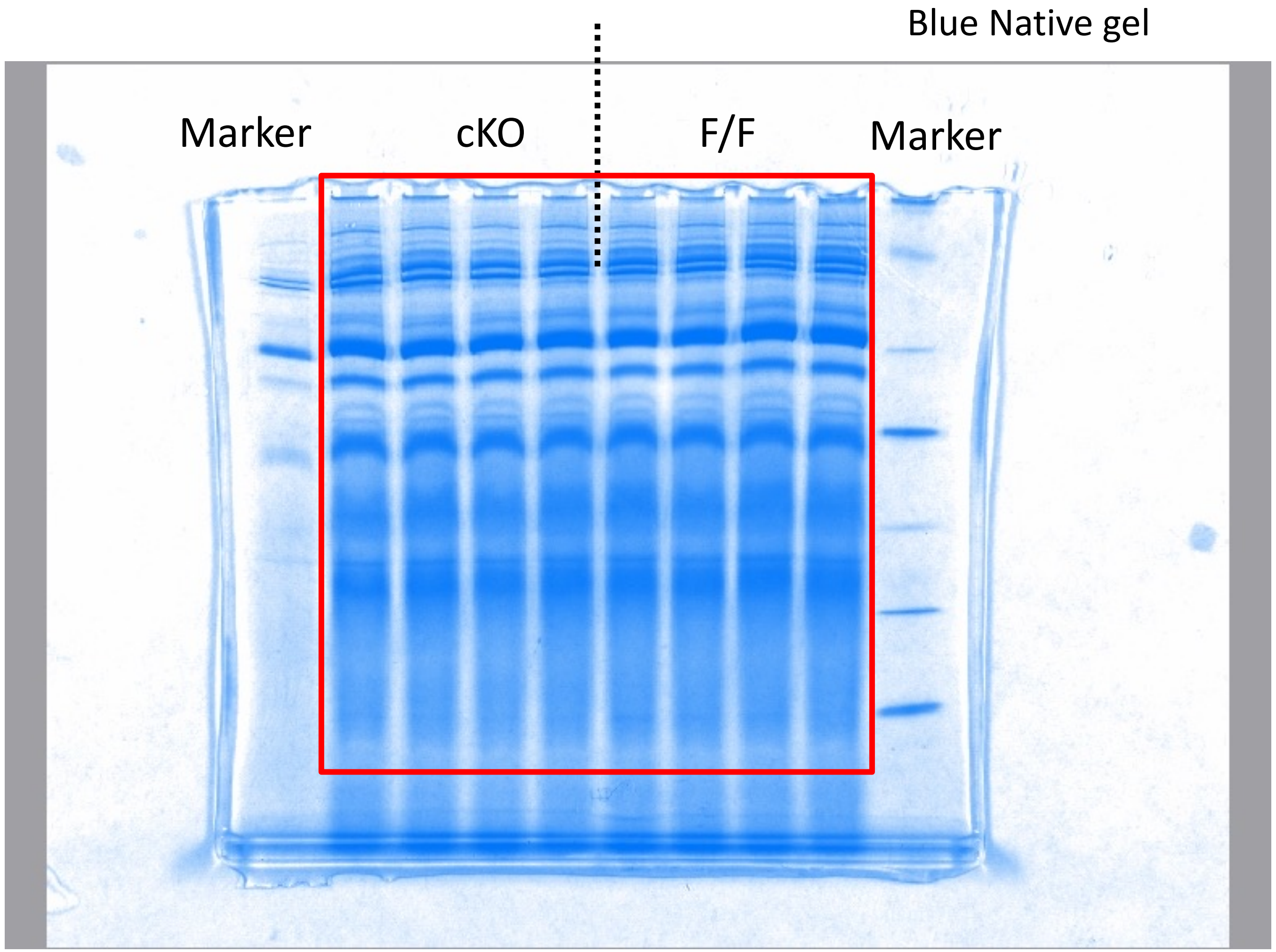

These lanes shown in the Supplementary 5D

Full unedited gel for Supplementary 5E

F/F: Lane 1,3,5,7,9,11;
cKO: 2,4,6,8,10,12

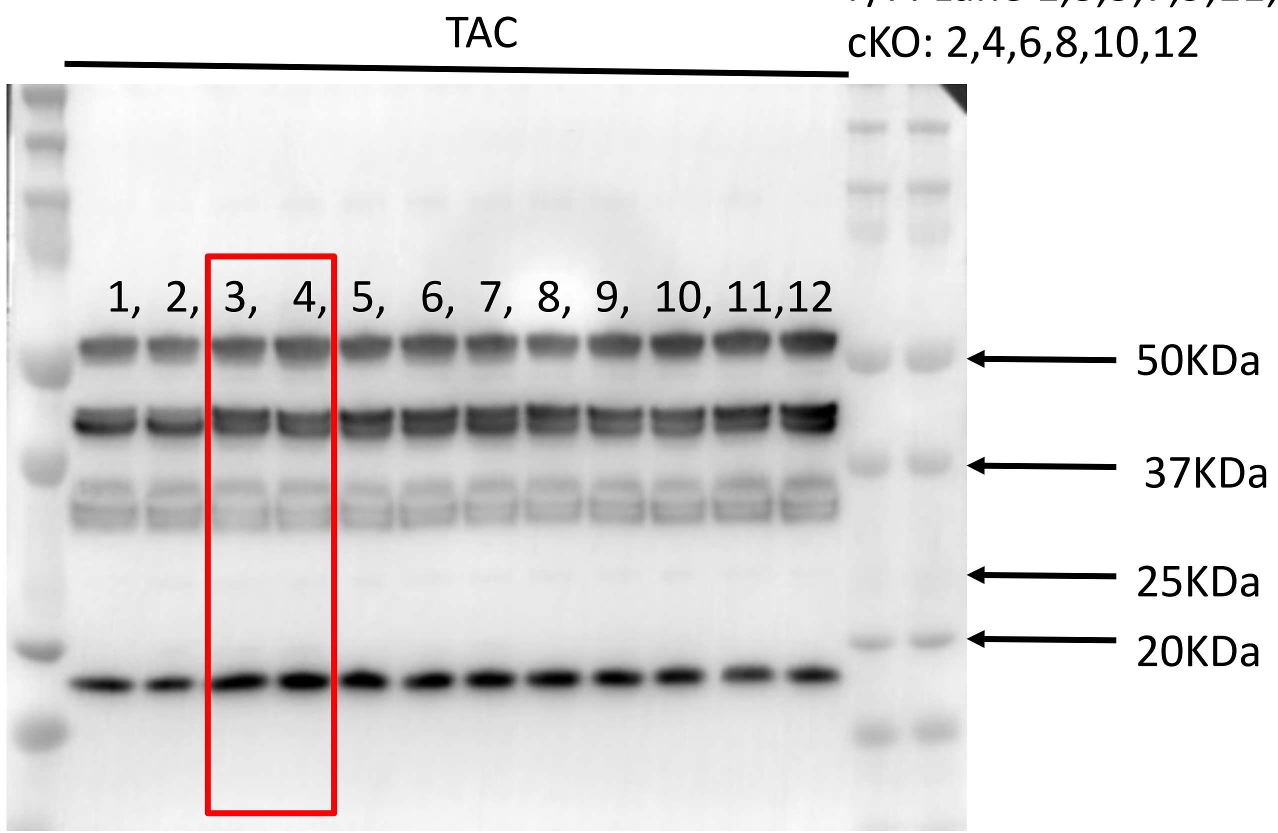

- Complex I subunit NDUFB8 - "CI-20" ~ 20kD
- Complex II subunit 30kDa - "CII-30" ~ 30kD
- Complex III subunit Core 2 - "CIII-core2" ~ 47kD
- Complex IV subunit I - "CIV-I" ~ 39kD
- ATP synthase subunit alpha - "CV-alpha" ~ 53kD

These 2 lanes shown in the Supplementary 5E

OXPHOS cocktail antibody: Abcam ab110413

Full unedited gel for Supplementary 5E

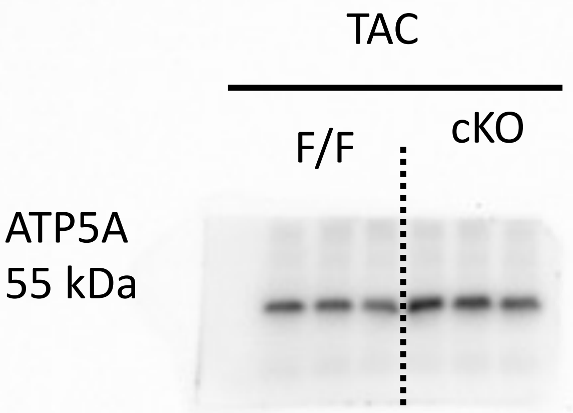

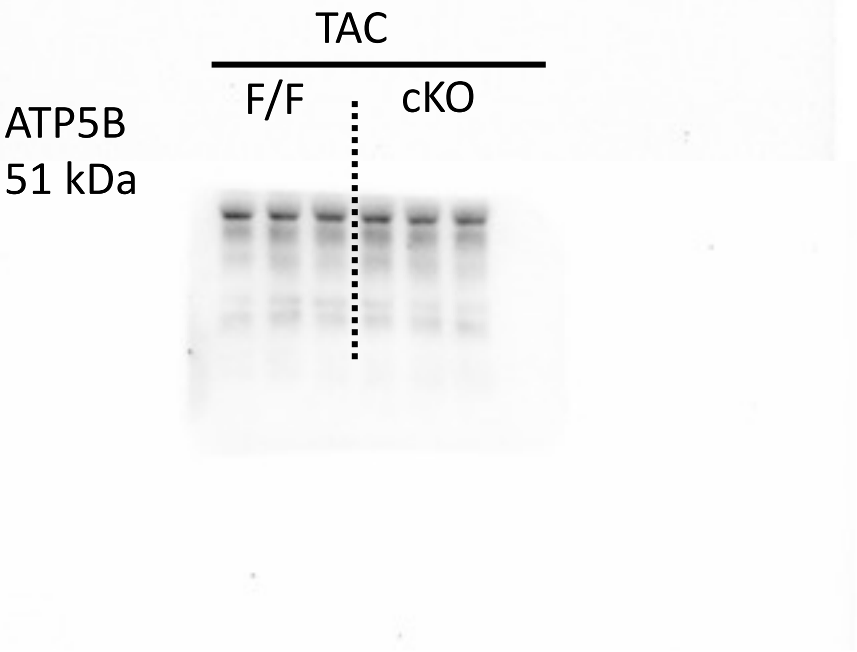

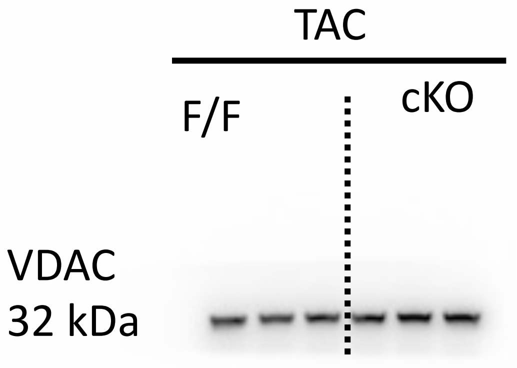

ATP5A: Santa Cruz Biotechnology sc-136178
ATP5B: Santa Cruz Biotechnology (sc-33618)
VDAC: Cell Signaling Technology 4661

Full unedited gel for Supplementary 5F

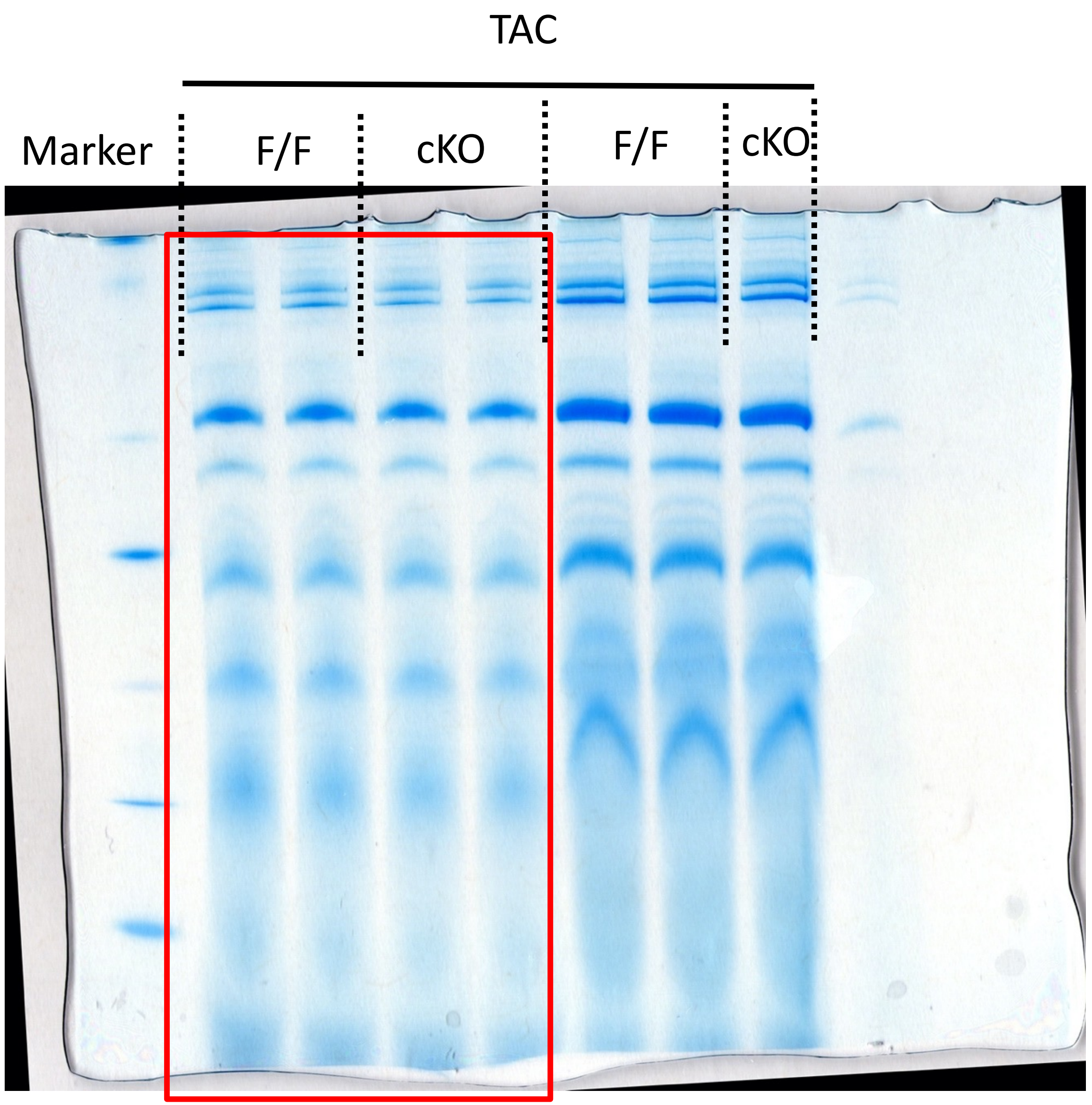

These lanes shown in the Supplementary 5F

Full unedited gel for Supplementary 6E

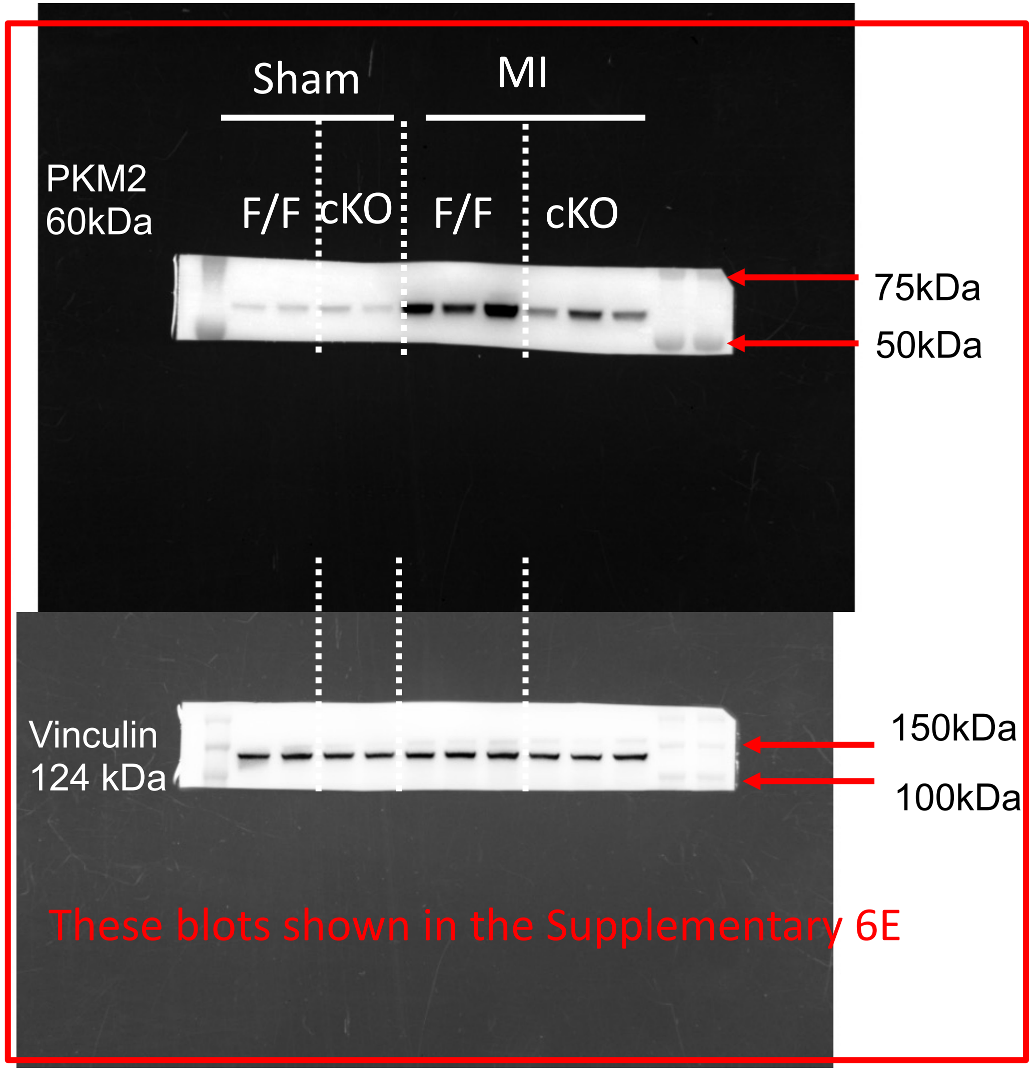

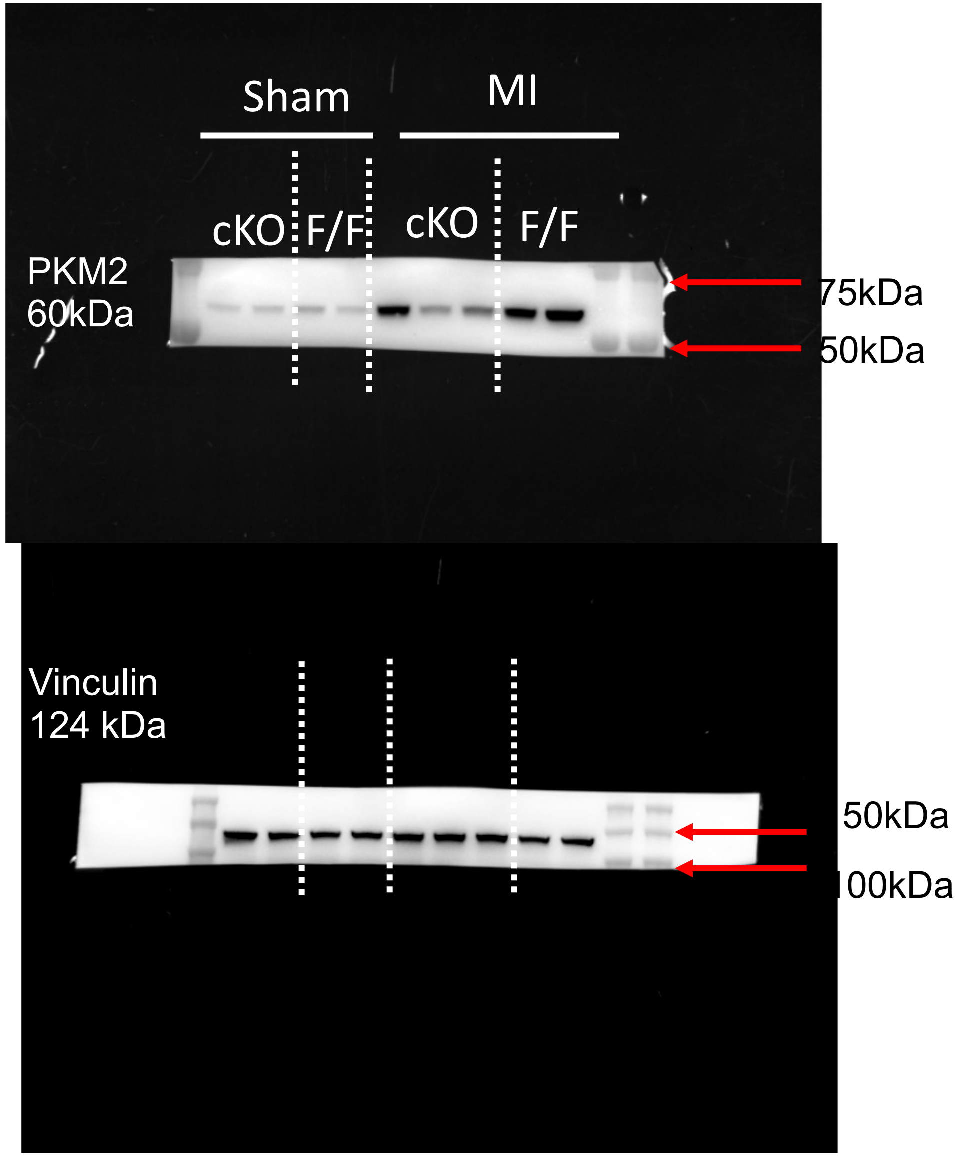

PKM2: Cell Signaling Technology 4053
Vinculin: Cell Signaling Technology 13901

Full unedited gel for Supplementary 6E

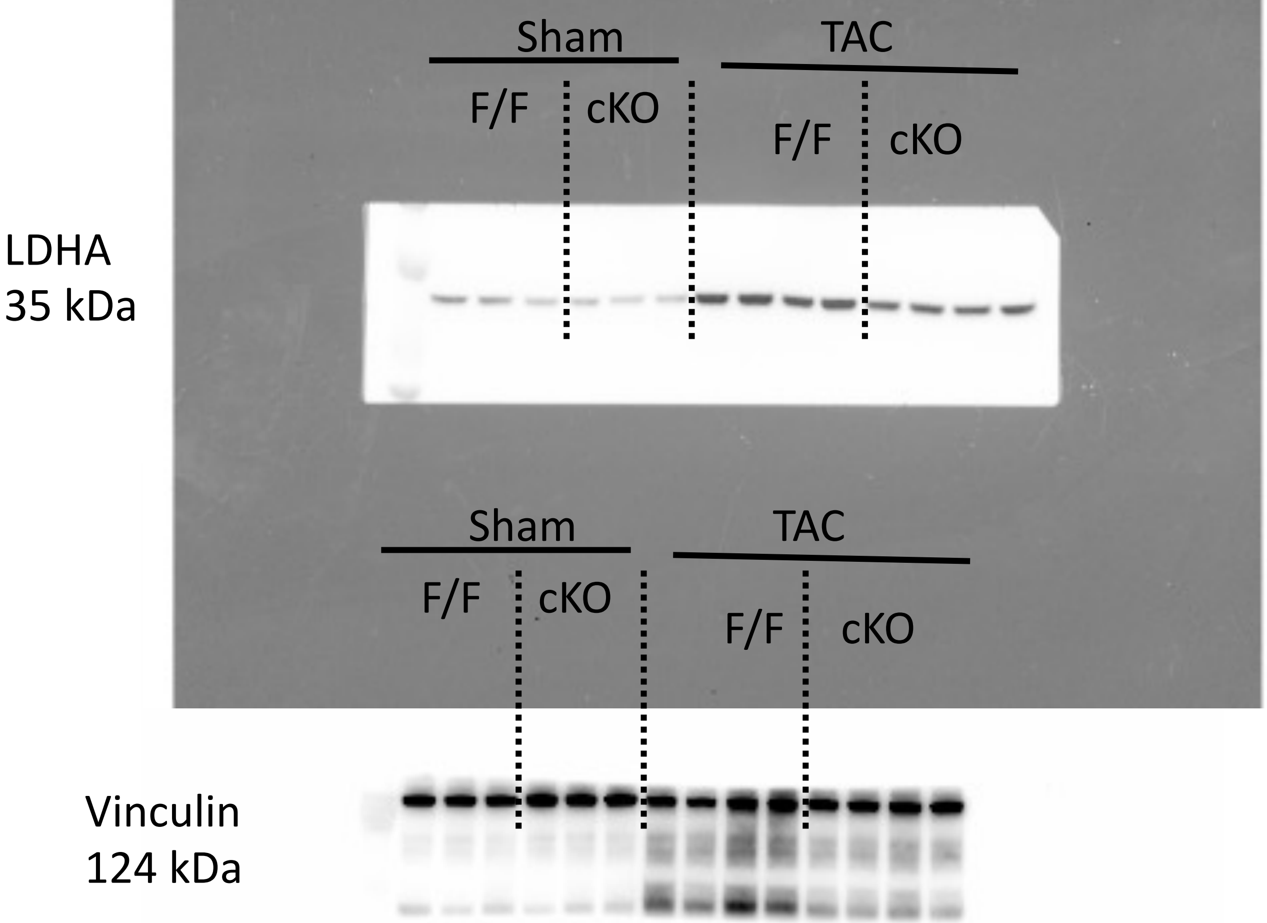

LDHA: Cell Signaling Technology 2012;
Vinculin: Cell Signaling Technology 13901
